# Intelligent design breaks the trade-off between energy efficiency and water flux in ultrafast seawater desalination

**DOI:** 10.1016/j.xinn.2026.101262

**Published:** 2026-01-07

**Authors:** Jiu Luo, Xing Liu, Jin Wang, Yi Heng

**Affiliations:** 1 School of Future Science and Engineering, Soochow University, Suzhou 215222, China; 2 Key Laboratory of General Artificial Intelligence and Large Models in Provincial Universities, Soochow University, Suzhou 215222, China; 3 School of Computer Science and Engineering, Sun Yat-sen University, Guangzhou 510006, China

**Keywords:** ultrapermeable membrane, seawater desalination, batch reverse osmosis, intelligent design, mass transfer enhancement

## Abstract

Enhancing the energy efficiency for energy-intensive seawater desalination technologies is imperative to sustainably mitigate water scarcity while reducing carbon footprints. This work presents a transformative advance in reverse osmosis desalination technology by fundamentally redefining the long-standing trade-off between energy efficiency and water production efficiency. Through the synergistic integration of bio-inspired ultrapermeable membrane module with state-of-the-art batch reverse osmosis, we demonstrate unprecedented performance—achieving a specific energy consumption of 1.68 kWh m^−3^ while delivering an average water flux of 95 L m^−2^ h^−1^. This represents a 33%–58% reduction in energy demand and a 5-fold improvement in water flux compared with conventional seawater reverse osmosis desalination plants (2.5–4 kWh m^−3^ and 15 L m^−2^ h^−1^), challenging the prevailing assumption that increased membrane permeability offers only marginal efficiency benefits. This work can further guide the development of advanced membrane materials and energy-efficient desalination technologies, with potential applications in desalination and zero/minimal liquid discharge systems.

## Public summary

• A multiscale intelligent design framework is proposed for ultrafast seawater reverse osmosis desalination.
• A bio-inspired module is optimized to achieve a best trade-off between mass transfer and flow resistance.
• Optimized ultrapermeable membrane systems significantly improve desalination energy efficiency.

## Graphical abstract

**Figure:**
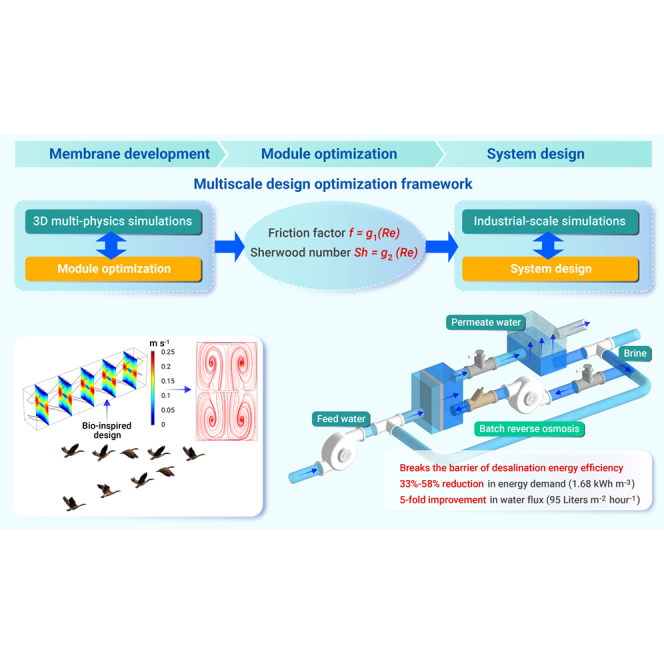

## Introduction

Global freshwater resources are becoming increasingly scarce. Desalination and wastewater reclamation and reuse through purification are expected to be the primary water resource augmentation technologies in the coming decades.^1^ Over the past three decades, seawater desalination capacity has increased more than 8-fold, now reaching 100 million cubic meters per day.^2^ Reverse osmosis (RO) is one of the most advanced desalination technologies currently available, accounting for over 60% of the global desalination market.

The specific energy consumption (SEC) for typical seawater RO (SWRO) systems in engineering (feed salinity 35 g/L, recovery rate 50%) ranges from 2.5 to 4.0 kWh m^−3^ which amounts to 71% of the total plant energy consumption.^3^ The rest of the energy consumption distribution is as follows: 11% is used for pre-treatment, 5% for delivering product water, 5% for intake, and 8% for other facilities.^3^ The theoretical SEC is approximately 1.1 kWh m^−3^ under the same feed salinity and recovery rate conditions. The comprehensive evaluation of SEC in SWRO systems indicated that it enables energy savings of approximately 69% through technological improvements in, e.g., pump efficiency, membrane permeability, mass transfer ability in membrane channel, and pre- and post-treatment.^4^ Further implementation of innovative system configurations, such as batch SWRO, could yield energy savings of up to 82%.^4^ The potential of further energy consumption reduction is even greater in zero/minimal liquid discharge systems.^5^

Ultrapermeable membrane (UPM) materials such as graphene/graphene oxide,^6^^,^^7^ carbon nanotube,^8^^,^^9^^,^^10^ improved polyamide,^11^^,^^12^^,^^13^ aquaporins,^14^^,^^15^^,^^16^ and fluorous nanochannels^17^ have undergone rapid development in recent years, with membrane permeabilities significantly improved over commercial thin-film composite SWRO membranes. For example, the developed biomimetic membranes can achieve a high water flux of 75 L m^−2^ h^−1^ (lmh) for SWRO (feed salinity of 35,000 ppm) with a high salt rejection (99.5% for sodium chloride or 91.4% for boron).^18^ The large-area graphene-nanomesh/single-walled carbon nanotube membrane reported exhibits excellent hydraulic permeability, with a rate of 97.6 lmh bar^−1^ for NaCl solution compared with 110.6 lmh bar^−1^ for pure water.^9^ It also demonstrates a high rejection ratio for salt ions and organic molecules, along with outstanding mechanical strength. In addition to water permeability and salt rejection, membrane development increasingly focuses on improving chlorine resistance, boron rejection, and anti-fouling properties.^19^

However, the applications of next-generation UPMs face several primary challenges. The first challenge lies in scaling up the production of UPMs that maintain an optimal balance between salt rejection, mechanical durability, and cost-effectiveness, particularly when advanced materials such as graphene oxide or aquaporins are employed. The second challenge lies in optimizing the membrane module to achieve a 2- to 5-fold enhancement in the boundary layer mass transfer coefficient.^20^ The experimental results indicate that the mass transfer coefficient with the static mixing spacer is 20% higher than that of the conventional spacer.^21^ The three-dimensional (3D) computational fluid dynamics (CFD) simulations reveal that twisted spacers exhibit a mass transfer (Sherwood number) approximately 55% higher and a friction factor 8% lower compared with conventional spacers.^22^ Using the machine learning-aided optimization approach in our previous work,^23^ the boundary layer mass transfer coefficient for the optimized feed spacer is improved by approximately 21.1%, accompanied by a 23.4% increase in axial pressure drop per meter compared with the commercial non-woven spacer. Therefore, doubling the boundary layer mass transfer coefficient with a moderate flow resistance (or pressure drop) penalty is challenging for typical spiral wound membrane module.^20^

In improving the energy efficiency of seawater desalination, system design offers considerable potential.^24^ In contrast to traditional steady-state RO operating under constant pressure, dynamic RO systems, such as batch^25^^,^^26^^,^^27^ or semi-batch^28^^,^^29^ configurations, operate with time-varying operating pressures. These systems have the potential for energy savings and could provide more uniform water flux distribution, effectively mitigating concentration polarization and membrane fouling. Therefore, UPM in conjunction of innovative membrane module and dynamics RO has the potential to significantly enhance water flux and reduce SEC.

## Materials and methods

### Overview of multiscale design optimization framework

In this work we propose a multiscale optimization framework that integrates membrane permeability, feed spacer design at sub-millimeter scale, and system design (two-stage and batch configurations) at industrial scale (meter scale). A Bayesian-driven pattern search approach is developed for optimal design of feed spacers that is based on the bio-inspired V-shaped spacer proposed in our previous work.^30^ The optimization approach is employed to balance mass transfer and flow resistance and identify optimal geometric parameters for solving a 3D multi-physics constrained optimization problem, incorporating nonlinear channel flow and mass transport. The two-stage and batch SWROs are designed for maximizing the benefit by reducing SEC and enhancing average water flux (or reducing the required membrane area). The 3D multi-physics models are solved with COMSOL Multiphysics 5.3a, whereas the spacer optimization and system design are conducted in MATLAB.

### Optimal membrane module design

#### Optimization problem description

The mathematical formulation of the module design optimization problem can be described as follows:

(Equation 1) $$\begin{array}{l} \min_{\beta_{1}}\;F_{1}\\ s.t.\;H_{1}=0\text{.} \end{array}$$

Design parameters (β_1_) of the membrane module, including distance parameters, size parameters, and a shape parameter, are expressed as β_1_ = [*L*_S_, *W*_S_, *a*_1_, *W*_1_, *H*_1_, *H*_2_] for cosine-shaped, β_1_ = [*L*_S_, *W*_S_, *a*_2_, *W*_1_, *H*_1_, *H*_2_] for parabolic-shaped, and β_1_ = [*L*_S_, *W*_S_, *α*, *W*_1_, *H*_1_, *L*_1_] for V-shaped membrane modules, as shown in Figure S1. The objective function (*F*_1_) is mathematically defined as follows:

(Equation 2) $$F_{1}=\frac{APLR}{{\left({\bar{k}}_{m}/{\bar{k}}_{m,0}\right)}^{\beta }}+\lambda \left(e^{0.1APLR}-1\right)\text{,}$$

where *APLR* represents the ratio of the axial pressure drops per meter between the designed membrane module in this work and the commercial membrane module, mathematically defined as follows:

(Equation 3) $$APLR=\frac{\Delta P_{c}/L}{\Delta P_{c,0}/L_{0}}\text{,}$$

The trade-off parameter *β* is applied to balance mass transfer and pressure drop (or flow resistance). Our previous study^30^ showed that an increase in *β* leads to an improvement in the membrane module’s mass transfer coefficient. However, this improvement is offset by a rapidly increasing pressure drop, which reduces the overall benefit of the enhanced mass transfer. In this study, we introduce a penalty term in Equation 2 with the parameter *λ* = 0.05 to prevent excessive increases in pressure drop. A hybrid algorithm combining Bayesian optimization and pattern search is developed to solve the 3D multi-physics model constrained optimization problem, integrating nonlinear channel fluid flow and mass transport, for the purpose of optimizing the geometric structure of the membrane module. A more detailed description of the hybrid algorithm can be found in Bayesian-driven pattern search.

#### Bayesian-driven pattern search

Bayesian optimization, a well-established technique, serves as a quintessential example of global black-box optimization algorithms,^31^ which has been widely applied across various fields, such as reactor designs,^32^ inverse heat transfer problems,^33^ and parameter estimation of partial differential equation models.^34^ The objective functions it seeks to optimize often lack fundamental mathematical properties, such as convexity and differentiability, and their evaluation is generally computationally expensive. The Bayesian algorithm assumes a model for the unknown objective function before optimization, with Gaussian processes commonly used due to their consistency over compact sets and closed-form posterior distribution. It iteratively selects the point with the highest acquisition function value.

The concept of pattern search was first introduced by Hooke and Jeeves in 1961,^35^ and later Kolda et al. developed a unified framework, demonstrating its strong adaptability in addressing complex engineering problems.^36^ A key advantage of pattern search algorithm is its independence from derivative information, making it particularly well suited for engineering optimization tasks. The algorithm iteratively executes search and polling steps based on the pattern vector direction and current grid size until the predefined convergence criteria are satisfied.

The developed hybrid optimization approach integrates the Bayesian algorithm with pattern search, offering enhanced robustness and computational efficiency. Its capabilities make it particularly well suited for engineering optimization challenges that involve costly computational simulations. For further details on Bayesian optimization and the pattern search algorithm, please refer to Note S1.

### Optimal design of two-stage UPM systems

#### Optimization problem description

The optimal design of two-stage UPM systems can be formulated as

(Equation 4) $$\begin{array}{l} \min_{\beta_{2}}\;F_{2}\\ s.t.\;H_{2}=0\text{,}\\ J\leq 0\text{.} \end{array}$$

The optimal design parameters (β_2_) are determined by minimizing the objective function (*F*_2_) subject to system-level model constraints and inequality constraints (**J** ≤ **0**) including limits on concentration polarization factor (CPF), maximum allowable average permeate salinity, minimum required water flux, and the specified range for design variables. *F*_2_ represents a balance between the annualized capital cost of the membrane and the energy cost per cubic meter of permeate, formulated as follows.

(Equation 5) $$F_{2}=\frac{A_{\text{tot}}c_{m}F_{a}}{Q_{p}t_{\text{op}}}+c_{e}\cdot \mathrm{SEC}\text{.}$$

This work employs the membrane cost per square meter (*c*_m_) as a control variable to balance the trade-off between SEC and the total required membrane area (*A*_tot_). The parameters *c*_e_, *F*_a_, and *t*_op_ correspond to the energy cost per kilowatt-hour, the annual amortization factor, and the total operating hours per year, respectively, with their values sourced from previous research.^37^ The SEC for two-stage RO is calculated by

(Equation 6) $$\mathrm{SEC}=\frac{Q_{0}\Delta P_{0}+Q_{1}\left(P_{1,\text{in}}-P_{1,\text{out}}\right)-\eta_{R}Q_{2}\Delta P_{2}}{36\eta_{\text{pump}}Q_{p}}\text{,}$$

where *Q*_0_, *Q*_1_, and *Q*_2_ represent the flow rates at the inlet of the first stage, the outlet of the first stage, and the outlet of the second stage, respectively. Similarly, Δ*P*_0_, Δ*P*_1_, and Δ*P*_2_ denote the transmembrane pressures at the corresponding locations. The hydraulic pressures at the inlet of the second stage and the outlet of the first stage are given by *P*_1, in_ and *P*_1, out_, respectively. The permeate flow rate is denoted as *Q*_p_. In this study, the pump efficiency and energy recovery device efficiency for the seawater SWRO system are specified as 85% and 95%, respectively.

The design parameters at system-level include water permeability (*L*_P_) and salt permeability (*B*), number of pressure vessels (*N*_pv, 1_, *N*_pv, 2_), number of modules per vessel (*n*_mem, 1_, *n*_mem, 2_), transmembrane pressures (Δ*P*_0_, Δ*P*_1_) at first and second stages, respectively, and number of spacer sheets per module (*n*_sp_). To obtain the optimal system configuration, the optimization problem (4) is solved as a nonlinear mixed-integer programming problem. The optimization process is conducted using the genetic algorithm toolbox, GATBX.^38^ Further details on the system model are available in our previous work.^23^

### Optimal design of batch UPM systems

The normalized SEC (NSEC) considering concentration polarization and expressed as SEC/*π*_0_ (where *π*_0_ represents the feed osmotic pressure) is estimated as follows^29^

(Equation 7) $${\text{NSEC}}_{\text{CP}}=\left(\text{CPF}-1\right)\left[-\frac{\ln\left(1-Y_{\text{tot}}\right)}{Y_{\text{tot}}}-\ln\left(1-Y_{\text{tot}}\right)\left(\frac{1}{f_{n_{\text{mem}}}}-1\right)\right]\text{.}$$

The total recovery rate is represented by *Y*_tot_, while $f_{n_{\text{mem}}}$ is the flushing efficacy when *n*_mem_ RO modules are arranged in series. $f_{n_{\text{mem}}}$ can be estimated using the simulated results of a 3D transient CFD model in a five spacer-filled channel incorporating convolution operation.^39^ CPF can be estimated by $\exp\left({\bar{J}}_{W}/{\bar{k}}_{m,\text{per}}\right)$, where ${\bar{J}}_{W}$ and ${\bar{k}}_{m,\text{per}}$ denote the average water flux and the estimated cell-averaged mass transfer coefficient using permeable wall model, respectively. ${\bar{k}}_{m,\text{per}}$ can be converted by the empirical correlation^40^ incorporating ${\bar{k}}_{m}$ calculated by CFD with the use of an impermeable wall boundary condition. The NSEC associated with frictional losses can be estimated as follows^29^

(Equation 8) $${\text{NSEC}}_{\text{friction}}=\frac{\alpha_{2}}{\pi_{0}}\frac{\frac{1}{t_{1}+1}\frac{1-{\left(1-Y_{\text{SP}}\right)}^{t_{1}+1}}{Y_{\text{SP}}}+\frac{Y_{\text{SP}}\left(1-Y_{\text{tot}}\right)}{Y_{\text{tot}}}}{Y_{\text{SP}}}\text{,}$$

The single-pass recovery is denoted as *Y*_SP_. The system-level pressure drop (*α*_2_) can be determined by the relations $-\Delta P_{c}/L=k_{1}Q^{t_{1}}$, where the parameters *k*_1_ and *t*_1_ are derived through regression analysis of CFD simulation data.

## Results

### Multiscale optimization framework

**Figure 1:**
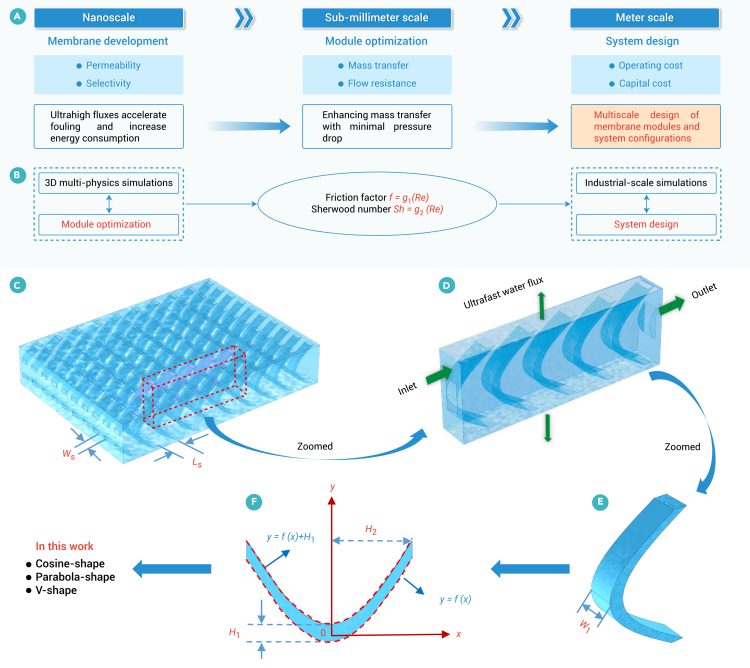
Overview of multiscale design optimization (A) Trade-off between permeability and selectivity for membrane development, mass transfer, and flow resistance for module optimization, and operating cost (i.e., specific energy consumption of this work) and capital cost (i.e., required membrane area of this work) for system design. (B) Multiscale design optimization coupling module optimization (or feed spacer design) at sub-meter scale and system design at meter scale (industrial scale). (C) A small piece of spacer sheet. (D) Computational domain of three-dimensional multi-physics model. (E) A spacer cell. (F) Lateral view of the spacer cell. The feed spacer-filled channel is defined by distance parameters (e.g., *L*_S_, *W*_S_), size parameters (e.g., *H*_1_, *H*_2_, *W*_1_), and shape function (*y* = *f* (*x*)). This work explores three shape functions—cosine, parabolic, and V shaped—along with additional details on the parametric geometric models in Figure S1.

UPM technology is constrained by factors such as concentration polarization and membrane fouling, highlighting the need for redesigned membrane modules (Figure 1A).^41^ Herein, we present a multiscale design optimization framework (Figure 1B) that combines membrane module optimization (or feed spacer design, Figures 1C–1F) with system design (two-stage and batch designs), incorporating UPMs for SWRO desalination. A Bayesian-driven pattern search approach is developed to optimize the membrane module, achieving a best trade-off between mass transfer and flow resistance. The Bayesian method guarantees strong global convergence, aiding in the acceleration of the pattern search approach without the need for derivative information or a well-defined initial estimate. More introduction on the hybrid optimization approach can be found in the materials and methods and Note S1.

In the module design objective function, the trade-off parameter *β* is employed to balance mass transfer and flow resistance. To mitigate excessive flow resistance, a penalty term is introduced in the optimization objective function to penalize pressure drop (Equation 2). It enables the optimal design of the membrane module to maximize mass transfer enhancement while applying an appropriate flow resistance penalty through adjustment *β*. Based on CFD simulations of the optimal membrane module across various Reynolds numbers (*Re*), the relationships between the Darcy friction factor (*f* = *g*_1_ (*Re*)) and Sherwood number (*Sh* = *g*_2_ (*Re*)) with respect to *Re* can be determined (Figure 1B).

In the system design, this work focuses on the operational and capital costs of membrane separation, particularly electricity and membrane expenses in membrane separation. A constant feed flow rate is maintained in our design; therefore, pre-treatment cost is fixed. Capital investment increases in the order of single-stage, batch, and multi-stage RO. Theoretically, batch RO can match the energy efficiency of an idealized infinite-stage RO system, which is economically impractical to construct. Moreover, the system design solutions are obtained (Figure 1B) by solving the system-level model constrained optimization problem (see materials and methods). The optimization objectives in system design include SEC (operating cost) and required membrane area (capital cost), the membrane cost of which per m^2^ (*c*_m_) is considered as a trade-off parameter to balance both objectives. The system design optimization framework, given a specified inlet flow rate and recovery rate, ensures that key constraints are met, including minimum average permeation flux, maximum permeate salinity, and the maximum CPF across the entire system, which is crucial for mitigating fouling and scaling. The two-stage and batch configurations are employed in this work, which reduces inlet operating pressure compared with a standard one-stage SWRO, thereby alleviating flux and CPF on the lead elements. Further details on the optimization framework are presented in the materials and methods and in Notes S2 and S3.

### Innovative membrane module design

**Figure 2:**
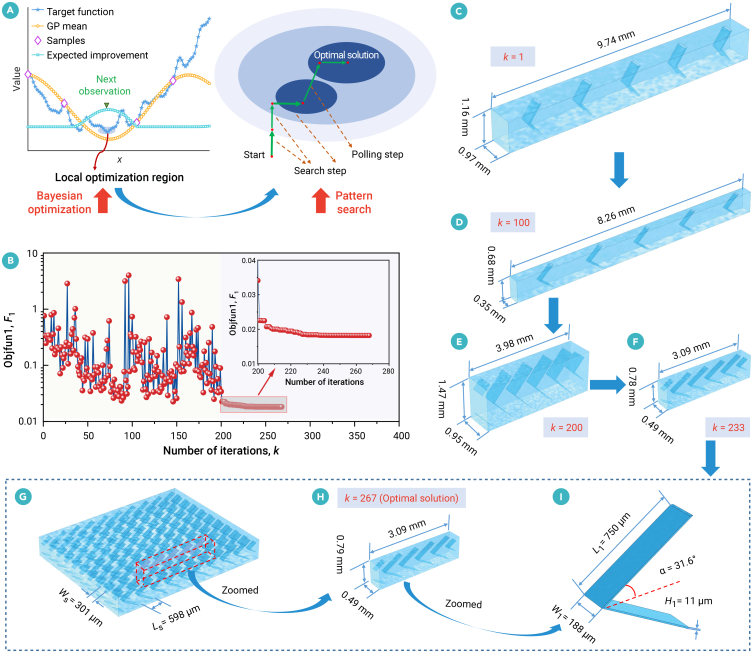
Bayesian-driven pattern search approach and its application (A) The schematic diagram of the hybrid optimization algorithm. (B–I) Convergence curve for optimizing the V-shaped spacer versus the number of iterations (*k*). The optimized solutions for (C) *k* = 1, (D) *k* = 100, (E) *k* = 200, and (F) *k* = 233. The optimal solution (*k* = 267) of (G) a small piece of spacer sheet (*k* = 267), (H) computational domain, and (I) a spacer cell. The optimal solution balances pressure drop and mass transfer with a trade-off parameter of *β* = 7 in the objective function (*F*_1_) for this case. The *F*_1_ is mathematically formulated as shown in Equation 2.

It requires radically different module design for the next-generation UPM system operated at ultrafast water flux, such as more than 100 lmh. Migratory birds—such as pelicans^42^ and geese^43^—fly in V-formations to save energy. Pelicans flying in vortex wakes achieve energy savings of 11.4%–14.0% by gliding longer or reducing wingbeat frequency.^42^ Inspired by this mechanism, three types of spacers—cosine, parabolic, and V shaped—are studied, with their parametric geometric models shown in Figure S1. The ranges of geometric design parameters for cosine-, parabolic-, and V-shaped spacers are provided in Table S1. Furthermore, the proposed Bayesian-driven pattern search approach (Figure 2A) is employed for optimizing the membrane module to balance the trade-off between mass transfer and flow resistance and maximize the benefit. Using this optimization approach, we calculate 30 optimized designs (Table S2) for three spacer shapes (V, cosine, and parabola shaped) across a range of trade-off parameters (*β* = 1, 2, ···, 10).

Taking the optimization process of the V-shaped spacer (*β* = 7), for example, the convergence curve (objective function *F*_1_ versus number of iterations *k*) is shown in Figure 2B. Following Bayesian optimization, the identified optimal point is regarded as a local optimum region and subsequently refined using the pattern search algorithm. Several representative geometric structures for number of iterations, *k* = 1, 100, 200, and 233, are shown in Figures 2C–2F, respectively. The optimal solution (*k* = 267) for the feed spacer is shown in Figures 2G–2I. Using the Bayesian-driven pattern search algorithm, the optimized geometric parameters and the Sherwood numbers with respect to various Reynolds numbers, along with the repeatability test, are shown in Tables S3 and S4, respectively. Despite the stochastic nature of the Bayesian-based algorithm, the two independent calculations yielded highly consistent results, with relative deviations in the Sherwood numbers of less than 4%, demonstrating the optimization framework’s replicability. Furthermore, we conduct sensitivity analysis of design parameter for the optimized objectives of ${\bar{k}}_{m}$/${\bar{k}}_{m,0}$ and $\frac{\Delta P_{c}/L}{\Delta P_{c,0}/L_{0}}$. ${\bar{k}}_{m}$/${\bar{k}}_{m,0}$ denotes the ratio of cell-averaged mass transfer coefficients for the optimized spacer (${\bar{k}}_{m}$) versus the commercial spacer (${\bar{k}}_{m,0}$) while $\frac{\Delta P_{c}/L}{\Delta P_{c,0}/L_{0}}$ is ratio of pressure drops per meter for the optimized spacer (Δ*P*_c_/*L*) versus the commercial spacer (Δ*P*_c,0_/*L*_0_). Parameter sensitivity analysis highlights the effective trade-off between mass transfer and pressure drop for the optimized designs in this work (Table S5).

**Figure 3:**
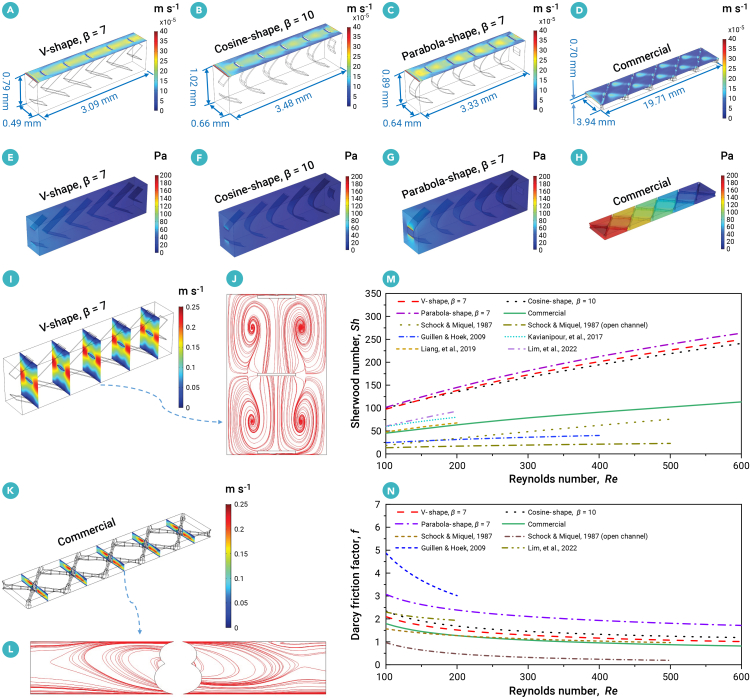
Hydrodynamics and mass transfer characteristics (A–L) Mass transfer coefficient distributions on membrane wall of optimized (A) V-shaped spacer (*β* = 7), (B) cosine-shaped spacer (*β* = 10), (C) parabola-shaped spacer (*β* = 7), and (D) commercial spacer. Pressure profiles of optimized (E) V-shaped spacer (*β* = 7), (F) cosine-shaped spacer (*β* = 10), (G) parabola-shaped spacer (*β* = 7), and (H) commercial spacer. (I) Velocity and (J) streamline distributions in the optimized V-shaped spacer (*β* = 7). (K) Velocity and (L) streamline distributions in the commercial spacer. The cross velocity is set to 0.1 m s^−1^ in (A–L). The Sherwood number and Darcy friction factor correlations for the optimized V-shaped spacer (*β* = 7), cosine-shaped spacer (*β* = 10), parabola-shaped spacer (*β* = 7), and the commercial spacer of this work and previously published correlations. (M) Sherwood number as a function of Reynolds number. (N) Darcy friction as a function of Reynolds number.

The mass transfer coefficients on the membrane wall of the three optimized spacers—V shaped (*β* = 7; Figure 3A), cosine shaped (*β* = 10; Figure 3B), and parabola shaped (*β* = 7; Figure 3C)—which offer the best trade-off between mass transfer and flow resistance, are significantly higher than those of the commercial spacer (Figure 3D). Accordingly, the 3D pressures of the optimized V-shaped (Figure 3E), cosine-shaped (Figure 3F), and parabola-shaped (Figure 3G) spacers are substantially lower than that of the commercial spacer (Figure 3H). This is primarily due to the generation of multi-vortex flow in the optimized channel, e.g., the V-shaped spacer (*β* = 7, Figures 3I and 3J), which results in a significant enhancement of mass transfer compared with the commercial spacer (Figures 3K and 3L). The flow pattern aligns with the theoretically optimal velocity, maximizing heat and mass transfer while accounting for viscous dissipation.^44^^,^^45^ Furthermore, the relationships between the Sherwood number (Figure 3M) and the Darcy friction factor (Figure 3N) as functions of the Reynolds number are utilized to evaluate the fluid mechanics and transport performance for the optimized and commercial spacer in this work and the reported results in previous work.^46^^,^^47^^,^^48^^,^^49^^,^^50^^,^^51^ For a Reynolds number of 100, the Sherwood numbers (*Sh* = 98, 100, and 102) for the optimized V-shaped spacer (*β* = 7), the cosine-shaped spacer (*β* = 10), and the parabola-shaped spacer (*β* = 7) are markedly higher—by 116%, 122%, and 125%, respectively—compared with that of the commercial spacer (*Sh* = 45). In contrast, the Darcy friction factors (*f* = 2.10, 2.34, and 3.07) for the V-shaped spacer (*β* = 7), the cosine-shaped spacer (*β* = 10), and the parabola-shaped spacer (*β* = 7) are elevated by 17%, 31%, and 71%, respectively, relative to the commercial spacer (*f* = 1.79). For a given crossflow velocity of 0.1 m s^−1^, the cell-averaged mass transfer for the optimized V-shaped spacer (*β* = 7), the cosine-shaped spacer (*β* = 10), and the parabola-shaped spacer (*β* = 7) are 2.39, 2.27, and 1.99 times greater than that of the commercial spacer, with corresponding increases in pressure drop per meter of 54%, 76%, and 1%, respectively.

In our previous work,^30^ the V-shaped spacer, inspired by the V-formation of birds, was proposed, achieving a 2.38-fold improvement in cell-averaged mass transfer over the commercial spacer, while accompanied by a pressure drop per meter up to four times higher. Therefore, the optimized results in this work represent a substantial advancement over our previous work.^30^ This improvement is largely attributed to the enhanced performance of the optimization algorithm, which enables the generation of superior solutions. Moreover, compared with the previously used genetic algorithm, the proposed method offers greater computational efficiency, making it feasible to explore a broader range of trade-off parameter values to achieve better solutions. Additionally, refinements in the objective function of this work may also contribute to the improved outcomes. Overall, the optimized V-shaped spacer (*β* = 7) outperforms the other two in balancing mass transfer and flow resistance. The results further validate that the V-shaped migration formation in birds reflects evolutionary intelligence, enhancing energy efficiency.^42^ For a Reynolds number of 100, the Sherwood number (*Sh* = 98) for the optimized V-shaped spacer (*β* = 7) is 2.04, 1.62, and 5.26 times greater than that of the reported results, with corresponding variations in flow resistance of a 57% reduction,^48^ a 9% decrease,^49^ and increases of 34%,^46^ respectively.

**Figure 4:**
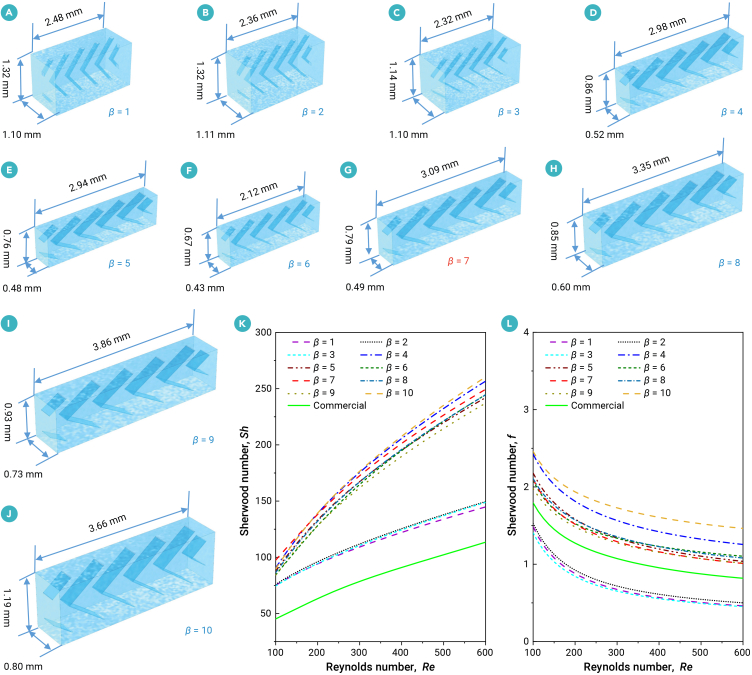
Optimized results for V-shaped spacers Optimized spacer geometries corresponding to different trade-off parameter values: (A) *β* = 1, (B) *β* = 2, (C) *β* = 3, (D) *β* = 4, (E) *β* = 5, (F) *β* = 6, (G) *β* = 7, (H) *β* = 8, (I) *β* = 9, and (J) *β* = 10. Additionally, (K) presents the Sherwood number as a function of Reynolds numbers for various *β* values, while (L) depicts the Darcy friction factor as a function of Reynolds numbers for different *β* values.

The detailed analysis process for exploring the optimal balance between mass transfer and flow resistance across the 30 optimized schemes is outlined as follows. Ratios of cell-averaged mass transfer coefficients and pressure drops per meter using optimized spacers (V, cosine, and parabola shaped) versus those of the commercial spacer are shown in Figure S2. As the crossflow velocity increases, the mass transfer coefficient rises, albeit at the expense of a higher pressure drop. The mass transfer coefficient exhibits an initial increase followed by a decrease as the trade-off parameter *β* increases. This is primarily attributed to the incorporation of a penalty term associated with the pressure drop in the optimization objective function (Equation 2), designed to prevent excessive pressure loss in the optimized results. For a more comprehensive assessment of the optimized results, we further calculate the ratios of the mass transfer coefficients and the pressure drops per meter using the optimized spacers (V, cosine, and parabola shaped) versus those of the commercial spacer, with a cross velocity of 0.1 m s^−1^ (Figures S3A and S3B). The Sherwood number and Darcy friction factor are shown in Figures S3C and S3D, respectively, for a Reynolds number of 100. Obviously, the optimized V-shaped spacer (*β* = 7) achieves the best balance between the mass transfer (or Sherwood number) and pressure drop (or flow resistance). Moreover, the optimized spacer geometries are shown in Figures 4A–4J, respectively, corresponding to different trade-off parameter values (for *β* = 1, 2, ···, 10). Accordingly, the calculated Sherwood numbers versus various Reynolds numbers are shown in Figures 4K and 4L. The additional enhancement in mass transfer for *β* = 7 is constrained compared with *β* = 4 and *β* = 10, whereas its flow resistance remains significantly lower than those of *β* = 4 and *β* = 10. Overall, the optimized V-shaped spacer with a trade-off parameter of *β* = 7 is recognized as the optimal configuration for further system design.

### Optimal design of two-stage UPM SWRO system

**Figure 5:**
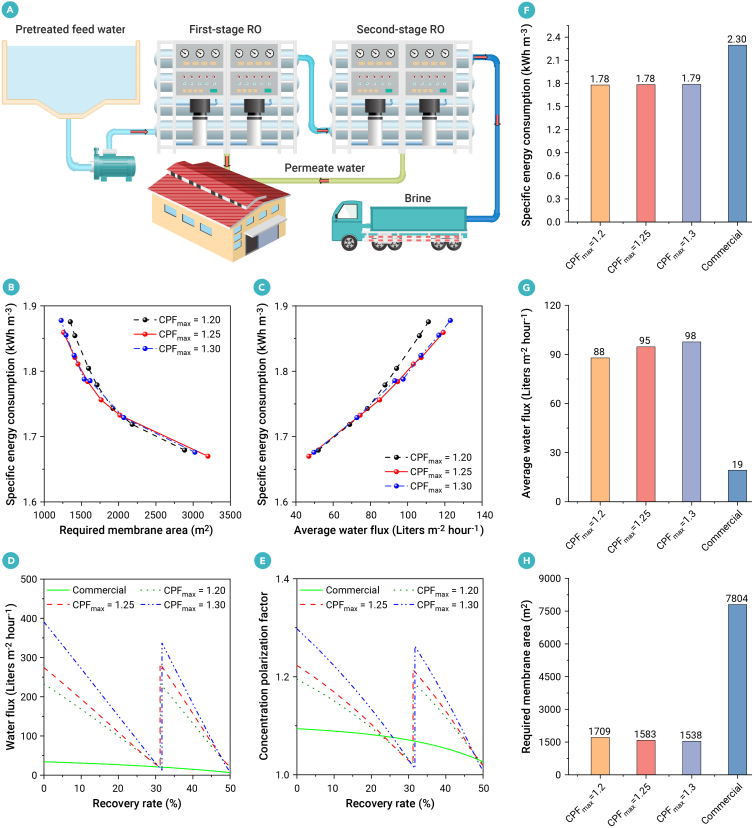
Optimal design of two-stage ultrapermeable reverse osmosis systems (A) Process flow diagram for two-stage reverse osmosis (RO). (B) Pareto front optimization between specific energy consumption (kWh m^−3^) and required membrane area (m^2^). (C) Pareto front optimization between specific energy consumption (kWh m^−3^) and average water flux (L m^−2^ h^−1^). (D) Water flux (L m^−2^ h^−1^) versus recovery rate (%). (E) Concentration polarization factor versus recovery rate (%). (F–H) (F) Specific energy consumption, (G) average water flux (L m^−2^ h^−1^), (H) required membrane area (m^2^) for optimized two-stage ROs and commercial one-stage RO. The optimized results for two-stage RO system are obtained with respect to various maximum concentration polarization factor (CPF_max_ = 1.20, 1.25, and 1.30) constraints. The conditions for all cases in the two-stage RO include a feed salinity of 35,000 ppm, an inlet flow rate of 300 m^3^ h^−1^, a total recovery rate of 50%, a pump efficiency of 85%, and an energy recovery efficiency of 95%.

Furthermore, the optimized membrane module with a V-shaped spacer (*β* = 7) is applied for designing two-stage UPM SWRO system (Figure 5A). The conditions for all cases in the two-stage SWRO include a feed salinity of 35,000 ppm, an inlet flow rate of 300 m^3^ h^−1^, a total recovery rate of 50%, a pump efficiency of 85%, and an energy recovery efficiency of 95%. The range of system design parameters for two-stage UPM SWRO is provided in Table S6. The optimized results for two-stage SWRO system are obtained with different maximum CPFs (CPF_max_ = 1.20, 1.25, and 1.30) constraints for various trade-off parameter values of *c*_m_ = 40, 100, 160, 220, 280, 340, and 400 $ m^−2^. The optimized results with a CPF_max_ = 1.25 constraint are preferred, as they result in a lower SEC under the same conditions for a required membrane area (Figure 5B) or an average water flux (Figure 5C) compared with those of CPF_max_ = 1.20, while effectively controlling membrane scaling and fouling.^49^ Varying the trade-off parameter *c*_m_ could yield different optimized solutions tailored to specific requirements. As *c*_m_ increases, the required membrane area decreases (Figure 5B) or average water production increases (Figure 5C), while energy consumption correspondingly rises. This is primarily due to the reduced number of membrane modules, higher crossflow velocity, and increased energy consumption from flow resistance. The ultrafast water flux at the inlet—exceeding 250 lmh in the case of CPF_max_ = 1.25 (Figure 5D)—is often associated with the highest concentration polarization (Figure 5E). Therefore, it is crucial to control the CPF at the inlet within a reasonable range (no more than 1.25). To balance the trade-off between SEC and the required membrane area (or average water flux), the optimized results with *c*_m_ = 220 $ m^−2^ (Figures 5F–5H) are recommended for further analysis.

Taking the case with a CPF_max_ = 1.25 constraint for example, the optimized SEC (1.78 kWh m^−3^) and required membrane area (1,583 m^2^) reduce 22% and 80%, respectively, compared with that of the commercial one-stage SWRO (2.30 kWh m^−3^ and 7,804 m^2^). Accordingly, the optimized average water flux (95 lmh) achieves a 391% increase compared with the estimated value from the commercial design (19 lmh). Detailed results for the two-stage UPM and commercial designs with various *c*_m_ values (40, 220, and 400 $ m^−2^) are presented in Tables S7–S9, respectively. Furthermore, we assess the repeatability of the system design. The simulated results demonstrated optimized performance and high reproducibility, with deviations of less than 3% in SEC and average permeate salinity (${\bar{w}}_{p}$), and below 7% in average water flux (${\bar{J}}_{W}$), between two independent calculations (Table S10).

**Figure 6:**
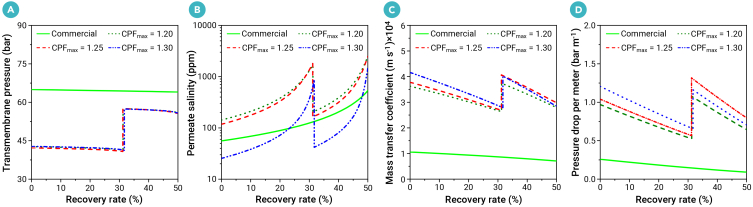
System performance analysis (A) Transmembrane pressure (bar), (B) permeate salinity (parts per million, ppm), (C) mass transfer coefficient (m s^−1^), and (D) pressure drop per meter (bar m^−1^) with respect to various recovery rate (%). *c*_m_ is 220 $ m^−2^ which is the trade-off parameter in the objective function (*F*_2_) to balance SEC (kWh m^−3^) and the required total membrane area (m^2^). The *F*_2_ is mathematically formulated as shown in Equation 5. The optimized results for two-stage RO system are obtained with respect to various maximum concentration polarization factor (CPF_max_ = 1.20, 1.25, and 1.30) constraints. The conditions for all cases in the two-stage RO include a feed salinity of 35,000 ppm, a total recovery rate of 50%, a pump efficiency of 85%, and an energy recovery efficiency of 95%.

The two-stage design operates at lower pressures—43.2 bar in the first stage and 58.5 bar in the second stage—compared with 66.0 bar in the commercial one-stage SWRO, resulting in a lower SEC (Figure 6A). Although the permeate salinity for the UPM systems will also increase compared with commercial membranes (Figure 6B), the average permeate salinity in all cases of this work remains below 500 ppm, meeting potable water standards.^52^ Under ultrafast water flux conditions, the mass transfer at the system inlet using the optimized membrane module is 3.57 times greater than that of the commercial design, accompanied by an approximately 4-fold increase in pressure drop per meter (Figures 6C and 6D). However, predictive models from a previous study^20^ indicate that achieving a 3.57-fold increase in mass transfer by merely increasing crossflow velocity in the commercial design would cause an impractical 97-fold rise in pressure drop. Hence, optimizing the synergistic relationship between velocity and concentration fields in the spacer-filled channel through feed spacer design offers a more effective strategy for enhancing mass transfer while balancing an acceptable pressure loss penalty, rather than simply increasing crossflow velocity. This further validates the field synergy theory proposed for enhancing heat transfer.^53^^,^^54^ The designed spacers will be further fabricated using advanced 3D printing technologies to assess their performance in hydrodynamics, mass transfer, and mechanical stability.

### Optimal design of UPM batch SWRO systems

**Figure 7:**
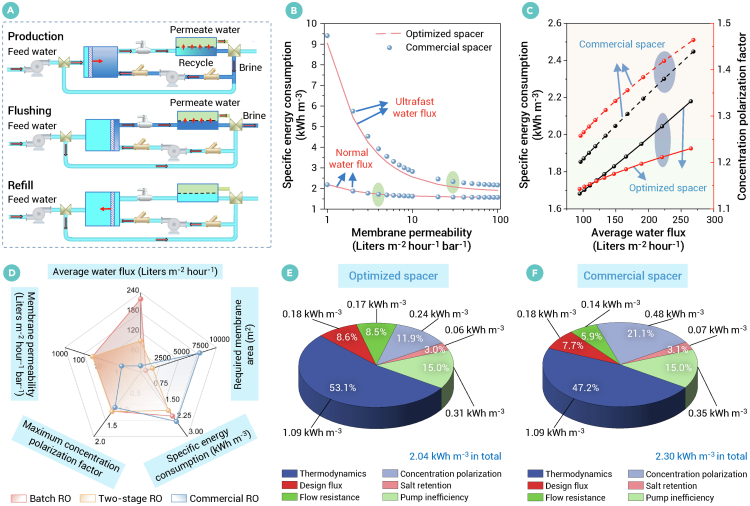
Optimal design of ultrapermeable batch reverse osmosis systems (A) Process flow diagram for batch RO. (B) SEC (kWh m^−3^) versus membrane permeability (L m^−2^ h^−1^ bar^−1^, lmh bar^−1^) for batch RO. The ultrafast average water flux: 221 lmh for the optimized V-shaped spacer and 224 lmh for the commercial spacer. Normal average water flux: 19 lmh. (C) SEC (kWh m^−3^) and concentration polarization factor versus average water flux (lmh) using an optimized V-shaped spacer and commercial spacer for batch RO. (D–F) (D) SEC (kWh m^−3^), average water flux (lmh), required membrane area (m^2^), maximum concentration polarization factor and membrane permeability (lmh bar^−1^) for optimized batch and two-stage ROs and commercial RO. Comparison of SEC breakdowns using (E) optimized V-shaped spacer and (F) commercial spacer for batch RO. The conditions for all cases in the batch RO include a feed salinity of 35,000 ppm, an inlet flow rate of 300 m^3^ h^−1^, a total recovery rate of 50%, and a pump efficiency of 85%.

Batch RO has attracted considerable attention due to its internal staging feature, which is divided into three stages: production, flushing, and refill (Figure 7A). A comparison of normalized SEC breakdowns for batch RO, including factors such as thermodynamics, design flux, flow resistance, concentration polarization, salt retention, and pump inefficiency, is presented in Table S11.

The SEC for batch RO is evaluated across various membrane permeabilities under both normal and ultrafast water flux conditions, using optimized and commercial spacers, respectively (Figure 7B). Under normal average water flux (19 lmh), SEC gradually decreases as membrane permeability increases from 1 to 4 lmh bar^−1^. When membrane permeability exceeds 4 lmh bar^−1^, further reductions in SEC become marginal. This is consistent with reported conclusions^24^ that the use of advanced membrane materials has a limited impact on further improving energy efficiency in RO and other desalination technologies. However, under ultrafast average water flux (>220 lmh), SEC continues to significantly decrease until membrane permeability exceeds 30 lmh bar^−1^ (Figure 7B). Thus, optimizing membrane permeability to around 30 lmh bar^−1^, coupled with ideal salt selectivity, is essential for enhancing energy efficiency in the context of ultrafast water flux. Optimization of the membrane module offers greater potential for reducing SEC under ultrafast water flux compared with normal water flux, while also mitigating CPF and the risks of membrane scaling and fouling. This is consistent with the conclusions reported in previous literature^20^ that the performance of the UPM is limited by the fluid mechanics and mass transfer of the membrane module, defining the upper bounds.

We further evaluate the potential through membrane permeability, membrane module design, and the use of batch RO, with the trade-off between SEC and average water flux shown in Figure 7C. For comparison, membrane permeability is fixed at 34.9 lmh bar^−1^, consistent with the optimized configuration in the two-stage design (*c*_m_ = 220 $ m^−2^). The optimized V-shaped spacer (*β* = 7) significantly reduces SEC compared with the commercial spacer, while effectively maintaining CPF below 1.25. If using the commercial spacer, the CPFs for all cases in batch RO are more than 1.25. When the ultrafast average water flux is increased to 224 lmh, the CPF increases to an impractical value of 1.42, further intensifying membrane fouling. Accordingly, the SEC is 2.30 kWh m^−3^, which does not provide energy-saving benefits compared with the commercial one-stage SWRO. Here, we propose an optimized design scheme incorporating an UPM, optimized membrane module, and batch RO (Figure 7D). The required membrane area (679 m^2^) and SEC (2.04 kWh m^−3^) are reduced by 91% and 11%, respectively. The average water flux reaches 221 lmh, which is 11.5 times higher than that of the commercial SWRO (19 lmh). The maximum CPF is 1.21, which falls within a reasonable range of less than 1.25.^49^ Comparison of SEC breakdowns (Figures 7E and 7F) reveals that higher concentration polarization is the primary factor contributing to the increase in SEC (from 2.04 to 2.30 kWh m^−3^) using the commercial spacer relative to that using the optimized spacer. If ultrahigh water flux is not a priority, the SEC can further reduce by 1.68 kWh m^−3^ with an average water flux of 95 lmh. It is twice that of the optimized two-stage design (47 lmh), with an equivalent SEC of 1.67 kWh m^−3^. This is mainly due to the uniform flux in batch RO, which allows operation at a higher average flux under the same maximum concentration polarization constraint, compared with the two-stage design. Detailed results using the optimized and commercial spacers in UPM batch SWRO desalination are presented in Tables S12 and S13, respectively.

## Discussion

In recent years, UPM materials have received widespread attention and extensive research. However, the mere discovery of new materials is insufficient for the realization of cost-effective water treatment technologies. The limitations in fluid dynamics and mass transfer of conventional membrane module may define the upper bounds of membrane performance.^20^ It is imperative to design and develop advanced membrane materials within the context of integrated unit processes and the entire water treatment system.^55^ By combining process system optimization models with techno-economic analysis and life cycle assessments, it becomes possible to redefine the theoretical upper limits of membrane performance improvements while simultaneously exploring the optimal balance between capital and operational costs.^55^ Herein, we propose a multiscale optimization framework coupling the optimal selection of membrane permeability, membrane module optimization and system design (with two-stage and batch configurations) in SWRO desalination. A Bayesian-driven pattern search approach is developed for feed spacer design.

This work presents a transformative advance in membrane desalination technology by fundamentally redefining the long-standing trade-off between energy efficiency and water production efficiency (or average water flux). Through the synergistic integration of bio-inspired UPM module with state-of-the-art batch RO, we demonstrate unprecedented performance—achieving a SEC of just 1.68 kWh m^−3^ while delivering an average water flux of 95 lmh. This represents a 33%–58% reduction in energy demand and a 5-fold improvement in water flux compared with modern plants for seawater RO desalination (feed salinity of 35,000 ppm, recovery rate of 50%). The concentration polarization is maintained within a reasonable range (no more than 1.25), reducing the risks of membrane scaling and fouling. The desalination energy efficiency, achieved through UPMs, optimized module design, and batch RO, shows significant improvement under ultrafast average water flux. However, when membrane permeability exceeds 30 lmh bar^−1^, additional reductions have minimal impact, unless future advancements yield membrane modules or processes with superior fluid dynamics and mass transfer characteristics relative to the present work. This finding is crucial for guiding the future direction of membrane material development and improving energy efficiency of desalination technologies. These breakthroughs redefine desalination technology, demonstrating how engineered UPMs can simultaneously boost energy efficiency and water production efficiency. Our findings establish design principles that enable next-generation desalination plants and open new possibilities for zero-liquid-discharge systems^5^ and other advanced water treatment applications.

Our prior work^56^ introduced a hybrid model for RO desalination, integrating 3D CFD with a one-dimensional system-scale approach. Simulations using this framework yielded transmembrane pressure predictions within 5% of industrial data and closely matched the total flow rate, as benchmarked against operational data^57^ from the Chino Desalter I facility, California. Nevertheless, the multiscale design scheme, e.g., structural integrity for the designed innovative membrane module (or feed spacer) under high-pressure operation still requires experimental validation in future work. The energy recovery device operates within the two-stage system at relatively constant pressure. Progressive membrane fouling during operation reduces water permeability, necessitating elevated operational pressure to maintain constant flux. This pressure increase may perturb energy recovery device efficiency; however, the effects of pressure fluctuations lie beyond this work’s scope and warrant future investigation. Furthermore, the assumption of identical residence time distributions across modules is limited by potential fouling-induced variability. The implementation of the optimized system faces practical challenges: (1) the scalable production of UPMs that balance high salt rejection and mechanical strength with low cost; (2) the scalable manufacturing of optimized feed spacers, despite the promise of low-cost methods such as 3D printing; and (3) the development of a closed-loop control system to maintain constant permeate flux via real-time pressure adjustments.

## Resource availability

### Materials availability

This study did not generate new unique materials/reagents.

### Data and code availability

Data and code are available from the corresponding author upon reasonable request.

## Funding and acknowledgments

Y.H. acknowledges support provided by the 10.13039/501100015956Key Area Research and Development Program of Guangdong Province, China (2021B0101190003). J.L. thanks support provided by the Opening Project of Guangdong Province Key Laboratory of Computational Science at the Sun Yat-sen University (2024014). J.W. thanks support provided by the Suzhou Planning Project of Science and Technology (2023ss03) and the Key Laboratory of General Artificial Intelligence and Large Models in Provincial Universities, Soochow University.

## Author contributions

J.L. and Y.H. designed the research. J.L. and X.L. provided the data, models and computational methods, and performed the research. J.L., X.L., J.W., and Y.H. analyzed the data and wrote the paper.

## Declaration of interests

The authors declare no competing interests.

## Supplemental information

Document S1. Figures S1–S3, Tables S1–S13, and Notes S1–S3

Document S2. Article plus supplemental information
