# Supplementary material for: Intelligent design breaks the trade-off between energy efficiency and water flux in ultrafast seawater desalination
Source: Innovation (Camb). 2026 Jan 7;7(5):101262. doi: 10.1016/j.xinn.2026.101262 (PMC13147988; doi:10.1016/j.xinn.2026.101262)
Supplement: Document S1. Figures S1–S3, Tables S1–S13, and Notes S1–S3 [file mmc1.pdf]

The Innovation, Volume 7

Supplemental Information

Intelligent design breaks the trade-off between energy efficiency and water flux in ultrafast seawater desalination

Jiu Luo, Xing Liu, Jin Wang, and Yi Heng

Supplementary Information for

Intelligent design breaks the trade-off between energy efficiency and water flux in ultrafast seawater desalination

Jiu Luo[1, 2], Xing Liu[1], Jin Wang[1, 2], Yi Heng[3*]

[1] School of Future Science and Engineering, Soochow University, Suzhou, 215222, China

[2] Key Laboratory of General Artificial Intelligence and Large Models in Provincial Universities, Soochow University, Suzhou, 215222, China

[3] School of Computer Science and Engineering, Sun Yat-sen University, Guangzhou, 510006, China

* Correspondence: hengyi@mail.sysu.edu.cn (Y. H.)

This PDF file includes:

Supplementary notes S1-S3
Figures S1-S3
Tables S1-S13
Supplementary references

Supplementary Note S1

1.1. Bayesian algorithm

During the global exploration phase, traditional intelligent optimization techniques, such as particle swarm optimization and genetic algorithm, typically require a large number of iterative evaluations to progressively approximate the global optimum. These methods lack an efficient mechanism to prioritize the exploration of potentially optimal regions, relying instead on randomness and natural selection, which often leads to redundant function evaluations. Given the black-box nature of the problem, Bayesian optimization presents a more suitable alternative. By leveraging probabilistic models, Bayesian optimization systematically approximates the objective function, enabling more informed and efficient exploration.

In this work, we employ the widely used Gaussian process model, defined as $F(x) \sim GP(\mu(x), \sigma^2(x))$, where $\mu(x)$ represents the predicted mean and $\sigma(x)$ quantifies the prediction uncertainty. Beyond estimating the objective value at each point, the Gaussian process also provides an uncertainty measure, enabling Bayesian optimization to strategically select sampling points that maximize potential improvement while reducing uncertainty. For complex and computationally expensive objective functions, this probabilistic modeling approach allows Bayesian optimization to efficiently identify the optimal solution while maintaining relatively low computational costs.

Among common acquisition functions—such as Probability of Improvement (PI)[1], Expected Improvement (EI)[2], and Lower Confidence Bound (LCB)[3]—EI is often preferred, and is expressed as:

$$EI(x) = (\mu(x) - f(x+))\Phi\left(\frac{(\mu(x) - f(x+))}{\sigma(x)}\right) + \sigma(x)\varphi\left(\frac{(\mu(x) - f(x+))}{\sigma(x)}\right) \quad (1)$$

where $\mu(x)$ is the mean of the Gaussian process at point x, $f(x+)$ is the current known optimal function value, $\Phi(\bullet)$ is the cumulative distribution function of the standard normal distribution, $\sigma(x)$ is the standard deviation of the Gaussian process at point x, and $\varphi(\bullet)$ is the probability density function of the standard normal distribution. However, when tackling the complex PDE problems of this work, the expected improvement (EI) criterion tends to become trapped in local optima. To overcome this limitation, the EI-Plus (EIP) acquisition function[4] is adopted. EIP incorporates a self-correcting mechanism to prevent over-exploitation, which is mathematically expressed as:

$$\sigma_Q{}^2(x) = \sigma_F{}^2(x) + \sigma^2,\ for\ \sigma_F(x) < t_\sigma \sigma, \quad (2)$$

where $\sigma_F(x)$ denotes the standard deviation of the posterior objective function at x while σ represents the posterior standard deviation of the additive noise. The adjusted standard deviation is given by $\sigma_Q(x)$, and t_σ (set to 0.8 in this work) defines the adjustable exploration–exploitation trade-off ratio. If the condition of $\sigma_F(x) < t_\sigma \sigma$ is not satisfied, the algorithm adaptively increases the variance between observation points by modifying the kernel function.[4]

1.2. Pattern search algorithm

In the local exploitation phase, gradient descent, Newton's method, and quasi-Newton methods are among the most widely used local optimization techniques due to their broad applicability and favorable convergence properties. However, these methods require the

objective function to be continuous and differentiable, making them heavily reliant on derivative information—an impractical assumption for complex PDE problems. In contrast, the pattern search algorithm provides a derivative-free alternative that is computationally efficient, straightforward to implement, and inherently more robust, making it well-suited for addressing the optimal design problem of membrane module in this work.

The pattern search algorithm determines search directions using pattern vectors. In the context of the optimal design of membrane module in this work, where the decision variables have six dimensions, 12 fundamental search directions are defined as a positively definite extension set $[d_1, d_2, \cdots, d_{11}, d_{12}]$ where $d_1 = (1, 0, ..., 0)$, $d_2 = (-1, 0, ..., 0)$ ..., $d_{12} = (0, ..., 0, -1)$. This formulation ensures comprehensive exploration across the search space. The algorithm employs pattern directions and the current grid size to systematically search around the incumbent solution. Here, the grid represents a conceptual mesh of candidate points from which the algorithm selects evaluation points. To enhance local refinement, we initialize the pattern search algorithm at the optimized solution identified through Bayesian optimization and adopt a smaller initial grid size, facilitating more precise local exploration.

If the search step fails to identify an improved solution, the algorithm proceeds to a polling step. Similar to the exploration phase, this step systematically evaluates alternative positions; however, instead of relying on a subset of the previously defined positive definite extension set, it sequentially traverses each direction. If a polling point yields a better objective value than the current position, it is adopted as the new incumbent solution, the grid size is increased, and the algorithm resumes the search step. Accordingly, if the polling

step fails to identify an improved solution, the current position remains unchanged, the grid size is reduced, and the search step is reinitiated. The algorithm terminates upon meeting the predefined convergence criteria, which include a minimum grid size tolerance of 10^{-10}, stabilization of the objective function value within a threshold of 10^{-7} over five consecutive iterations, and a maximum iteration limit. These criteria ensure high solution accuracy while enhancing the adaptability of the algorithm.

Supplementary Note S2

2.1. 3D high-fidelity multi-physics simulations

In spiral-wound membrane systems, each module consists of over a million feed spacer cells, making full-scale 3D multi-physics simulations computationally infeasible. However, prior research indicates that flow and mass transfer reach a periodic fully-developed state within just a few spacer cells[5]. This allows for a substantial reduction in computational complexity by focusing on a localized domain while still capturing the essential hydrodynamic and transport characteristics. To systematically evaluate system performance, the mass transfer coefficient and axial pressure drop per unit length are assessed at different positions along the feed direction by modulating the inlet feed velocity. In this study, high-fidelity 3D multi-physics models is employed to resolve fluid flow and mass transport within a computational domain consisting of five unit cells (Figure S1), providing an efficient yet accurate framework for analyzing local transport phenomena for water production process (3) and flushing process (4) during the flushing process of batch reverse osmosis, as below:

$$
\begin{cases}
\rho(\mathbf{u}\cdot\nabla)\mathbf{u} = \nabla\cdot[-P\mathbf{I} + \mu(\nabla\mathbf{u} + (\nabla\mathbf{u})^{\mathrm{T}})], & \text{in } \Omega, \\
\nabla\cdot(\rho\mathbf{u}) = 0, & \text{in } \Omega, \\
\nabla\cdot(D_s\nabla c) = \mathbf{u}\cdot\nabla c, & \text{in } \Omega, \\
\mathbf{u}_{\mathrm{in}} = \mathbf{u}_{\mathrm{out}}, (\nabla\mathbf{u})_{\mathrm{in}} = (\nabla\mathbf{u})_{\mathrm{out}}, \bar{u}_{\mathrm{ave,\,in}} = U_0, & \text{on } (\boldsymbol{\Gamma}_I \cup \boldsymbol{\Gamma}_O), \\
(\nabla P)_{\mathrm{in}} = (\nabla P)_{\mathrm{out}}, P_{\mathrm{out}} = 0, & \text{on } (\boldsymbol{\Gamma}_I \cup \boldsymbol{\Gamma}_O), \\
\mathbf{u}_L = \mathbf{u}_{\mathrm{R}}, (\nabla\mathbf{u})_L = (\nabla\mathbf{u})_R, & \text{on } (\boldsymbol{\Gamma}_L \cup \boldsymbol{\Gamma}_R), \\
P_L = P_{\mathrm{R}}, (\nabla P)_L = (\nabla P)_R, & \text{on } (\boldsymbol{\Gamma}_L \cup \boldsymbol{\Gamma}_R), \\
\mathbf{u} = \mathbf{0}, & \text{on } (\boldsymbol{\Gamma}_U \cup \boldsymbol{\Gamma}_B \cup \boldsymbol{\Gamma}_S), \\
c_{\mathrm{in}} = c_0, (-\mathbf{n}\cdot D_{\mathrm{s}}\nabla c)_{\mathrm{out}} = 0, & \text{on } (\boldsymbol{\Gamma}_I \cup \boldsymbol{\Gamma}_O), \\
c_L = c_R, (\nabla c)_L = (\nabla c)_R, & \text{on } (\boldsymbol{\Gamma}_L \cup \boldsymbol{\Gamma}_R), \\
\mathbf{n}\cdot(-D_{\mathrm{s}}\nabla c + c\mathbf{u}) = 0, & \text{on } (\boldsymbol{\Gamma}_U \cup \boldsymbol{\Gamma}_B \cup \boldsymbol{\Gamma}_S),
\end{cases}
\tag{3}
$$

$$
\begin{cases}
\rho(\mathbf{u}\cdot\nabla)\mathbf{u} = \nabla\cdot[-P\mathbf{I} + \mu(\nabla\mathbf{u} + (\nabla\mathbf{u})^{\mathrm{T}})], & \text{in } \Omega, \\
\nabla\cdot(\rho\mathbf{u}) = 0, & \text{in } \Omega, \\
\dfrac{\partial c}{\partial t} = \nabla\cdot(D_s\nabla c) - \mathbf{u}\cdot\nabla c, & \text{in } \Omega\times[0, t_{\mathrm{f}}], \\
c\big|_{t=0} = 2c_0, & \text{on } \Omega, \\
\mathbf{u}_{\mathrm{in}} = \mathbf{u}_{\mathrm{out}}, (\nabla\mathbf{u})_{\mathrm{in}} = (\nabla\mathbf{u})_{\mathrm{out}}, \bar{u}_{\mathrm{ave,\,in}} = U_0, & \text{on } (\boldsymbol{\Gamma}_I \cup \boldsymbol{\Gamma}_O), \\
(\nabla P)_{\mathrm{in}} = (\nabla P)_{\mathrm{out}}, P_{\mathrm{out}} = 0, & \text{on } (\boldsymbol{\Gamma}_I \cup \boldsymbol{\Gamma}_O), \\
\mathbf{u}_L = \mathbf{u}_R, (\nabla\mathbf{u})_L = (\nabla\mathbf{u})_R, & \text{on } (\boldsymbol{\Gamma}_L \cup \boldsymbol{\Gamma}_R), \\
P_L = P_R, (\nabla P)_L = (\nabla P)_R, & \text{on } (\boldsymbol{\Gamma}_L \cup \boldsymbol{\Gamma}_R), \\
\mathbf{u} = \mathbf{0}, & \text{on } (\boldsymbol{\Gamma}_U \cup \boldsymbol{\Gamma}_B \cup \boldsymbol{\Gamma}_S), \\
c_{\mathrm{in}} = c_0, (-\mathbf{n}\cdot D_{\mathrm{s}}\nabla c)_{\mathrm{out}} = 0, & \text{on } (\boldsymbol{\Gamma}_I \cup \Gamma_O)\times(0, t_{\mathrm{f}}), \\
c_L = c_R, (\nabla c)_L = (\nabla c)_R, & \text{on } (\boldsymbol{\Gamma}_L \cup \boldsymbol{\Gamma}_R)\times(0, t_{\mathrm{f}}), \\
\mathbf{n}\cdot(-D_{\mathrm{s}}\nabla c + c\mathbf{u}) = 0, & \text{on } (\boldsymbol{\Gamma}_U \cup \boldsymbol{\Gamma}_B \cup \boldsymbol{\Gamma}_S)\times(0, t_{\mathrm{f}}).
\end{cases}
\tag{4}
$$

The computational domain (Ω) is defined within a narrow spacer-filled channel, as shown in Figure S1, where the boundaries $\boldsymbol{\Gamma}_I$, $\boldsymbol{\Gamma}_O$, $\boldsymbol{\Gamma}_L$, $\boldsymbol{\Gamma}_R$, $\boldsymbol{\Gamma}_U$, $\boldsymbol{\Gamma}_B$ and $\boldsymbol{\Gamma}_S$ are depicted. The total simulation time in Eq. (4) is denoted as t_{f}. The fluid properties, including density (ρ), viscosity (μ), and salt diffusivity (D_{s}), are assumed to remain constant throughout the

simulation. The key variables governing the system include the velocity vector (**u**), hydraulic pressure (P), and molar concentration (c).

2.2. Hydrodynamics and transport characteristics

The Darcy friction factor is computed based on flow characteristics, including cross velocity ($\bar{u}$), axial pressure drop (ΔP_{c}), and spacer geometry, as below[6]

$$f = \frac{2D_{\mathrm{H}}\Delta P_{\mathrm{c}}}{\rho \bar{u}^2 L}, \tag{5}$$

where D_{H} and L represent the hydraulic diameter and the computational domain's channel length, respectively (Figure S1). $\Delta P_c / L$ and D_{H} are estimated by

$$-\frac{\Delta P_c}{L} = -\frac{\bar{P}_{\mathrm{out}} - \bar{P}_{\mathrm{in}}}{L}, \tag{6}$$

and

$$D_H = \frac{4\varepsilon}{2/H + (1-\varepsilon)S_{\mathrm{sp}}/V_{\mathrm{sp}}}, \tag{7}$$

respectively where S_{sp} and V_{sp} denote surface area and volume of spacers in feed channel. H is channel height. Channel porosity ε is defined as

$$\varepsilon = \frac{V_{\mathrm{tot}} - V_{\mathrm{sp}}}{V_{\mathrm{tot}}}, \tag{8}$$

which V_{tot} is the volume of rectangular prism (Figure S1). The Sherwood number (Sh), determined as a function of cell-average mass transfer coefficient ($\bar{k}_{\mathrm{m}}$) on membrane walls can be expressed as

$$Sh = \bar{k}_{\mathrm{m}} \frac{D_{\mathrm{H}}}{D_{\mathrm{s}}}. \tag{9}$$

$\bar{k}_{\mathrm{m}}$ is defined by

$$\bar{k}_{\mathrm{m}} = \frac{\int_0^L \mathrm{d}y \int_0^W \left(\frac{-D_{\mathrm{s}}}{c_{\mathrm{r}} - c_{\mathrm{w}}} \cdot \frac{\partial c}{\partial z}\right) \mathrm{d}x}{\int_0^L \mathrm{d}y \int_0^W \mathrm{d}x}. \tag{10}$$

Here, c_{r} and c_{w} represent the solute concentration in the bulk retentate and at the membrane surface, respectively.

Using CFD simulations across a wide range of Reynolds numbers (*Re* = 50, 62.5, 75 … 1,000), we derive empirical correlations for the Sherwood number and Darcy friction factor. These correlations characterize mass transfer and flow resistance within the spacer-filled channel and are expressed as follows:

$$Sh_{\mathrm{imp}} = kRe^t, \tag{11}$$

$$f = \sum_{i=1}^{8} (a_i Re^i + b_0), \tag{12}$$

The Reynolds number is determined as a function of crossflow velocity, incorporating fluid properties and channel geometry to characterize flow behavior within the spacer-filled domain, as following

$$Re = \frac{D_{\mathrm{H}} \bar{u} \rho}{\mu}. \tag{13}$$

The correlations (11) and (12) establish a quantitative framework for analyzing mass transfer and flow resistance, facilitating the optimal system design.

2.3. Flushing efficacy for batch RO

Batch reverse osmosis operates in three phases: water production, flushing and refill. During the flushing process, incomplete flushing causes salt retention, which impairs

performance by increasing salt concentration, raising peak pressure, and elevating power consumption. The flushing efficacy (f) is estimated using the following formula[7]

$$f\left(\theta\right)=\int_0^{\theta}\left(1-F\left(\theta\right)\right)\mathrm{d}\theta. \tag{14}$$

The cumulative function of residence time distribution (CRTD), $F\left(t\right)$ is evaluated from[7]

$$F\left(t\right)=\frac{\iint_{\mathrm{out}}c_{\mathrm{out}}(t)u\mathrm{d}y\mathrm{dz}}{c_{\mathrm{in}}\iint_{\mathrm{in}}u\mathrm{d}y\mathrm{dz}}, \tag{15}$$

The residence time distribution (RTD), $E\left(t\right)$ is determined by[7]

$$E\left(t\right)=\frac{\mathrm{d}F\left(t\right)}{\mathrm{d}t}, \tag{16}$$

Here, c_{in} represents the constant inlet concentration, while outlet concentration of $c_{\mathrm{out}}\left(t\right)$ varies over time. To evaluate the influence of design parameters on RTD, CRTD, and flushing efficiency, the dimensionless time is defined as $\theta = t/\tau$. The space time (τ) is determined using the following equation

$$\tau=\frac{\iiint_{\Omega}\mathrm{d}x\mathrm{d}y\mathrm{dz}}{\iint_{in}u\mathrm{d}y\mathrm{dz}}, \tag{17}$$

which u denote the velocity alone the feed direction (Figure S1). In a full-scale RO system, where multiple RO elements are arranged in series, the overall RTD is determined through a convolution operation[7], expressed as follows:

$$\begin{aligned}
c_{\mathrm{out}}^{1}\left(t\right)&=conv\left(c_{\mathrm{in}}^{1}\left(t\right),E_{\mathrm{in}}^{1}\left(t\right)\right),\\
c_{\mathrm{out}}^{2}\left(t\right)&=conv\left(c_{\mathrm{out}}^{1}\left(t\right),E_{\mathrm{out}}^{1}\left(t\right)\right),\\
&\vdots\\
c_{\mathrm{out}}^{n}\left(t\right)&=conv\left(c_{\mathrm{out}}^{n\text{-}1}\left(t\right),E_{\mathrm{out}}^{n\text{-}1}\left(t\right)\right),
\end{aligned} \tag{18}$$

Here, $c_{\text{out}}^{n\text{-}1}$ and $E_{\text{out}}^{n\text{-}1}$ ($n \in N^{+}$) represent the outlet concentration and RTD, respectively, for the (n-1)th membrane module within a pressure vessel. Similarly, c_{in}^{1} and E_{in}^{1} denote the inlet concentration and RTD for the first membrane module. Since all membrane elements in the RO system share identical geometric structures, they exhibit the same RTD, meaning $E_{\text{in}}^{1}(t) = E_{\text{out}}^{1}(t) = \ldots = E_{\text{out}}^{n\text{-}1}(t) = E(t)$. $E(t)$ is the RTD through CFD simulations in this work.

Supplementary Note S3

3.1. System modeling at industry-scale

At the industrial scale, the RO process can be mathematically modeled using one-dimensional (1D) differential-algebraic equations (DAEs).[8] The governing equations for k^{th} stage of the RO process are formulated as follows

$$\begin{cases} \dfrac{\mathrm{d}Q}{\mathrm{d}X} = -J_{\mathrm{W}} \cdot A_{k} & X = k-1, Q = Q_{k-1}, \\ \dfrac{\mathrm{d}(\Delta P)}{\mathrm{d}X} = -\dfrac{\rho \bar{u}^{2} f}{2D_{H}} \cdot \left(n_{\text{mem}, k} \cdot l_{y}\right) & X = k-1, \Delta P = \Delta P_{k-1}, \\ \dfrac{\mathrm{d}w_{\mathrm{b}}}{\mathrm{d}X} = J_{\mathrm{W}} \cdot \dfrac{A_{k}}{Q}\left(w_{\mathrm{b}} - w_{\mathrm{p}}\right) & X = k-1, w_{\mathrm{b}} = w_{\mathrm{b},\, k-1}, \\ J_{\mathrm{W}} = L_{\mathrm{P}}\left(\Delta P - \sigma \cdot \varphi R_{\text{salt}} w_{\mathrm{w}}\right) & \end{cases} \tag{19}$$

In Equation (19), the primary variables to be determined include the flow rate (Q), transmembrane pressure (ΔP), water flux (J_{w}), and salinity concentrations at different points: within the retentate bulk (w_{b}), permeate bulk (w_{p}), and along the membrane surface (w_{w}). These parameters evolve along the dimensionless axial coordinate ($X \in [k-1, k]$),

which represents the normalized position along the membrane length. The expression for Q is given by:

$$Q = N_{\text{pv},k} n_{\text{sp}} l_x H \varepsilon \bar{u}, \tag{20}$$

In this formulation, $N_{\text{pv},k}$ refers to the number of pressure vessels in a given stage, while n_{sp} indicates the number of spacer sheets per module. The parameters l_x, H and ε represent the membrane sheet length perpendicular to the feed flow direction (*x* direction, Figure S1), the height of each feed channel, and the porosity of the feed channel, respectively. The total membrane surface area (A_k) for a given stage, k^{th}, is determined using the following equation

$$A_k = N_{\text{pv},k} \cdot n_{\text{mem},k} \cdot A_0 \cdot l_y / l_{y,0}, \tag{21}$$

In this model, $n_{\text{mem},k}$ represents the number of modules in a given stage of kth. The parameters l_y and $l_{y,0}$ refer to the membrane sheet lengths along the feed flow direction (*y*-direction, in Figure S1) for the optimized module (0.5 m) and the commercial module (1 m), respectively. The membrane area of a commercial module is denoted as A_0. The reflection coefficient (σ) and osmotic pressure coefficient (φ) are considered constants. Additionally, R_{salt} represents the intrinsic rejection efficiency of the membrane. The values of w_{p} and w_{w} can be calculated using the following equations[8]

$$w_{\text{p}} = w_{\text{b}} / \left[\exp\left(\ln \frac{J_{\text{W}}}{B} - \frac{J_{\text{W}}}{\bar{k}_{\text{m, per}}} \right) + 1 \right], \tag{22}$$

and

$$w_{\mathrm{w}} = \frac{w_{\mathrm{p}}}{1 - R_{\mathrm{salt}}}, \tag{23}$$

respectively.

The derivation of Eq. (22) is provided in our previous work.[8] The term $\bar{k}_{\mathrm{m,\,per}}$ represents the cell-averaged mass transfer coefficient on the permeable wall, which can be estimated using Eqs. (24) and (25)[9], as shown below

$$\bar{k}_{\mathrm{m,\,per}} = \bar{k}_{\mathrm{m}} \left[\psi + \left(1 + 0.26\psi^{1.4} \right)^{-1.7} \right], \quad (\psi < 20) \tag{24}$$

$$\psi = \frac{J_{\mathrm{W}}}{\bar{k}_{\mathrm{m}}}. \tag{25}$$

Here, $\bar{k}_{\mathrm{m}}$ for a given system can can be determined using Eqs. (10), (11), and (13), based on the CFD simulations. By combining Eqs. (19) to (23), the system-level variables (Q, ΔP, J_{W}, w_{b}, w_{p}, w_{w}) can be solved.

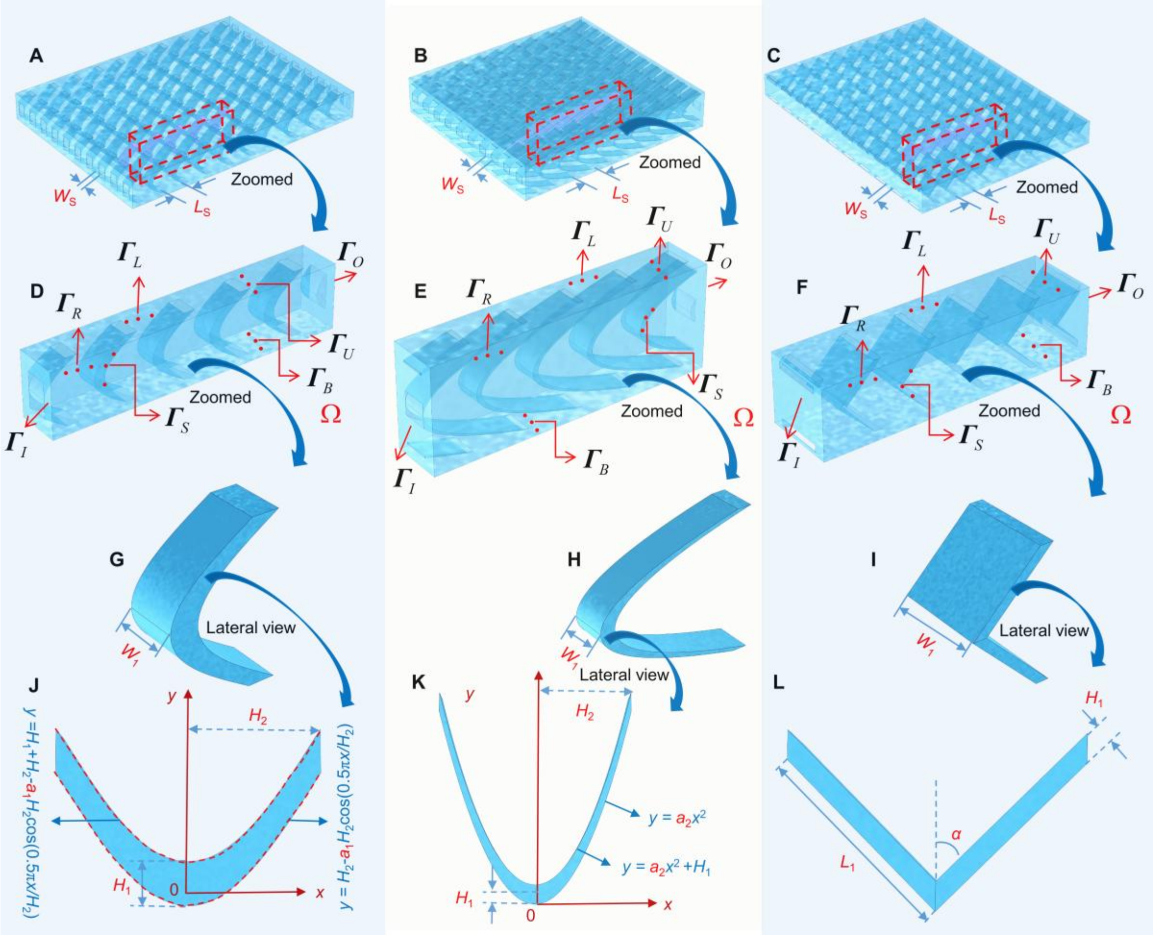

Figure S1. Establishment of parametric geometric models. Small segments of spacer sheets designed for **A**, a cosine shape, **B**, a parabolic shape and **C**, a V-shape. Computational domains of three-dimensional multi-physics model for **D**, a cosine shape, **E**, a parabolic shape and **F**, a V-shape. Spacer cells corresponding to **G**, a cosine shape, **H**, a parabolic shape and **I**, a V-shape. Lateral views of the spacer cells for **J**, a cosine shape, **K**, a parabolic shape and **L**, a V-shape. The cosine-shaped spacers-filled channel is characterized by distance parameters (L_S, W_S), size parameters (H_1, H_2, W_1), and a shape parameter (a_1). The parabolic-shaped spacers-filled channel is defined by distance parameters (L_S, W_S), size parameters (H_1, H_2, W_1), and a shape parameter (a_2). Specifically, the V-shaped spacers-filled channel is defined by distance parameters (L_S, W_S), size parameters (L_1, H_1, W_1), and a shape parameter (α).

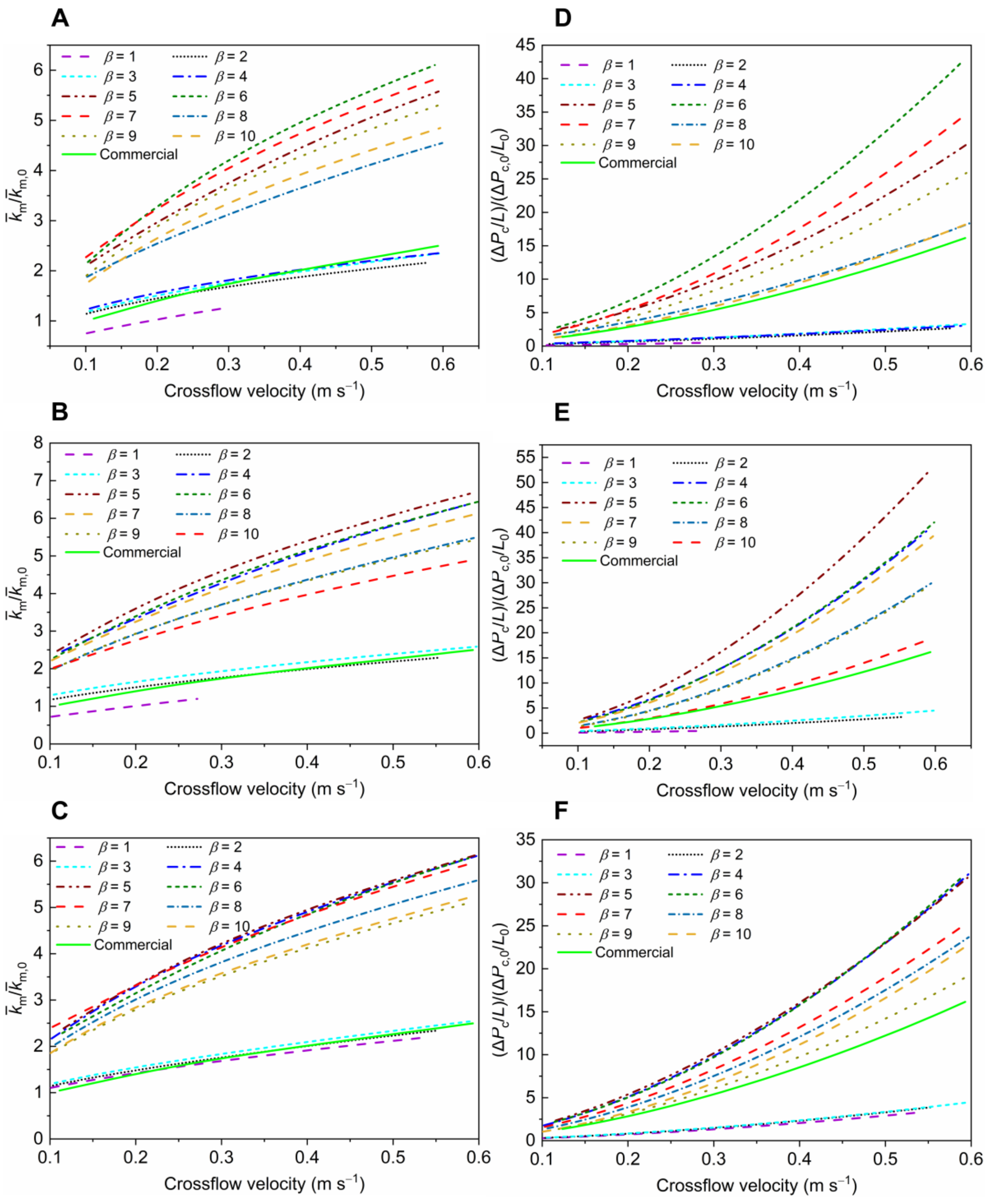

Figure S2. Mass transfer and pressure drop of optimized and commercial spacers. Ratios of cell-averaged mass transfer coefficients ($\bar{k}_m$) for optimized spacers with **A**, V-shape, **B**, cosine-shape, and **C**, parabola-shape, compared to the commercial spacer ($\bar{k}_{m,0}$). Ratios of pressure drops per meter ($\Delta P_c / L$) for optimized spacers with **D**, V-shape, **E**, cosine-shape, and **F**, parabola-shape, relative to the commercial spacer ($\Delta P_{c,0} / L_0$).

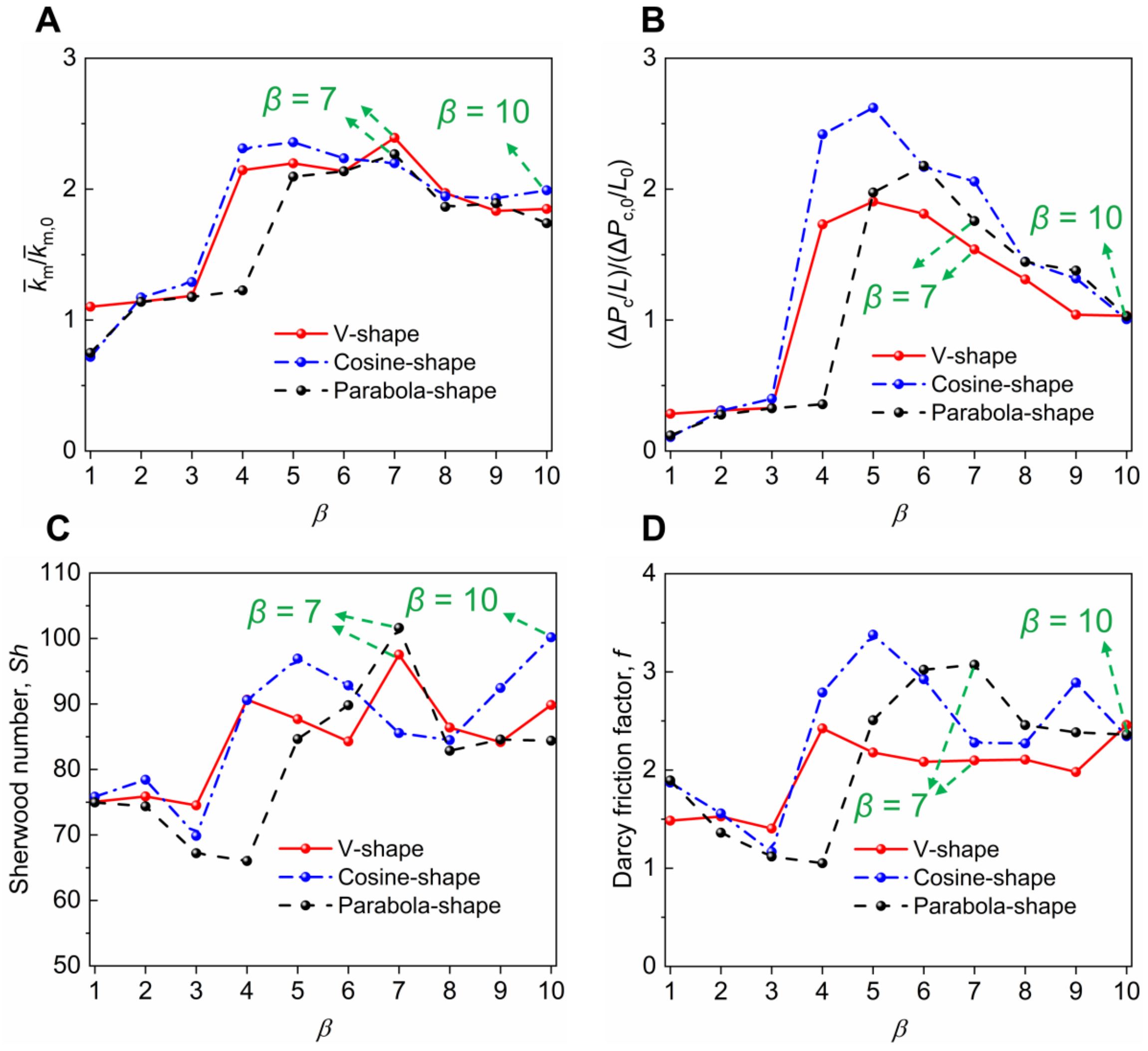

Figure S3. Identification of optimal tradeoff parameters. A, Ratios of cell-averaged mass transfer coefficients for the optimized spacer ($\bar{k}_m$) versus the commercial spacer ($\bar{k}_{m,0}$) as a function of β. **B,** Ratios of pressure drops per meter for the optimized spacer ($\Delta P_c / L$) versus the commercial spacer ($\Delta P_{c,0} / L_0$) as a function of β. **C**, Sherwood number for the optimized spacers as a function of β. **D**, Darcy friction factor for the optimized spacers as a function of β. β represents the tradeoff parameter in the objective function (F_1) to balance pressure drop and mass transfer. A detailed mathematical formulation of F_1 is provided in the *MATERIALS AND METHODS* section. The cross velocity is set to 0.1 m s^{-1} in **A** and **B**, while the Reynolds number is fixed at 100 in **C** and **D**.

Table S1. The range of geometric design parameters. The design parameters are represented as $\boldsymbol{\beta}_1 = [L_S, W_S, a_1, W_1, H_1, H_2]$ for cosine-shape, $\boldsymbol{\beta}_1 = [L_S, W_S, a_2, W_1, H_1, H_2]$ for parabolic-shaped, and $\boldsymbol{\beta}_1 = [L_S, W_S, \alpha, W_1, H_1, L_1]$ for V-shaped membrane modules, respectively, as shown in Figure S1.

**Range of design parameters**		**Value**					
		L_S (μm)	W_S (μm)	a_1 (m) or a_2 (m^{-1}) or α (°)	W_1 (μm)	H_1 (μm)	H_2 (μm) or L_1 (μm)
Cosine-shape	Minimum	400	200	0.25	100	10	300
	Maximum	2,000	1,000	1.25	1,000	100	1,000
Parabola-shape	Minimum	400	200	800	100	10	300
	Maximum	2,000	1,000	3,000	1,000	100	1,000
V-shape	Minimum	400	200	25	100	10	300
	Maximum	2,000	1,000	70	1,000	100	1,000

Table S2. Optimized outcomes for cosine-, parabolic-, and V-shape spacers. Geometric design parameters and computational domain size are investigated in relation to various trade-off parameter values of *β*. The design parameters are represented as $\boldsymbol{\beta}_1 = [L_S, W_S, a_1, W_1, H_1, H_2]$ for cosine-shape, $\boldsymbol{\beta}_1 = [L_S, W_S, a_2, W_1, H_1, H_2]$ for parabolic-shaped, and $\boldsymbol{\beta}_1 = [L_S, W_S, \alpha, W_1, H_1, L_1]$ for V-shaped membrane modules, respectively, as shown in Figure S1. The computational domain size consists of channel length (*L*), width (*W*) and height (*H*).

**Cases**		**Optimized results**								
		Geometric design parameters ($\boldsymbol{\beta}_1$)						Computational domain size		
		L_S (μm)	W_S (μm)	a_1 (m) or a_2 (m^{-1}) or α (°)	W_1 (μm)	H_1 (μm)	H_2 (μm) or L_1 (μm)	L (mm)	W (mm)	H (mm)
Cosine-shape	$\beta = 1$	1,966	964	1.24	102	14	993	9.90	1.07	1.99
	$\beta = 2$	411	1,000	1.05	102	17	597	2.14	1.10	1.19
	$\beta = 3$	403	990	1.07	101	28	399	2.15	1.09	0.80
	$\beta = 4$	409	252	1.09	132	14	340	2.11	0.38	0.68
	$\beta = 5$	501	274	1.09	217	26	401	2.63	0.49	0.80
	$\beta = 6$	563	274	1.16	190	13	357	2.88	0.46	0.71
	$\beta = 7$	472	312	1.20	179	17	304	2.44	0.49	0.61
	$\beta = 8$	583	408	1.18	223	13	372	2.98	0.63	0.74
	$\beta = 9$	887	468	1.22	359	12	491	4.49	0.83	0.98
	$\beta = 10$	657	449	1.10	212	38	510	3.48	0.66	1.02
Parabola-shape	$\beta = 1$	1,816	994	1,511	105	68	984	9.42	1.10	1.97
	$\beta = 2$	411	972	1,023	111	73	583	2.42	1.08	1.17
	$\beta = 3$	478	947	2,304	110	66	445	2.72	1.06	0.89
	$\beta = 4$	405	989	1,631	119	53	393	2.29	1.11	0.79
	$\beta = 5$	414	288	2,924	133	33	365	2.24	0.42	0.73
	$\beta = 6$	558	315	2,880	240	36	369	2.97	0.55	0.74
	$\beta = 7$	644	378	2,567	261	22	444	3.33	0.64	0.89
	$\beta = 8$	400	322	1,828	106	54	414	2.27	0.43	0.83
	$\beta = 9$	727	400	2,931	295	61	422	3.94	0.69	0.84
	$\beta = 10$	836	440	2,436	272	50	511	4.43	0.71	0.51
V-shape	$\beta = 1$	413	998	52.7	106	65	828	2.48	1.10	1.32
	$\beta = 2$	404	997	48.7	116	51	879	2.36	1.11	1.32
	$\beta = 3$	418	988	41.9	110	31	850	2.32	1.10	1.14
	$\beta = 4$	576	306	33.5	219	11	777	2.98	0.52	0.86
	$\beta = 5$	565	279	30.4	205	12	753	2.94	0.48	0.76
	$\beta = 6$	407	297	37.5	135	11	550	2.12	0.43	0.67
	$\beta = 7$	598	301	31.6	188	11	750	3.09	0.49	0.79
	$\beta = 8$	652	374	32.5	230	10	794	3.35	0.60	0.85
	$\beta = 9$	753	455	31.4	272	10	895	3.86	0.73	0.93
	$\beta = 10$	712	498	37.3	304	13	983	3.66	0.80	1.19

Table S3. Repeatability test of V-shaped feed spacer design for geometric parameters. Geometric design parameters and computational domain size are investigated in relation to a trade-off parameter value of β = 7. The design parameters are represented as $\boldsymbol{\beta}_1 = [L_S, W_S, \alpha, W_1, H_1, L_1]$ for V-shaped membrane modules, as shown in Figure S1. The computational domain size consists of channel length (L), width (W) and height (H).

	Geometric design parameters ($\boldsymbol{\beta}_1$)						Computational domain size		
	L_S (μm)	W_S (μm)	α (°)	W_1 (μm)	H_1 (μm)	L_1 (μm)	L (mm)	W (mm)	H (mm)
Optimized	598	301	31.6	188	11	750	3.09	0.49	0.79
Repeatability test	562	314	34.2	188	11	719	2.91	0.51	0.81

Table S4. Repeatability test of V-shaped feed spacer design for Sherwood numbers with respect to various Reynolds numbers. The tradeoff parameter in the objective function (F_1) is chosen as $\beta = 7$ to balance pressure drop and mass transfer.

Re	Sherwood number		Relative deviation
	Optimized	Repeatability test	
50	76	75	0.1%
100	98	98	0.4%
150	118	120	1.8%
200	138	142	2.5%
250	156	161	2.7%
300	173	177	2.9%
350	187	193	3.0%
400	201	207	3.1%
450	214	221	3.1%
500	226	234	3.2%
550	238	246	3.3%
600	249	258	3.3%

Table S5. Sensitivity analysis for geometric design parameters. The parameters, defined as $\boldsymbol{\beta}_1 = [L_S, W_S, \alpha, W_1, H_1, L_1]$, describe the V-shaped membrane modules under a cross-flow velocity of 0.1 m s^{-1}. A design optimized at β = 7 serves as the benchmark: $\boldsymbol{\beta}_{1,\text{opt}} = [598\,\mu\text{m}, 301\,\mu\text{m}, 31.6^\circ, 188\,\mu\text{m}, 11\,\mu\text{m}, 750\,\mu\text{m}]$ where $\bar{k}_m / \bar{k}_{m,0}$ and $\frac{\Delta P_c / L}{\Delta P_{c,0} / L_0}$ are 2.39 and 1.54 respectively. For the sensitivity analysis, each parameter is varied individually while the others remain fixed at $\boldsymbol{\beta}_{1,\text{opt}}$.

Parameter	Value								
L_S (μm)	400	600	800	1,000	1,200	1,400	1,600	1,800	2,000	
$\bar{k}_m / \bar{k}_{m,0}$	2.56	2.38	2.15	1.98	1.83	1.76	1.68	1.61	1.52	
$\frac{\Delta P_c / L}{\Delta P_{c,0} / L_0}$	2.20	1.54	1.22	1.09	1.00	0.90	0.81	0.74	0.68	
W_S (μm)	200	300	400	500	600	700	800	900	1,000	
$\bar{k}_m / \bar{k}_{m,0}$	2.62	2.40	2.19	2.02	1.87	1.72	1.66	1.62	1.52	
$\frac{\Delta P_c / L}{\Delta P_{c,0} / L_0}$	2.39	1.55	1.13	0.88	0.71	0.60	0.52	0.47	0.43	
α (°)	25	30	35	40	45	50	55	60	65	70
$\bar{k}_m / \bar{k}_{m,0}$	2.35	2.39	2.33	2.26	2.18	2.06	1.97	1.90	1.82	1.84
$\frac{\Delta P_c / L}{\Delta P_{c,0} / L_0}$	1.69	1.57	1.51	1.48	1.48	1.49	1.53	1.61	1.75	1.80
W_1 (μm)	100	200	300	400	500	600	700	800	900	1,000
$\bar{k}_m / \bar{k}_{m,0}$	2.18	2.41	2.53	2.61	2.65	2.68	2.70	2.71	2.71	2.73
$\frac{\Delta P_c / L}{\Delta P_{c,0} / L_0}$	1.04	1.63	2.39	3.29	4.34	5.54	6.86	8.25	9.69	11.14
H_1 (μm)	10	20	30	40	50	60	70	80	90	100
$\bar{k}_m / \bar{k}_{m,0}$	2.39	2.30	2.28	2.30	2.26	2.24	2.21	2.20	2.18	2.16
$\frac{\Delta P_c / L}{\Delta P_{c,0} / L_0}$	1.54	1.53	1.53	1.52	1.52	1.51	1.51	1.51	1.51	1.52
L_1 (μm)	300	400	500	600	700	800	900	1,000		
$\bar{k}_m / \bar{k}_{m,0}$	2.61	2.56	2.46	2.46	2.41	2.36	2.33	2.27		
$\frac{\Delta P_c / L}{\Delta P_{c,0} / L_0}$	3.26	2.49	2.05	1.75	1.59	1.51	1.46	1.43		

Table S6. The range of system design parameters for two-stage ultrapermeable seawater reverse osmosis.

Design Parameters			Maximum	Minimum
Membrane module	Number of feed spacers per element	n_{sp}	30	5
First-stage RO system	Transmembrane pressure	ΔP_0 (bar)	$\Delta P_{0,\ min}$ (Refer to our previous work[8])	0
	Number of modules per pressure vessel	$n_{mem,\ 1}$	20	4
	Number of pressure vessels	$N_{pv,\ 1}$	30	5
Second-stage RO system	Transmembrane pressure	ΔP_1 (bar)	$\Delta P_{0,\ min}$ +10 bar	$\Delta P_{0,\ min}$
	Number of modules per pressure vessel	$n_{mem,\ 2}$	20	4
	Number of pressure vessels	$N_{pv,\ 2}$	$N_{pv,\ 1}$	$\text{ceil}\left(N_{pv,\ 1}/3\right)^{\#}-1$
Membrane properties	Water permeability	L_p (L m^{-2} h^{-1} bar^{-1})	100	1
	Salt permeability	B (L m^{-2} h^{-1})	$0.1\times L_p\times\text{bar}$	0.1

[#] ceil: rounding towards positive infinity

Table S7. Optimized two-stage ultrapermeable membrane systems with a limited concentration polarization (no more than 1.20). The analysis is based on a feed salinity of 35,000 ppm, a water recovery rate of 50%, pump efficiency of 85%, and energy recovery efficiency of 95%. The original results are from a conventional one-stage SWRO system with a commercial membrane module under standard engineering conditions. In contrast, optimized results are obtained from a two-stage SWRO system using ultrapermeable membranes and optimized spacer configurations for tradeoff parameter c_m values (40, 220, and 400 \$ m^{-2}), with a maximum concentration polarization factor of 1.20. The geometric specifications of the commercial membrane module, with a 28 mil feed spacer, are detailed in previous work.[10]

Parameters				Value			
				Original	Optimized c_m = 40 \$ m^{-2}	c_m = 220 \$ m^{-2}	c_m = 400 \$ m^{-2}
Design parameters	Geometrical parameters of feed spacers		L_1 (μm)	---	750	750	750
			W_1 (μm)	---	188	188	188
			H_1 (μm)	---	11	11	11
			α (°)	---	31.6	31.6	31.6
			L_S (μm)	---	598	598	598
			W_S (μm)	---	301	301	301
	First-stage RO system	Inlet transmembrane pressure	ΔP_0 (bar)	65.0	40.7	42.7	44.0
		Number of modules per pressure vessel	$n_{mem,1}$	7	4	4	4
		Number of pressure vessels	$N_{pv,1}$	30	30	14	23
	Second-stage RO system	Inlet transmembrane pressure	ΔP_1 (bar)	---	56.7	57.5	58.9
		Number of modules per pressure vessel	$n_{mem,2}$	---	4	4	4
		Number of pressure vessels	$N_{pv,2}$	---	17	9	15
	Module parameters	Number of feed spacers per element	n_{sp}	23	19	23	11
	Membrane properties	Water permeability	L_p (lmh bar^{-1})	1.00	15.73	25.34	26.23
		Salt permeability	B (lmh)	0.05	0.48	0.79	1.01
Computational results	Average permeate salinity		$\bar{w}_p$ (ppm)	145	495	498	499
	Average water flux		$\bar{J}_w$ (lmh)	19	52	88	111
	Maximum CPF		Max (CPF)	1.09	1.16	1.20	1.20
	Recovery rate		$R_{r,0}$	0.50	0.50	0.50	0.50
	Membrane area		A_{tot} (m^2)	7,804	2,886	1,709	1,351
	Specific energy consumption		SEC (kWh m^{-3})	2.30	1.68	1.78	1.88

Table S8. Optimized two-stage ultrapermeable membrane systems with a limited concentration polarization (no more than 1.25). The analysis is based on a feed salinity of 35,000 ppm, a water recovery rate of 50%, pump efficiency of 85%, and energy recovery efficiency of 95%. The original results are from a conventional one-stage SWRO system with a commercial membrane module under standard engineering conditions. In contrast, optimized results are obtained from a two-stage SWRO system using ultrapermeable membranes and optimized spacer configurations for tradeoff parameter c_m values (40, 220, and 400 \$ m^{-2}), with a maximum concentration polarization factor of 1.25. The geometric specifications of the commercial membrane module, with a 28 mil feed spacer, are detailed in previous work.[10]

Parameters				Value			
				Original	Optimized c_m = 40 \$ m^{-2}	c_m = 220 \$ m^{-2}	c_m = 400 \$ m^{-2}
Design parameters	Geometrical parameters of feed spacers		L_1 (μm)	---	750	750	750
			W_1 (μm)	---	188	188	188
			H_1 (μm)	---	11	11	11
			α (°)	---	31.6	31.6	31.6
			L_S (μm)	---	598	598	598
			W_S (μm)	---	301	301	301
	First-stage RO system	Inlet transmembrane pressure	ΔP_0 (bar)	65.0	40.9	42.2	43.3
		Number of modules per pressure vessel	$n_{mem,1}$	7	4	4	4
		Number of pressure vessels	$N_{pv,1}$	30	29	22	8
	Second-stage RO system	Inlet transmembrane pressure	ΔP_1 (bar)	---	56.7	57.5	58.1
		Number of modules per pressure vessel	$n_{mem,2}$	---	4	4	4
		Number of pressure vessels	$N_{pv,2}$	---	16	13	5
	Module parameters	Number of feed spacers per element	n_{sp}	23	22	14	30
	Membrane properties	Water permeability	L_p (lmh bar^{-1})	1.00	13.68	34.90	42.33
		Salt permeability	B (lmh)	0.05	0.44	0.76	1.06
Computational results	Average permeate salinity		$\bar{w}_p$ (ppm)	145	500	441	495
	Average water flux		$\bar{J}_w$ (lmh)	19	47	95	119
	Maximum CPF		Max (CPF)	1.09	1.15	1.22	1.25
	Recovery rate		$R_{r,0}$	0.50	0.5	0.50	0.50
	Membrane area		A_{tot} (m^2)	7,804	3,199	1,583	1,260
	Specific energy consumption		SEC (kWh m^{-3})	2.30	1.67	1.78	1.86

Table S9. Optimized two-stage ultrapermeable membrane systems with a limited concentration polarization (no more than 1.30). The analysis is based on a feed salinity of 35,000 ppm, a water recovery rate of 50%, pump efficiency of 85%, and energy recovery efficiency of 95%. The original results are from a conventional one-stage SWRO system with a commercial membrane module under standard engineering conditions. In contrast, optimized results are obtained from a two-stage SWRO system using ultrapermeable membranes and optimized spacer configurations for tradeoff parameter c_m values (40, 220, and 400 \$ m^{-2}), with a maximum concentration polarization factor of 1.30. The geometric specifications of the commercial membrane module, with a 28 mil feed spacer, are detailed in previous work.[10]

Parameters				Value			
				Original	Optimized c_m = 40 \$ m^{-2}	c_m = 220 \$ m^{-2}	c_m = 400 \$ m^{-2}
Design parameters	Geometrical parameters of feed spacers		L_1 (μm)	---	750	750	750
			W_1 (μm)	---	188	188	188
			H_1 (μm)	---	11	11	11
			α (°)	---	31.6	31.6	31.6
			L_S (μm)	---	598	598	598
			W_S (μm)	---	301	301	301
	First-stage RO system	Inlet transmembrane pressure	ΔP_0 (bar)	65.0	41.0	42.8	43.4
		Number of modules per pressure vessel	$n_{mem,1}$	7	4	4	4
		Number of pressure vessels	$N_{pv,1}$	30	25	20	24
	Second-stage RO system	Inlet transmembrane pressure	ΔP_1 (bar)	---	56.8	57.4	59.1
		Number of modules per pressure vessel	$n_{mem,2}$	---	4	4	4
		Number of pressure vessels	$N_{pv,2}$	---	14	14	14
	Module parameters	Number of feed spacers per element	n_{sp}	23	24	14	10
	Membrane properties	Water permeability	L_p (lmh bar^{-1})	1.00	15.90	62.42	59.05
		Salt permeability	B (lmh)	0.05	0.36	0.22	0.43
Computational results	Average permeate salinity		$\bar{w}_p$ (ppm)	145	394	129	200
	Average water flux		$\bar{J}_w$ (lmh)	19	50	98	123
	Maximum CPF		Max (CPF)	1.09	1.16	1.30	1.29
	Recovery rate		$R_{r,0}$	0.50	0.50	0.50	0.50
	Membrane area		A_{tot} (m^2)	7,804	3,025	1,538	1,228
	Specific energy consumption		SEC (kWh m^{-3})	2.30	1.68	1.79	1.88

Table S10. Repeatability test of system design. The analysis is based on a feed salinity of 35,000 ppm, a water recovery rate of 50%, pump efficiency of 85%, and energy recovery efficiency of 95%. Optimized system performance and repeatability are evaluated in a two-stage seawater reverse osmosis (SWRO) using ultrapermeable membranes and optimized spacer configurations for tradeoff parameter c_m values of 310, 340, 370 and 400 \$ m^{-2}, with a maximum concentration polarization factor of 1.25.

c_m (\$ m^{-2})	SEC (kWh m^{-3})		Relative deviation	$\bar{J}_w$ (lmh)		Relative deviation	$\bar{w}_p$ (ppm)		Relative deviation
	Optimized	Repeatability test		Optimized	Repeatability test		Optimized	Repeatability test	
310	1.84	1.82	0.8%	112.2	105.5	5.9%	490	480	2.1%
340	1.82	1.84	1.3%	107.3	114.6	6.8%	500	490	2.0%
370	1.88	1.85	1.3%	121.2	117.2	3.3%	499	497	0.4%
400	1.86	1.88	1.1%	119.2	122.5	2.8%	495	499	0.8%

Table S11. Comparison of normalized SEC (NSEC) breakdowns for batch reverse osmosis.[10]

NSEC = SEC/π_0 (π_0 is feed osmotic pressure)

Contributing terms in NSEC	Batch RO
Thermodynamics, $NSEC_1$	$-\ln\left(1-Y_{\text{tot}}\right)/Y_{\text{tot}}$
Design flux, $NSEC_2$	$\bar{J}_{\text{W}}/\left(L_{\text{P}}\pi_0\right)$
Flow resistance, $NSEC_3$	$\frac{\alpha_2}{\pi_0}\frac{\frac{1}{t_1+1}\frac{1-\left(1-Y_{\text{SP}}\right)^{t_1+1}}{Y_{\text{SP}}}+\frac{Y_{\text{SP}}\left(1-Y_{\text{tot}}\right)}{Y_{\text{tot}}}}{Y_{\text{SP}}}$
Concentration polarization, $NSEC_4$	$\left(\text{CPF}-1\right)\left[-\frac{\ln\left(1-Y_{\text{tot}}\right)}{Y_{\text{tot}}}-\ln\left(1-Y_{\text{tot}}\right)\left(\frac{1}{f_{n_{\text{mem}}}}-1\right)\right]$
Salt retention, $NSEC_5$	$-\ln\left(1-Y_{\text{tot}}\right)/\left(1/f-1\right)$
Pump inefficiency, $NSEC_6$	$\left(1/\eta_{\text{pump}}-1\right)\sum_{i=1}^{5}\text{NSEC}_i$

Table S12. Batch ultrapermeable membrane systems with optimized spacer. The analysis is based on a feed salinity of 35,000 ppm, a water recovery rate of 50%, pump efficiency of 85%. The original results are from a conventional one-stage SWRO system utilizing a commercial membrane module under standard engineering conditions. In contrast, optimized results are obtained from a batch SWRO system incorporating ultrapermeable membranes and optimized spacer configurations. The geometric specifications of the commercial membrane module, with a 28 mil feed spacer, are detailed in previous work.[10]

**Parameters**				**Value**			
				Original	Optimized spacer + UPM + batch		
					Option 1	Option 2	Option 3
Design parameters	Geometrical parameters of feed spacers		L_1 (μm)	---	750	750	750
			W_1 (μm)	---	188	188	188
			H_1 (μm)	---	11	11	11
			α (°)	---	31.6	31.6	31.6
			L_S (μm)	---	598	598	598
			W_S (μm)	---	301	301	301
	RO system	Inlet transmembrane pressure	ΔP_0 (bar)	65.0	Varying as a function of time[11]		
		Number of modules per pressure vessel	n_{mem}	7	2	2	2
		Number of pressure vessels	N_{pv}	30	70	30	25
	Module parameters	Number of feed spacers per element	n_{sp}	23	14	14	14
	Membrane properties	Water permeability	L_p (lmh bar^{-1})	1.00	34.90	34.90	34.90
Computational results	Average water flux		$\bar{J}_w$ (lmh)	19	95	221	265
	Maximum CPF		Max (CPF)	1.09	1.14	1.21	1.23
	Recovery rate		$R_{r,0}$	0.50	0.50	0.50	0.50
	Membrane area		A_{tot} (m^2)	7,804	1,583	679	565
	Specific energy consumption		SEC (kWh m^{-3})	2.30	1.68	2.04	2.18

Table S13. Batch ultrapermeable membrane systems with commercial spacer. The analysis is based on a feed salinity of 35,000 ppm, a water recovery rate of 50%, pump efficiency of 85%. The original results are from a conventional one-stage SWRO system utilizing a commercial membrane module under standard engineering conditions. In contrast, optimized results are obtained from a batch SWRO system incorporating ultrapermeable membranes and commercial spacer configurations. The geometric specifications of the commercial membrane module, with a 28 mil feed spacer, are detailed in previous work.[10]

**Parameters**				**Value**			
				Original	Commercial spacer + UPM + batch		
					Option 1	Option 2	Option 3
Design parameters	RO system	Inlet transmembrane pressure	ΔP_0 (bar)	65.0	Varying as a function of time[11]		
		Number of modules per pressure vessel	n_{mem}	7	1	1	1
		Number of pressure vessels	N_{pv}	30	42	18	15
	Module parameters	Number of feed spacers per element	n_{sp}	23	23	23	23
	Membrane properties	Water permeability	L_p (lmh bar^{-1})	1.00	34.90	34.90	34.90
Computational results	Average water flux		$\bar{J}_W$ (lmh)	19	96	224	269
	Maximum CPF		Max (CPF)	1.09	1.26	1.42	1.46
	Recovery rate		$R_{r,0}$	0.50	0.50	0.50	0.50
	Membrane area		A_{tot} (m^2)	7,804	1,561	669	557
	Specific energy consumption		SEC (kWh m^{-3})	2.30	1.85	2.30	2.45

Supplementary References

1. Ruan X., Jiang P., Zhou Q., et al. (2020). Variable-fidelity probability of improvement method for efficient global optimization of expensive black-box problems. *Structural and Multidisciplinary Optimization* **62**:3021-3052. DOI:10.1007/s00158-020-02646-9
2. Jones D. R., Schonlau M. and Welch W. J. (1998). Efficient global optimization of expensive black-box functions. *Journal of Global Optimization* **13**:455–492. DOI:10.1023/A:1008306431147
3. Blockeel H., Kristian K., Nijssen S., et al. (2013). Machine learning and knowledge discovery in databases (Springer). 10.1007/978-3-642-40988-2.
4. Bull A. D. (2011). Convergence rates of efficient global optimization algorithms. *Journal of Machine Learning Research* **12**:2879-2904. DOI:10.5555/1953048.2078198
5. Li M. H., Bui T. and Chao S. (2016). Three-dimensional CFD analysis of hydrodynamics and concentration polarization in an industrial RO feed channel. *Desalination* **397**:194-204. DOI:10.1016/j.desal.2016.07.005
6. Guillen G. and Hoek E. M. V. (2009). Modeling the impacts of feed spacer geometry on reverse osmosis and nanofiltration processes. *Chem. Eng. J.* **149**:221–231. DOI:10.1016/j.cej.2008.10.030
7. Li M. H. (2021). Residence time distribution in RO channel. *Desalination* **506**:115000. DOI:10.1016/j.desal.2021.115000
8. Luo J., Li M., Hoek E. M. V., et al. (2023). Supercomputing and machine learning-aided optimal design of high permeability seawater reverse osmosis membrane systems. *Sci. Bull.* **68**:397-407. DOI:10.1016/j.scib.2023.01.039
9. Geraldes V. and Afonso M. D. (2006). Generalized mass-transfer correction factor for nanofiltration and reverse osmosis. *AIChE J.* **52**:3353-3362. DOI:10.1002/aic.10968
10. Bucs S. S., Radu A. I., Lavric V., et al. (2014). Effect of different commercial feed spacers on biofouling of reverse osmosis membrane systems: a numerical study. *Desalination* **343**:26–37. DOI:10.1016/j.desal.2013.11.007
11. Li M. H. (2020). Effects of finite flux and flushing efficacy on specific energy consumption in semi-batch and batch reverse osmosis processes. *Desalination* **496**:114646. DOI:10.1016/j.desal.2020.114646
