# Supplementary material for: Intelligent design breaks the trade-off between energy efficiency and water flux in ultrafast seawater desalination
Source: Innovation (Camb). 2026 Jan 7;7(5):101262. doi: 10.1016/j.xinn.2026.101262 (PMC13147988; doi:10.1016/j.xinn.2026.101262)
Supplement: Document S2. Article plus supplemental information [file mmc2.pdf]

OPEN ACCESS

Intelligent design breaks the trade-off between energy efficiency and water flux in ultrafast seawater desalination

Jiu Luo,[1,2] Xing Liu,[1] Jin Wang,[1,2] and Yi Heng[3,*]
*Correspondence: hengyi@mail.sysu.edu.cn

Received: May 20, 2025; Accepted: January 5, 2026; Published Online: January 7, 2026; https://doi.org/10.1016/j.xinn.2026.101262

© 2026 The Authors. Published by Elsevier Inc. on behalf of Youth Innovation Co., Ltd. This is an open access article under the CC BY license (http://creativecommons.org/licenses/by/4.0/).

GRAPHICAL ABSTRACT

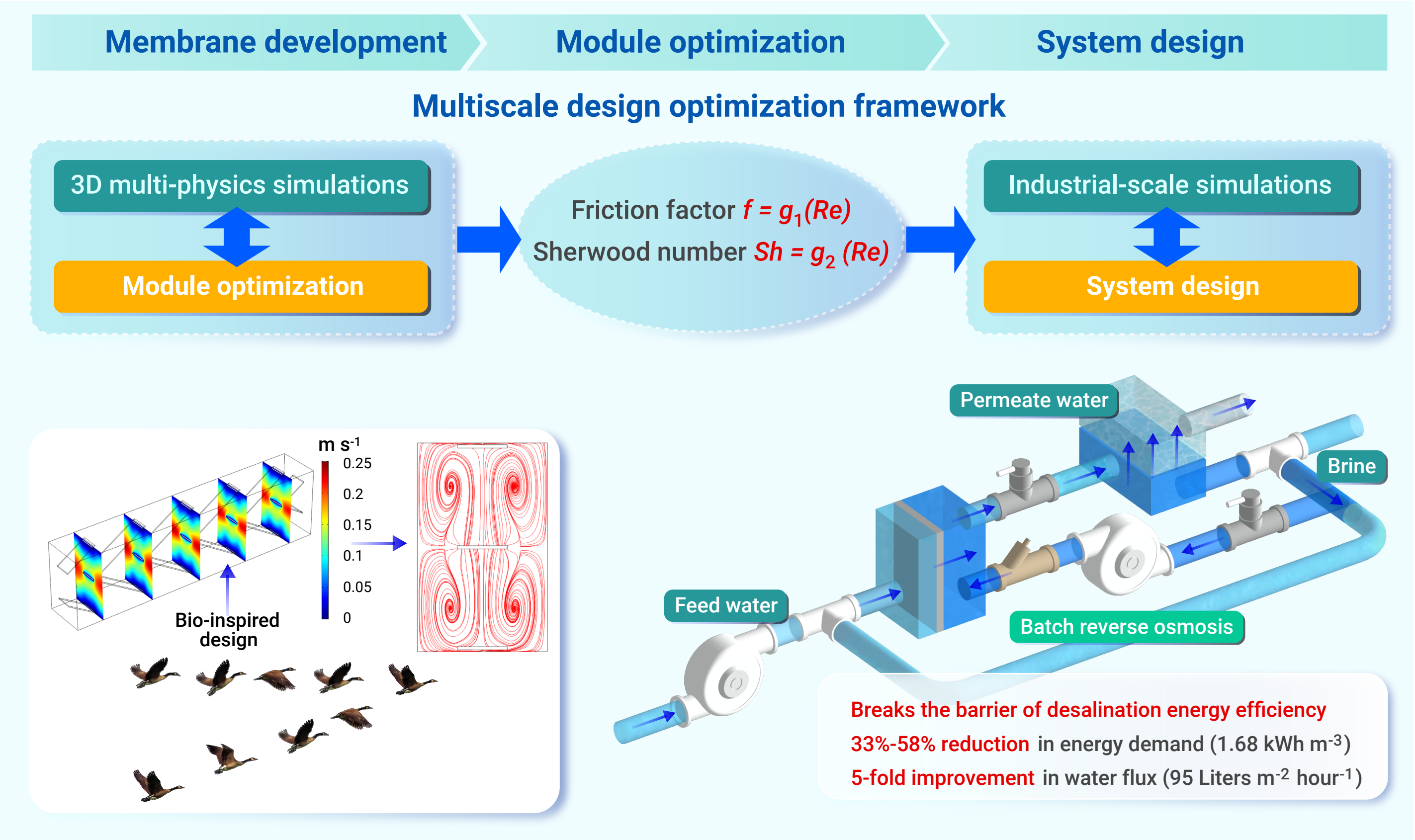

PUBLIC SUMMARY

- A multiscale intelligent design framework is proposed for ultrafast seawater reverse osmosis desalination.
- A bio-inspired module is optimized to achieve a best trade-off between mass transfer and flow resistance.
- Optimized ultrapermeable membrane systems significantly improve desalination energy efficiency.

OPEN ACCESS

Science X ARTICLE

Intelligent design breaks the trade-off between energy efficiency and water flux in ultrafast seawater desalination

Jiu Luo,[1,2] Xing Liu,[1] Jin Wang,[1,2] and Yi Heng[3,*]

[1]School of Future Science and Engineering, Soochow University, Suzhou 215222, China

[2]Key Laboratory of General Artificial Intelligence and Large Models in Provincial Universities, Soochow University, Suzhou 215222, China

[3]School of Computer Science and Engineering, Sun Yat-sen University, Guangzhou 510006, China

*Correspondence: hengyi@mail.sysu.edu.cn

Received: May 20, 2025; Accepted: January 5, 2026; Published Online: January 7, 2026; https://doi.org/10.1016/j.xinn.2026.101262

© 2026 The Authors. Published by Elsevier Inc. on behalf of Youth Innovation Co., Ltd. This is an open access article under the CC BY license (http://creativecommons.org/licenses/by/4.0/).

Citation: Luo J., Liu X., Wang J., et al., (2026). Intelligent design breaks the trade-off between energy efficiency and water flux in ultrafast seawater desalination. The Innovation **7(5)**, 101262.

Enhancing the energy efficiency for energy-intensive seawater desalination technologies is imperative to sustainably mitigate water scarcity while reducing carbon footprints. This work presents a transformative advance in reverse osmosis desalination technology by fundamentally redefining the long-standing trade-off between energy efficiency and water production efficiency. Through the synergistic integration of bio-inspired ultra-permeable membrane module with state-of-the-art batch reverse osmosis, we demonstrate unprecedented performance—achieving a specific energy consumption of 1.68 kWh m^{-3} while delivering an average water flux of 95 L m^{-2} h^{-1}. This represents a 33%–58% reduction in energy demand and a 5-fold improvement in water flux compared with conventional seawater reverse osmosis desalination plants (2.5–4 kWh m^{-3} and 15 L m^{-2} h^{-1}), challenging the prevailing assumption that increased membrane permeability offers only marginal efficiency benefits. This work can further guide the development of advanced membrane materials and energy-efficient desalination technologies, with potential applications in desalination and zero/minimal liquid discharge systems.

INTRODUCTION

Global freshwater resources are becoming increasingly scarce. Desalination and wastewater reclamation and reuse through purification are expected to be the primary water resource augmentation technologies in the coming decades.[1] Over the past three decades, seawater desalination capacity has increased more than 8-fold, now reaching 100 million cubic meters per day.[2] Reverse osmosis (RO) is one of the most advanced desalination technologies currently available, accounting for over 60% of the global desalination market.

The specific energy consumption (SEC) for typical seawater RO (SWRO) systems in engineering (feed salinity 35 g/L, recovery rate 50%) ranges from 2.5 to 4.0 kWh m^{-3} which amounts to 71% of the total plant energy consumption.[3] The rest of the energy consumption distribution is as follows: 11% is used for pre-treatment, 5% for delivering product water, 5% for intake, and 8% for other facilities.[3] The theoretical SEC is approximately 1.1 kWh m^{-3} under the same feed salinity and recovery rate conditions. The comprehensive evaluation of SEC in SWRO systems indicated that it enables energy savings of approximately 69% through technological improvements in, e.g., pump efficiency, membrane permeability, mass transfer ability in membrane channel, and pre- and post-treatment.[4] Further implementation of innovative system configurations, such as batch SWRO, could yield energy savings of up to 82%.[4] The potential of further energy consumption reduction is even greater in zero/minimal liquid discharge systems.[5]

Ultrapermeable membrane (UPM) materials such as graphene/graphene oxide,[6,7] carbon nanotube,[8–10] improved polyamide,[11–13] aquaporins,[14–16] and fluorous nanochannels[17] have undergone rapid development in recent years, with membrane permeabilities significantly improved over commercial thin-film composite SWRO membranes. For example, the developed biomimetic membranes can achieve a high water flux of 75 L m^{-2} h^{-1} (lmh) for SWRO (feed salinity of 35,000 ppm) with a high salt rejection (99.5% for sodium chloride or 91.4% for boron).[18] The large-area graphene-nanomesh/single-walled carbon nanotube membrane reported exhibits excellent hydraulic permeability, with a rate of 97.6 lmh bar^{-1} for NaCl solution compared with 110.6 lmh bar^{-1} for pure water.[9] It also demonstrates a high rejection ratio for salt ions and organic molecules, along with outstanding mechanical strength. In addition to water permeability and salt rejection, membrane development increasingly focuses on improving chlorine resistance, boron rejection, and anti-fouling properties.[19]

However, the applications of next-generation UPMs face several primary challenges. The first challenge lies in scaling up the production of UPMs that maintain an optimal balance between salt rejection, mechanical durability, and cost-effectiveness, particularly when advanced materials such as graphene oxide or aquaporins are employed. The second challenge lies in optimizing the membrane module to achieve a 2- to 5-fold enhancement in the boundary layer mass transfer coefficient.[20] The experimental results indicate that the mass transfer coefficient with the static mixing spacer is 20% higher than that of the conventional spacer.[21] The three-dimensional (3D) computational fluid dynamics (CFD) simulations reveal that twisted spacers exhibit a mass transfer (Sherwood number) approximately 55% higher and a friction factor 8% lower compared with conventional spacers.[22] Using the machine learning-aided optimization approach in our previous work,[23] the boundary layer mass transfer coefficient for the optimized feed spacer is improved by approximately 21.1%, accompanied by a 23.4% increase in axial pressure drop per meter compared with the commercial non-woven spacer. Therefore, doubling the boundary layer mass transfer coefficient with a moderate flow resistance (or pressure drop) penalty is challenging for typical spiral wound membrane module.[20]

In improving the energy efficiency of seawater desalination, system design offers considerable potential.[24] In contrast to traditional steady-state RO operating under constant pressure, dynamic RO systems, such as batch[25–27] or semi-batch[28,29] configurations, operate with time-varying operating pressures. These systems have the potential for energy savings and could provide more uniform water flux distribution, effectively mitigating concentration polarization and membrane fouling. Therefore, UPM in conjunction of innovative membrane module and dynamics RO has the potential to significantly enhance water flux and reduce SEC.

MATERIALS AND METHODS

Overview of multiscale design optimization framework

In this work we propose a multiscale optimization framework that integrates membrane permeability, feed spacer design at sub-millimeter scale, and system design (two-stage and batch configurations) at industrial scale (meter scale). A Bayesian-driven pattern search approach is developed for optimal design of feed spacers that is based on the bio-inspired V-shaped spacer proposed in our previous work.[30] The optimization approach is employed to balance mass transfer and flow resistance and identify optimal geometric parameters for solving a 3D multi-physics constrained optimization problem, incorporating nonlinear channel flow and mass transport. The two-stage and batch SWROs are designed for maximizing the benefit by reducing SEC and enhancing average water flux (or reducing the required membrane area). The 3D multi-physics models are solved with COMSOL Multiphysics 5.3a, whereas the spacer optimization and system design are conducted in MATLAB.

Optimal membrane module design

Optimization problem description. The mathematical formulation of the module design optimization problem can be described as follows:

$$\min_{\beta_1} F_1 \quad s.t.\ \mathbf{H}_1 = \mathbf{0}. \qquad \text{(Equation 1)}$$

Design parameters (β_1) of the membrane module, including distance parameters, size parameters, and a shape parameter, are expressed as $\beta_1 = [L_S, W_S, a_1, W_1, H_1, H_2]$ for cosine-shaped, $\beta_1 = [L_S, W_S, a_2, W_1, H_1, H_2]$ for parabolic-shaped, and $\beta_1 = [L_S, W_S, \alpha, W_1,$

www.the-innovation.org

H_1, L_1] for V-shaped membrane modules, as shown in Figure S1. The objective function (F_1) is mathematically defined as follows:

$$F_1 = \frac{APLR}{(\bar{k}_m/\bar{k}_{m,0})^{\beta}} + \lambda\left(e^{0.1APLR} - 1\right), \quad \text{(Equation 2)}$$

where $APLR$ represents the ratio of the axial pressure drops per meter between the designed membrane module in this work and the commercial membrane module, mathematically defined as follows:

$$APLR = \frac{\Delta P_c/L}{\Delta P_{c,0}/L_0}, \quad \text{(Equation 3)}$$

The trade-off parameter β is applied to balance mass transfer and pressure drop (or flow resistance). Our previous study[30] showed that an increase in β leads to an improvement in the membrane module's mass transfer coefficient. However, this improvement is offset by a rapidly increasing pressure drop, which reduces the overall benefit of the enhanced mass transfer. In this study, we introduce a penalty term in Equation 2 with the parameter $\lambda = 0.05$ to prevent excessive increases in pressure drop. A hybrid algorithm combining Bayesian optimization and pattern search is developed to solve the 3D multi-physics model constrained optimization problem, integrating nonlinear channel fluid flow and mass transport, for the purpose of optimizing the geometric structure of the membrane module. A more detailed description of the hybrid algorithm can be found in Bayesian-driven pattern search.

Bayesian-driven pattern search. Bayesian optimization, a well-established technique, serves as a quintessential example of global black-box optimization algorithms,[31] which has been widely applied across various fields, such as reactor designs,[32] inverse heat transfer problems,[33] and parameter estimation of partial differential equation models.[34] The objective functions it seeks to optimize often lack fundamental mathematical properties, such as convexity and differentiability, and their evaluation is generally computationally expensive. The Bayesian algorithm assumes a model for the unknown objective function before optimization, with Gaussian processes commonly used due to their consistency over compact sets and closed-form posterior distribution. It iteratively selects the point with the highest acquisition function value.

The concept of pattern search was first introduced by Hooke and Jeeves in 1961,[35] and later Kolda et al. developed a unified framework, demonstrating its strong adaptability in addressing complex engineering problems.[36] A key advantage of pattern search algorithm is its independence from derivative information, making it particularly well suited for engineering optimization tasks. The algorithm iteratively executes search and polling steps based on the pattern vector direction and current grid size until the predefined convergence criteria are satisfied.

The developed hybrid optimization approach integrates the Bayesian algorithm with pattern search, offering enhanced robustness and computational efficiency. Its capabilities make it particularly well suited for engineering optimization challenges that involve costly computational simulations. For further details on Bayesian optimization and the pattern search algorithm, please refer to Note S1.

Optimal design of two-stage UPM systems

Optimization problem description. The optimal design of two-stage UPM systems can be formulated as

$$\begin{aligned} &\min_{\boldsymbol{\beta}_2} \; F_2 \\ &s.t. \; \mathbf{H}_2 = \mathbf{0}, \\ &\mathbf{J} \leq \mathbf{0}. \end{aligned} \quad \text{(Equation 4)}$$

The optimal design parameters (β_2) are determined by minimizing the objective function (F_2) subject to system-level model constraints and inequality constraints ($\mathbf{J} \leq \mathbf{0}$) including limits on concentration polarization factor (CPF), maximum allowable average permeate salinity, minimum required water flux, and the specified range for design variables. F_2 represents a balance between the annualized capital cost of the membrane and the energy cost per cubic meter of permeate, formulated as follows.

$$F_2 = \frac{A_{tot} c_m F_a}{Q_p t_{op}} + c_e \cdot \text{SEC}. \quad \text{(Equation 5)}$$

This work employs the membrane cost per square meter (c_m) as a control variable to balance the trade-off between SEC and the total required membrane area (A_{tot}). The parameters c_e, F_a, and t_{op} correspond to the energy cost per kilowatt-hour, the annual amortization factor, and the total operating hours per year, respectively, with their values sourced from previous research.[37] The SEC for two-stage RO is calculated by

$$\text{SEC} = \frac{Q_0 \Delta P_0 + Q_1\left(P_{1,in} - P_{1,out}\right) - \eta_R Q_2 \Delta P_2}{36 \eta_{pump} Q_p}, \quad \text{(Equation 6)}$$

where Q_0, Q_1, and Q_2 represent the flow rates at the inlet of the first stage, the outlet of the first stage, and the outlet of the second stage, respectively. Similarly, ΔP_0, ΔP_1, and ΔP_2 denote the transmembrane pressures at the corresponding locations. The hydraulic pressures at the inlet of the second stage and the outlet of the first stage are given by $P_{1,\,in}$ and $P_{1,\,out}$, respectively. The permeate flow rate is denoted as Q_p. In this study, the pump efficiency and energy recovery device efficiency for the seawater SWRO system are specified as 85% and 95%, respectively.

The design parameters at system-level include water permeability (L_P) and salt permeability (B), number of pressure vessels ($N_{pv,\,1}$, $N_{pv,\,2}$), number of modules per vessel ($n_{mem,\,1}$, $n_{mem,\,2}$), transmembrane pressures (ΔP_0, ΔP_1) at first and second stages, respectively, and number of spacer sheets per module (n_{sp}). To obtain the optimal system configuration, the optimization problem (4) is solved as a nonlinear mixed-integer programming problem. The optimization process is conducted using the genetic algorithm toolbox, GATBX.[38] Further details on the system model are available in our previous work.[23]

Optimal design of batch UPM systems

The normalized SEC (NSEC) considering concentration polarization and expressed as SEC/π_0 (where π_0 represents the feed osmotic pressure) is estimated as follows[29]

$$\text{NSEC}_{CP} = (\text{CPF} - 1)\left[-\frac{\ln(1 - Y_{tot})}{Y_{tot}} - \ln(1 - Y_{tot})\left(\frac{1}{f_{n_{mem}}} - 1\right)\right]. \quad \text{(Equation 7)}$$

The total recovery rate is represented by Y_{tot}, while $f_{n_{mem}}$ is the flushing efficacy when n_{mem} RO modules are arranged in series. $f_{n_{mem}}$ can be estimated using the simulated results of a 3D transient CFD model in a five spacer-filled channel incorporating convolution operation.[39] CPF can be estimated by $\exp\left(\bar{J}_W/\bar{k}_{m,per}\right)$, where $\bar{J}_W$ and $\bar{k}_{m,per}$ denote the average water flux and the estimated cell-averaged mass transfer coefficient using permeable wall model, respectively. $\bar{k}_{m,per}$ can be converted by the empirical correlation[40] incorporating $\bar{k}_m$ calculated by CFD with the use of an impermeable wall boundary condition. The NSEC associated with frictional losses can be estimated as follows[29]

$$\text{NSEC}_{friction} = \frac{\alpha_2}{\pi_0} \frac{\frac{1}{t_1+1}\frac{1-(1-Y_{SP})^{t_1+1}}{Y_{SP}} + \frac{Y_{SP}(1-Y_{tot})}{Y_{tot}}}{Y_{SP}}, \quad \text{(Equation 8)}$$

The single-pass recovery is denoted as Y_{SP}. The system-level pressure drop (α_2) can be determined by the relations $-\Delta P_c/L = k_1 Q^{t_1}$, where the parameters k_1 and t_1 are derived through regression analysis of CFD simulation data.

RESULTS

Multiscale optimization framework

UPM technology is constrained by factors such as concentration polarization and membrane fouling, highlighting the need for redesigned membrane modules (Figure 1A).[41] Herein, we present a multiscale design optimization framework (Figure 1B) that combines membrane module optimization (or feed spacer design, Figures 1C–1F) with system design (two-stage and batch designs), incorporating UPMs for SWRO desalination. A Bayesian-driven pattern search approach is developed to optimize the membrane module, achieving a best trade-off between mass transfer and flow resistance. The Bayesian method guarantees strong global convergence, aiding in the acceleration of the pattern search approach without the need for derivative information or a well-defined initial estimate. More introduction on the hybrid optimization approach can be found in the materials and methods and Note S1.

In the module design objective function, the trade-off parameter β is employed to balance mass transfer and flow resistance. To mitigate excessive flow resistance, a penalty term is introduced in the optimization objective function to penalize pressure drop (Equation 2). It enables the optimal design of the membrane module to maximize mass transfer enhancement while applying an appropriate flow resistance penalty through adjustment β. Based on CFD simulations of the optimal membrane module across various Reynolds numbers (Re), the relationships between the Darcy friction factor ($f = g_1$ (Re)) and Sherwood number ($Sh = g_2$ (Re)) with respect to Re can be determined (Figure 1B).

In the system design, this work focuses on the operational and capital costs of membrane separation, particularly electricity and membrane expenses in membrane separation. A constant feed flow rate is maintained in our design; therefore, pre-treatment cost is fixed. Capital investment increases in the order

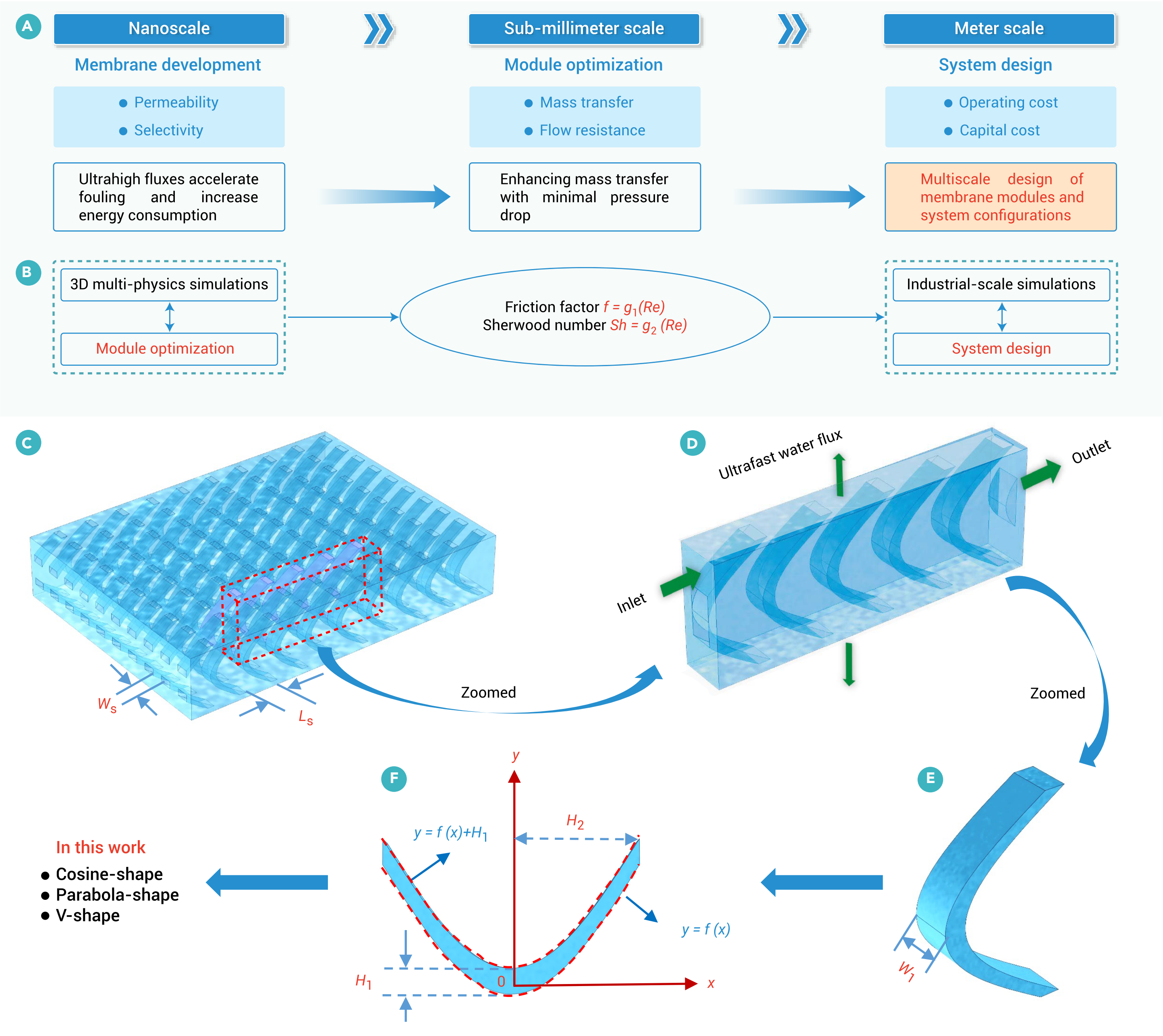

Figure 1. Overview of multiscale design optimization (A) Trade-off between permeability and selectivity for membrane development, mass transfer, and flow resistance for module optimization, and operating cost (i.e., specific energy consumption of this work) and capital cost (i.e., required membrane area of this work) for system design. (B) Multiscale design optimization coupling module optimization (or feed spacer design) at sub-meter scale and system design at meter scale (industrial scale). (C) A small piece of spacer sheet. (D) Computational domain of three-dimensional multi-physics model. (E) A spacer cell. (F) Lateral view of the spacer cell. The feed spacer-filled channel is defined by distance parameters (e.g., L_S, W_S), size parameters (e.g., H_1, H_2, W_1), and shape function ($y = f(x)$). This work explores three shape functions—cosine, parabolic, and V shaped—along with additional details on the parametric geometric models in Figure S1.

of single-stage, batch, and multi-stage RO. Theoretically, batch RO can match the energy efficiency of an idealized infinite-stage RO system, which is economically impractical to construct. Moreover, the system design solutions are obtained (Figure 1B) by solving the system-level model constrained optimization problem (see materials and methods). The optimization objectives in system design include SEC (operating cost) and required membrane area (capital cost), the membrane cost of which per m^2 (c_m) is considered as a trade-off parameter to balance both objectives. The system design optimization framework, given a specified inlet flow rate and recovery rate, ensures that key constraints are met, including minimum average permeation flux, maximum permeate salinity, and the maximum CPF across the entire system, which is crucial for mitigating fouling and scaling. The two-stage and batch configurations are employed in this work, which reduces inlet operating pressure compared with a standard one-stage SWRO, thereby alleviating flux and CPF on the lead elements. Further details on the optimization framework are presented in the materials and methods and in Notes S2 and S3.

Innovative membrane module design

It requires radically different module design for the next-generation UPM system operated at ultrafast water flux, such as more than 100 lmh. Migratory birds—such as pelicans[42] and geese[43]—fly in V-formations to save energy. Pelicans flying in vortex wakes achieve energy savings of 11.4%–14.0% by gliding longer or reducing wingbeat frequency.[42] Inspired by this mechanism, three types of spacers—cosine, parabolic, and V shaped—are studied, with their parametric geometric models shown in Figure S1. The ranges of geometric design parameters for cosine-, parabolic-, and V-shaped spacers are provided in Table S1. Furthermore, the proposed Bayesian-driven pattern search approach (Figure 2A) is employed for optimizing the membrane module to balance the

www.the-innovation.org

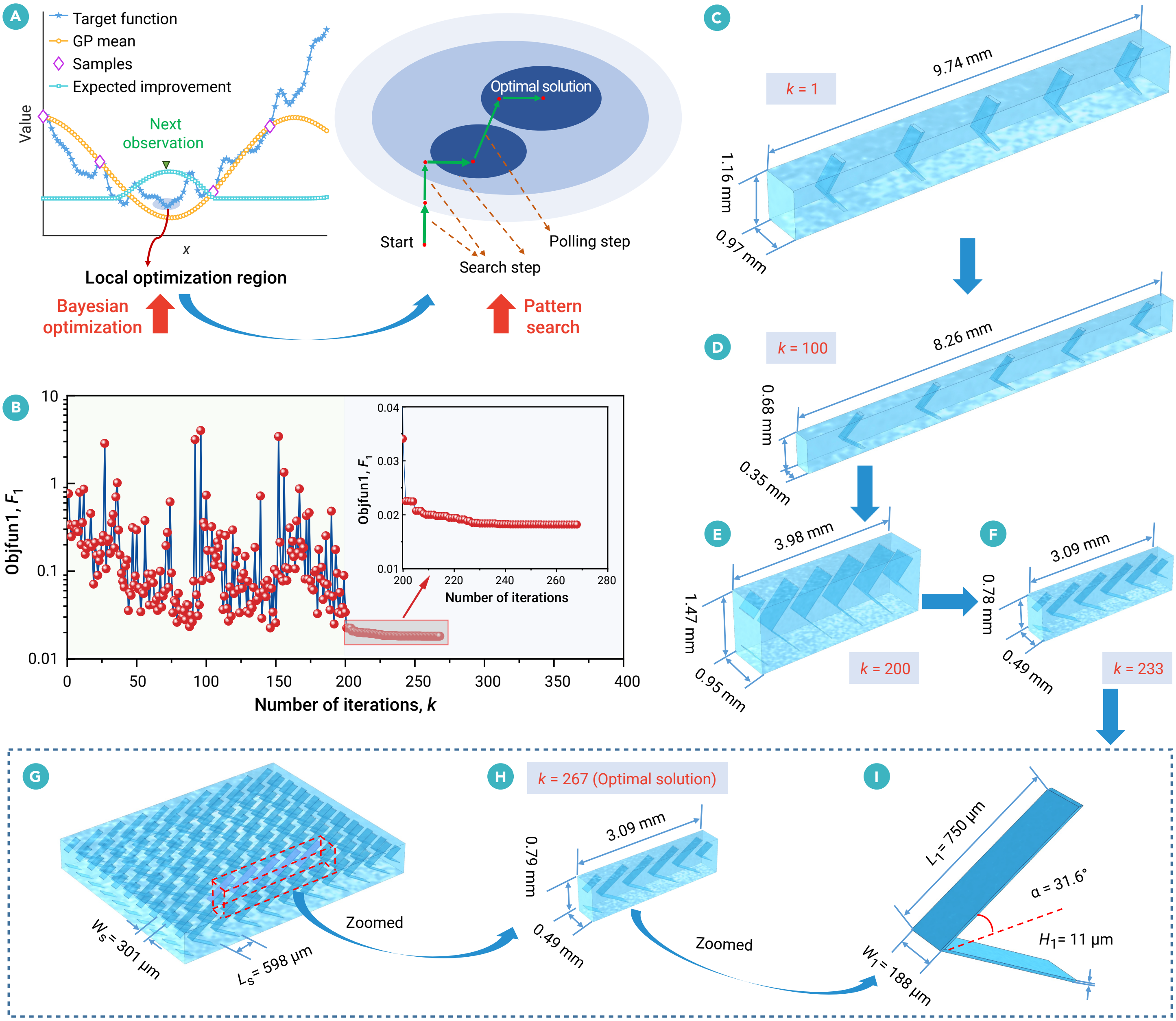

Figure 2. Bayesian-driven pattern search approach and its application (A) The schematic diagram of the hybrid optimization algorithm. (B–I) Convergence curve for optimizing the V-shaped spacer versus the number of iterations (k). The optimized solutions for (C) k = 1, (D) k = 100, (E) k = 200, and (F) k = 233. The optimal solution (k = 267) of (G) a small piece of spacer sheet (k = 267), (H) computational domain, and (I) a spacer cell. The optimal solution balances pressure drop and mass transfer with a trade-off parameter of β = 7 in the objective function (F_1) for this case. The F_1 is mathematically formulated as shown in Equation 2.

trade-off between mass transfer and flow resistance and maximize the benefit. Using this optimization approach, we calculate 30 optimized designs (Table S2) for three spacer shapes (V, cosine, and parabola shaped) across a range of trade-off parameters (β = 1, 2, ⋯, 10).

Taking the optimization process of the V-shaped spacer (β = 7), for example, the convergence curve (objective function F_1 versus number of iterations k) is shown in Figure 2B. Following Bayesian optimization, the identified optimal point is regarded as a local optimum region and subsequently refined using the pattern search algorithm. Several representative geometric structures for number of iterations, k = 1, 100, 200, and 233, are shown in Figures 2C–2F, respectively. The optimal solution (k = 267) for the feed spacer is shown in Figures 2G–2I. Using the Bayesian-driven pattern search algorithm, the optimized geometric parameters and the Sherwood numbers with respect to various Reynolds numbers, along with the repeatability test, are shown in Tables S3 and S4, respectively. Despite the stochastic nature of the Bayesian-based algorithm, the two independent calculations yielded highly consistent results, with relative deviations in the Sherwood numbers of less than 4%, demonstrating the optimization framework's replicability. Furthermore, we conduct sensitivity analysis of design parameter for the optimized objectives of $\bar{k}_m/\bar{k}_{m,0}$ and $\frac{\Delta P_c/L}{\Delta P_{c,0}/L_0}$. $\bar{k}_m/\bar{k}_{m,0}$ denotes the ratio of cell-averaged mass transfer coefficients for the optimized spacer ($\bar{k}_m$) versus the commercial spacer ($\bar{k}_{m,0}$) while $\frac{\Delta P_c/L}{\Delta P_{c,0}/L_0}$ is ratio of pressure drops per meter for the optimized spacer ($\Delta P_c/L$) versus the commercial spacer ($\Delta P_{c,0}/L_0$). Parameter sensitivity analysis highlights the effective trade-off between mass transfer and pressure drop for the optimized designs in this work (Table S5).

The mass transfer coefficients on the membrane wall of the three optimized spacers—V shaped (β = 7; Figure 3A), cosine shaped (β = 10; Figure 3B), and parabola shaped (β = 7; Figure 3C)—which offer the best trade-off between mass transfer and flow resistance, are significantly higher than those of the commercial spacer (Figure 3D). Accordingly, the 3D pressures of the optimized V-shaped (Figure 3E), cosine-shaped (Figure 3F), and parabola-shaped (Figure 3G) spacers are substantially lower than that of the commercial spacer (Figure 3H). This is primarily due to the generation of multi-vortex flow in the

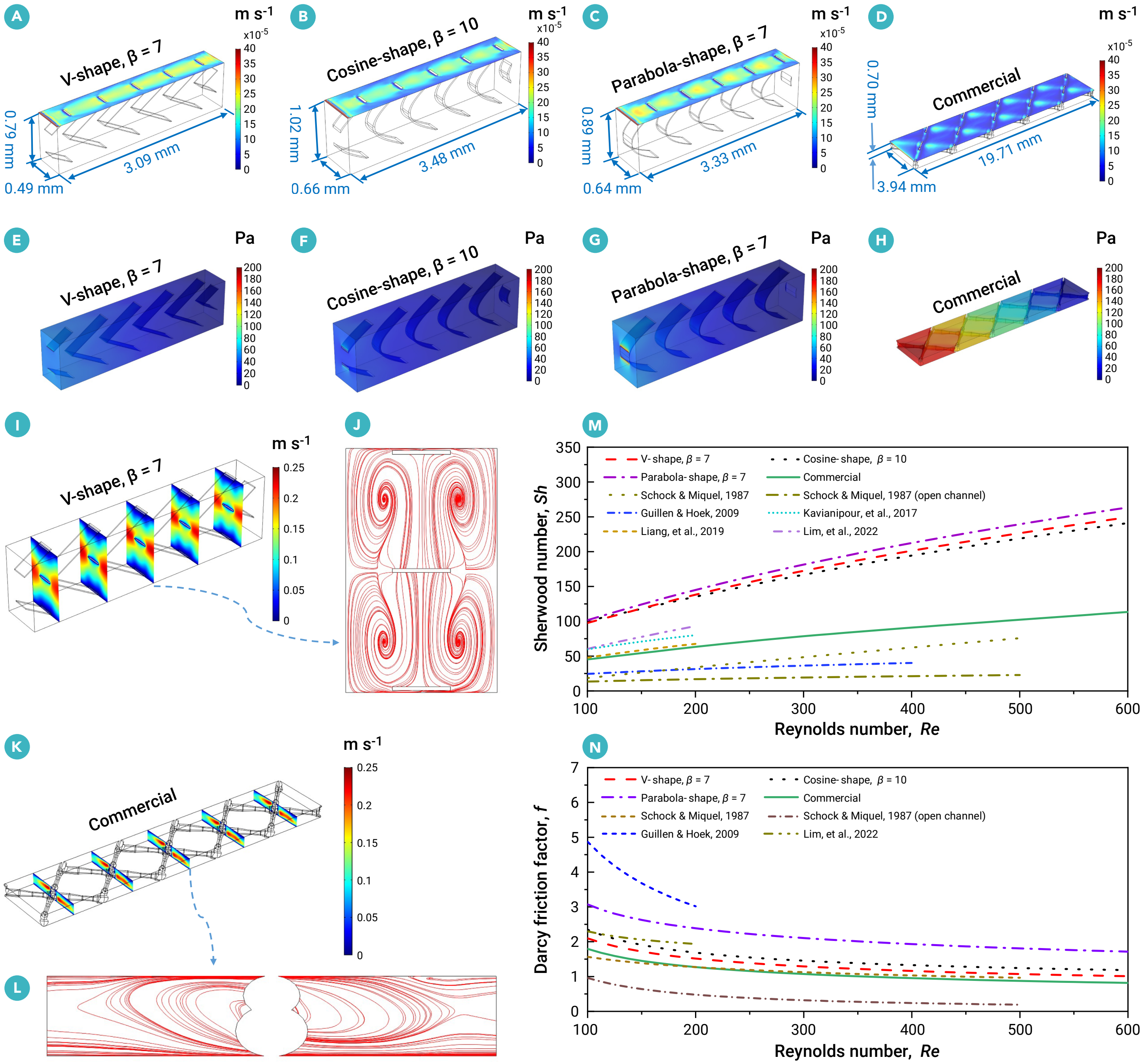

Figure 3. Hydrodynamics and mass transfer characteristics (A–L) Mass transfer coefficient distributions on membrane wall of optimized (A) V-shaped spacer (β = 7), (B) cosine-shaped spacer (β = 10), (C) parabola-shaped spacer (β = 7), and (D) commercial spacer. Pressure profiles of optimized (E) V-shaped spacer (β = 7), (F) cosine-shaped spacer (β = 10), (G) parabola-shaped spacer (β = 7), and (H) commercial spacer. (I) Velocity and (J) streamline distributions in the optimized V-shaped spacer (β = 7). (K) Velocity and (L) streamline distributions in the commercial spacer. The cross velocity is set to 0.1 m s^{-1} in (A–L). The Sherwood number and Darcy friction factor correlations for the optimized V-shaped spacer (β = 7), cosine-shaped spacer (β = 10), parabola-shaped spacer (β = 7), and the commercial spacer of this work and previously published correlations. (M) Sherwood number as a function of Reynolds number. (N) Darcy friction as a function of Reynolds number.

optimized channel, e.g., the V-shaped spacer (β = 7, Figures 3I and 3J), which results in a significant enhancement of mass transfer compared with the commercial spacer (Figures 3K and 3L). The flow pattern aligns with the theoretically optimal velocity, maximizing heat and mass transfer while accounting for viscous dissipation.[44,45] Furthermore, the relationships between the Sherwood number (Figure 3M) and the Darcy friction factor (Figure 3N) as functions of the Reynolds number are utilized to evaluate the fluid mechanics and transport performance for the optimized and commercial spacer in this work and the reported results in previous work.[46–51] For a Reynolds number of 100, the Sherwood numbers (*Sh* = 98, 100, and 102) for the optimized V-shaped spacer (β = 7), the cosine-shaped spacer (β = 10), and the parabola-shaped spacer (β = 7) are markedly higher—by 116%, 122%, and 125%, respectively—compared with that of the commercial spacer (*Sh* = 45). In contrast, the Darcy friction factors (*f* = 2.10, 2.34, and 3.07) for the V-shaped spacer (β = 7), the cosine-shaped spacer (β = 10), and the parabola-shaped spacer (β = 7) are elevated by 17%, 31%, and 71%, respectively, relative to the commercial spacer (*f* = 1.79). For a given crossflow velocity of 0.1 m s^{-1}, the cell-averaged mass transfer for the optimized V-shaped spacer (β = 7), the cosine-shaped spacer (β = 10), and the parabola-shaped spacer (β = 7) are 2.39, 2.27, and 1.99 times greater than that of the commercial spacer, with corresponding increases in pressure drop per meter of 54%, 76%, and 1%, respectively.

In our previous work,[30] the V-shaped spacer, inspired by the V-formation of birds, was proposed, achieving a 2.38-fold improvement in cell-averaged mass transfer over the commercial spacer, while accompanied by a pressure drop per meter up to four times higher. Therefore, the optimized results in this work represent a substantial advancement over our previous work.[30]

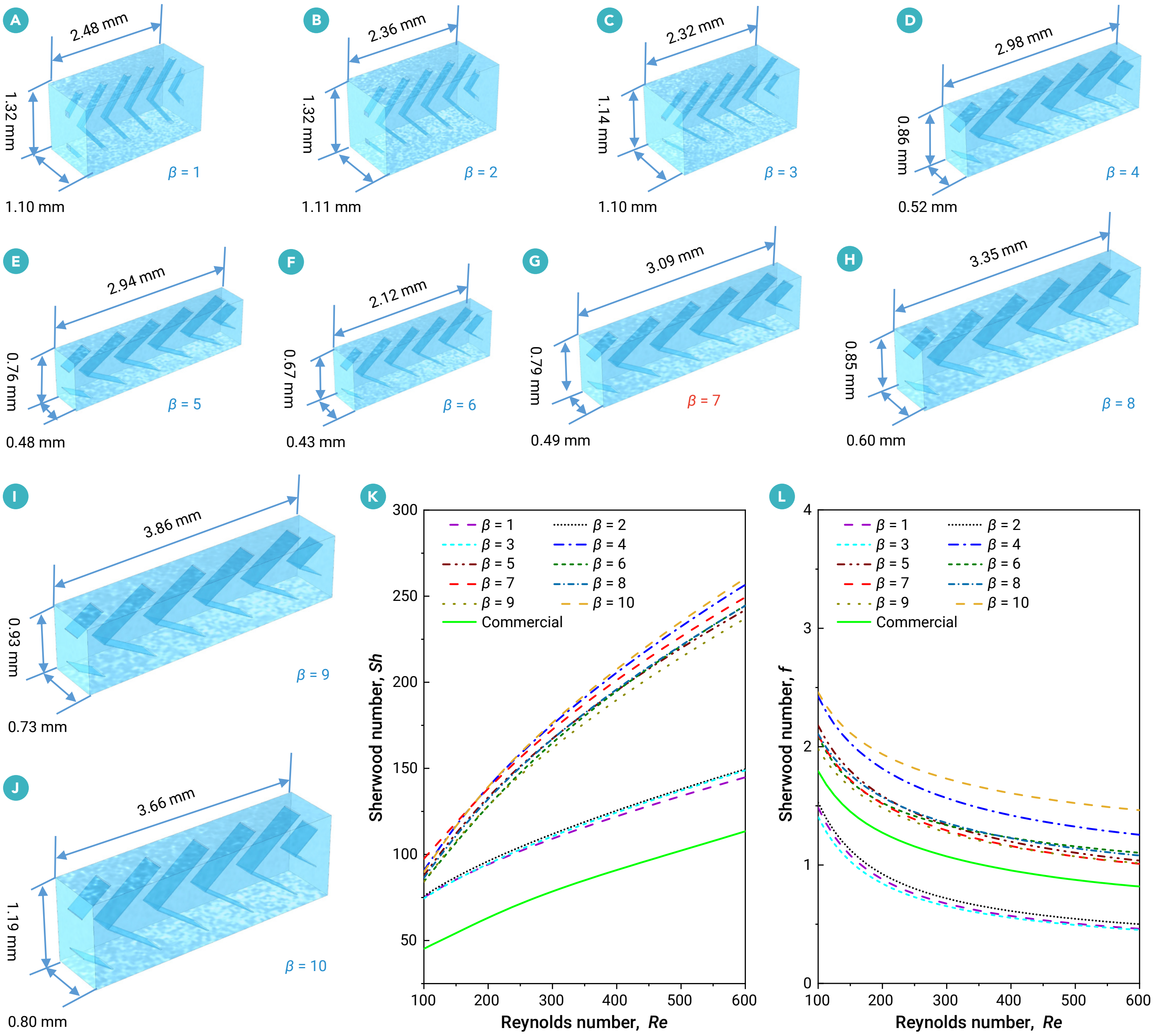

Figure 4. Optimized results for V-shaped spacers Optimized spacer geometries corresponding to different trade-off parameter values: (A) β = 1, (B) β = 2, (C) β = 3, (D) β = 4, (E) β = 5, (F) β = 6, (G) β = 7, (H) β = 8, (I) β = 9, and (J) β = 10. Additionally, (K) presents the Sherwood number as a function of Reynolds numbers for various β values, while (L) depicts the Darcy friction factor as a function of Reynolds numbers for different β values.

This improvement is largely attributed to the enhanced performance of the optimization algorithm, which enables the generation of superior solutions. Moreover, compared with the previously used genetic algorithm, the proposed method offers greater computational efficiency, making it feasible to explore a broader range of trade-off parameter values to achieve better solutions. Additionally, refinements in the objective function of this work may also contribute to the improved outcomes. Overall, the optimized V-shaped spacer (β = 7) outperforms the other two in balancing mass transfer and flow resistance. The results further validate that the V-shaped migration formation in birds reflects evolutionary intelligence, enhancing energy efficiency.[42] For a Reynolds number of 100, the Sherwood number (*Sh* = 98) for the optimized V-shaped spacer (β = 7) is 2.04, 1.62, and 5.26 times greater than that of the reported results, with corresponding variations in flow resistance of a 57% reduction,[48] a 9% decrease,[49] and increases of 34%,[46] respectively.

The detailed analysis process for exploring the optimal balance between mass transfer and flow resistance across the 30 optimized schemes is outlined as follows. Ratios of cell-averaged mass transfer coefficients and pressure drops per meter using optimized spacers (V, cosine, and parabola shaped) versus those of the commercial spacer are shown in Figure S2. As the cross-flow velocity increases, the mass transfer coefficient rises, albeit at the expense of a higher pressure drop. The mass transfer coefficient exhibits an initial increase followed by a decrease as the trade-off parameter β increases. This is primarily attributed to the incorporation of a penalty term associated with the pressure drop in the optimization objective function (Equation 2), designed to prevent excessive pressure loss in the optimized results. For a more comprehensive assessment of the optimized results, we further calculate the ratios of the mass transfer coefficients and the pressure drops per meter using the optimized spacers (V, cosine, and parabola shaped) versus those of the commercial spacer, with a cross velocity of 0.1 m s^{-1} (Figures S3A and S3B). The Sherwood number and Darcy friction factor are shown in Figures S3C and S3D, respectively, for a Reynolds number of 100. Obviously, the optimized V-shaped spacer (β = 7) achieves the best balance between the mass transfer (or Sherwood number) and pressure drop (or flow resistance). Moreover, the optimized spacer geometries are shown in Figures 4A–4J, respectively,

corresponding to different trade-off parameter values (for β = 1, 2, ⋯, 10). Accordingly, the calculated Sherwood numbers versus various Reynolds numbers are shown in Figures 4K and 4L. The additional enhancement in mass transfer for β = 7 is constrained compared with β = 4 and β = 10, whereas its flow resistance remains significantly lower than those of β = 4 and β = 10. Overall, the optimized V-shaped spacer with a trade-off parameter of β = 7 is recognized as the optimal configuration for further system design.

Optimal design of two-stage UPM SWRO system

Furthermore, the optimized membrane module with a V-shaped spacer (β = 7) is applied for designing two-stage UPM SWRO system (Figure 5A). The conditions for all cases in the two-stage SWRO include a feed salinity of 35,000 ppm, an inlet flow rate of 300 m^3 h^{-1}, a total recovery rate of 50%, a pump efficiency of 85%, and an energy recovery efficiency of 95%. The range of system design parameters for two-stage UPM SWRO is provided in Table S6. The optimized results for two-stage SWRO system are obtained with different maximum CPFs (CPF_{max} = 1.20, 1.25, and 1.30) constraints for various trade-off parameter values of c_m = 40, 100, 160, 220, 280, 340, and 400 \$ m^{-2}. The optimized results with a CPF_{max} = 1.25 constraint are preferred, as they result in a lower SEC under the same conditions for a required membrane area (Figure 5B) or an average water flux (Figure 5C) compared with those of CPF_{max} = 1.20, while effectively controlling membrane scaling and fouling.[49] Varying the trade-off parameter c_m could yield different optimized solutions tailored to specific requirements. As c_m increases, the required membrane area decreases (Figure 5B) or average water production increases (Figure 5C), while energy consumption correspondingly rises. This is primarily due to the reduced number of membrane modules, higher crossflow velocity, and increased energy consumption from flow resistance. The ultrafast water flux at the inlet—exceeding 250 lmh in the case of CPF_{max} = 1.25 (Figure 5D)—is often associated with the highest concentration polarization (Figure 5E). Therefore, it is crucial to control the CPF at the inlet within a reasonable range (no more than 1.25). To balance the trade-off between SEC and the required membrane area (or average water flux), the optimized results with c_m = 220 \$ m^{-2} (Figures 5F–5H) are recommended for further analysis.

Taking the case with a CPF_{max} = 1.25 constraint for example, the optimized SEC (1.78 kWh m^{-3}) and required membrane area (1,583 m^2) reduce 22% and 80%, respectively, compared with that of the commercial one-stage SWRO (2.30 kWh m^{-3} and 7,804 m^2). Accordingly, the optimized average water flux (95 lmh) achieves a 391% increase compared with the estimated value from the commercial design (19 lmh). Detailed results for the two-stage UPM and commercial designs with various c_m values (40, 220, and 400 \$ m^{-2}) are presented in Tables S7–S9, respectively. Furthermore, we assess the repeatability of the system design. The simulated results demonstrated optimized performance and high reproducibility, with deviations of less than 3% in SEC and average permeate salinity ($\overline{w}_p$), and below 7% in average water flux ($\overline{J}_W$), between two independent calculations (Table S10).

The two-stage design operates at lower pressures—43.2 bar in the first stage and 58.5 bar in the second stage—compared with 66.0 bar in the commercial one-stage SWRO, resulting in a lower SEC (Figure 6A). Although the permeate salinity for the UPM systems will also increase compared with commercial membranes (Figure 6B), the average permeate salinity in all cases of this work remains below 500 ppm, meeting potable water standards.[52] Under ultrafast water flux conditions, the mass transfer at the system inlet using the optimized membrane module is 3.57 times greater than that of the commercial design, accompanied by an approximately 4-fold increase in pressure drop per meter (Figures 6C and 6D). However, predictive models from a previous study[20] indicate that achieving a 3.57-fold increase in mass transfer by merely increasing crossflow velocity in the commercial design would cause an impractical 97-fold rise in pressure drop. Hence, optimizing the synergistic relationship between velocity and concentration fields in the spacer-filled channel through feed spacer design offers a more effective strategy for enhancing mass transfer while balancing an acceptable pressure loss penalty, rather than simply increasing crossflow velocity. This further validates the field synergy theory proposed for enhancing heat transfer.[53,54] The designed spacers will be further fabricated using advanced 3D printing technologies to assess their performance in hydrodynamics, mass transfer, and mechanical stability.

Optimal design of UPM batch SWRO systems

Batch RO has attracted considerable attention due to its internal staging feature, which is divided into three stages: production, flushing, and refill (Figure 7A). A comparison of normalized SEC breakdowns for batch RO, including factors such as thermodynamics, design flux, flow resistance, concentration polarization, salt retention, and pump inefficiency, is presented in Table S11.

The SEC for batch RO is evaluated across various membrane permeabilities under both normal and ultrafast water flux conditions, using optimized and commercial spacers, respectively (Figure 7B). Under normal average water flux (19 lmh), SEC gradually decreases as membrane permeability increases from 1 to 4 lmh bar^{-1}. When membrane permeability exceeds 4 lmh bar^{-1}, further reductions in SEC become marginal. This is consistent with reported conclusions[24] that the use of advanced membrane materials has a limited impact on further improving energy efficiency in RO and other desalination technologies. However, under ultrafast average water flux (>220 lmh), SEC continues to significantly decrease until membrane permeability exceeds 30 lmh bar^{-1} (Figure 7B). Thus, optimizing membrane permeability to around 30 lmh bar^{-1}, coupled with ideal salt selectivity, is essential for enhancing energy efficiency in the context of ultrafast water flux. Optimization of the membrane module offers greater potential for reducing SEC under ultrafast water flux compared with normal water flux, while also mitigating CPF and the risks of membrane scaling and fouling. This is consistent with the conclusions reported in previous literature[20] that the performance of the UPM is limited by the fluid mechanics and mass transfer of the membrane module, defining the upper bounds.

We further evaluate the potential through membrane permeability, membrane module design, and the use of batch RO, with the trade-off between SEC and average water flux shown in Figure 7C. For comparison, membrane permeability is fixed at 34.9 lmh bar^{-1}, consistent with the optimized configuration in the two-stage design (c_m = 220 \$ m^{-2}). The optimized V-shaped spacer (β = 7) significantly reduces SEC compared with the commercial spacer, while effectively maintaining CPF below 1.25. If using the commercial spacer, the CPFs for all cases in batch RO are more than 1.25. When the ultrafast average water flux is increased to 224 lmh, the CPF increases to an impractical value of 1.42, further intensifying membrane fouling. Accordingly, the SEC is 2.30 kWh m^{-3}, which does not provide energy-saving benefits compared with the commercial one-stage SWRO. Here, we propose an optimized design scheme incorporating an UPM, optimized membrane module, and batch RO (Figure 7D). The required membrane area (679 m^2) and SEC (2.04 kWh m^{-3}) are reduced by 91% and 11%, respectively. The average water flux reaches 221 lmh, which is 11.5 times higher than that of the commercial SWRO (19 lmh). The maximum CPF is 1.21, which falls within a reasonable range of less than 1.25.[49] Comparison of SEC breakdowns (Figures 7E and 7F) reveals that higher concentration polarization is the primary factor contributing to the increase in SEC (from 2.04 to 2.30 kWh m^{-3}) using the commercial spacer relative to that using the optimized spacer. If ultrahigh water flux is not a priority, the SEC can further reduce by 1.68 kWh m^{-3} with an average water flux of 95 lmh. It is twice that of the optimized two-stage design (47 lmh), with an equivalent SEC of 1.67 kWh m^{-3}. This is mainly due to the uniform flux in batch RO, which allows operation at a higher average flux under the same maximum concentration polarization constraint, compared with the two-stage design. Detailed results using the optimized and commercial spacers in UPM batch SWRO desalination are presented in Tables S12 and S13, respectively.

DISCUSSION

In recent years, UPM materials have received widespread attention and extensive research. However, the mere discovery of new materials is insufficient for the realization of cost-effective water treatment technologies. The limitations in fluid dynamics and mass transfer of conventional membrane module may define the upper bounds of membrane performance.[20] It is imperative to design and develop advanced membrane materials within the context of integrated unit processes and the entire water treatment system.[55] By combining process system optimization models with techno-economic analysis and life cycle assessments, it becomes possible to redefine the theoretical upper limits of membrane performance improvements while simultaneously exploring the optimal balance between capital and operational costs.[55] Herein, we propose

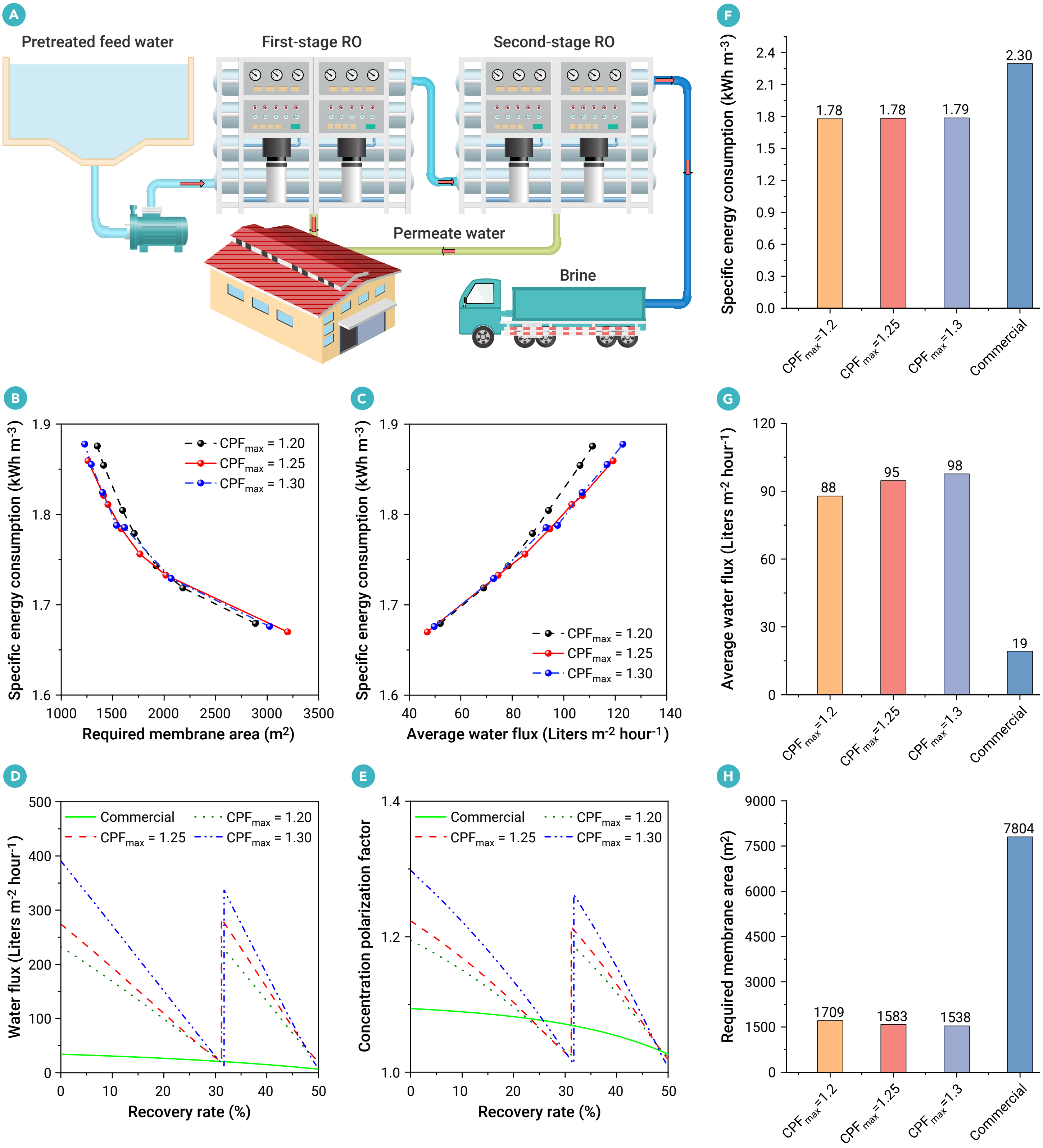

Figure 5. Optimal design of two-stage ultrapermeable reverse osmosis systems (A) Process flow diagram for two-stage reverse osmosis (RO). (B) Pareto front optimization between specific energy consumption (kWh m^{-3}) and required membrane area (m^2). (C) Pareto front optimization between specific energy consumption (kWh m^{-3}) and average water flux (L m^{-2} h^{-1}). (D) Water flux (L m^{-2} h^{-1}) versus recovery rate (%). (E) Concentration polarization factor versus recovery rate (%). (F–H) (F) Specific energy consumption, (G) average water flux (L m^{-2} h^{-1}), (H) required membrane area (m^2) for optimized two-stage ROs and commercial one-stage RO. The optimized results for two-stage RO system are obtained with respect to various maximum concentration polarization factor (CPF_{max} = 1.20, 1.25, and 1.30) constraints. The conditions for all cases in the two-stage RO include a feed salinity of 35,000 ppm, an inlet flow rate of 300 m^3 h^{-1}, a total recovery rate of 50%, a pump efficiency of 85%, and an energy recovery efficiency of 95%.

a multiscale optimization framework coupling the optimal selection of membrane permeability, membrane module optimization and system design (with two-stage and batch configurations) in SWRO desalination. A Bayesian-driven pattern search approach is developed for feed spacer design.

This work presents a transformative advance in membrane desalination technology by fundamentally redefining the long-standing trade-off between energy efficiency and water production efficiency (or average water flux). Through the synergistic integration of bio-inspired UPM module with state-of-the-art

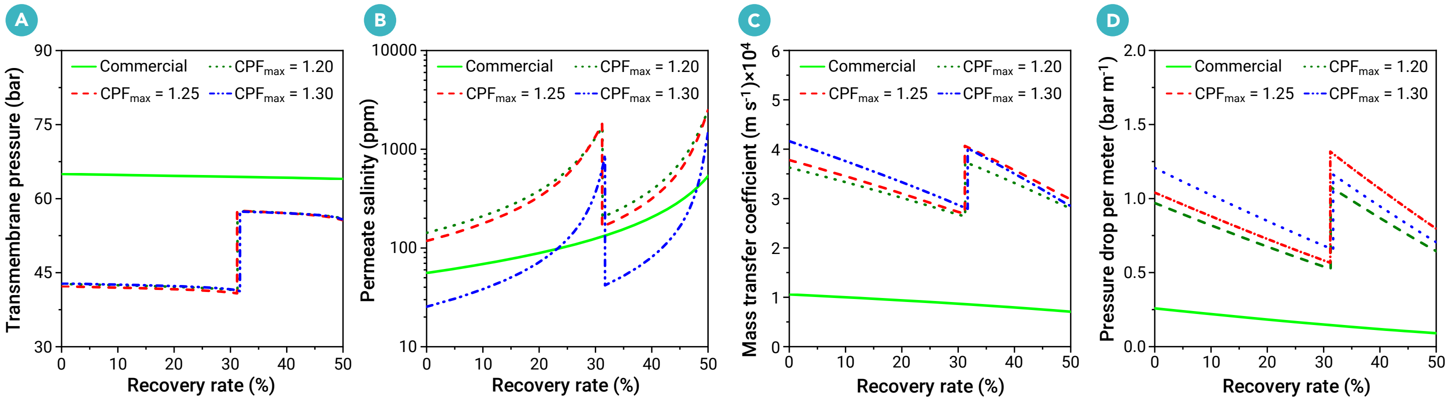

Figure 6. System performance analysis (A) Transmembrane pressure (bar), (B) permeate salinity (parts per million, ppm), (C) mass transfer coefficient (m s^{-1}), and (D) pressure drop per meter (bar m^{-1}) with respect to various recovery rate (%). c_m is 220 \$ m^{-2} which is the trade-off parameter in the objective function (F_2) to balance SEC (kWh m^{-3}) and the required total membrane area (m^2). The F_2 is mathematically formulated as shown in Equation 5. The optimized results for two-stage RO system are obtained with respect to various maximum concentration polarization factor (CPF$_{max}$ = 1.20, 1.25, and 1.30) constraints. The conditions for all cases in the two-stage RO include a feed salinity of 35,000 ppm, a total recovery rate of 50%, a pump efficiency of 85%, and an energy recovery efficiency of 95%.

batch RO, we demonstrate unprecedented performance—achieving a SEC of just 1.68 kWh m^{-3} while delivering an average water flux of 95 lmh. This represents a 33%–58% reduction in energy demand and a 5-fold improvement in water flux compared with modern plants for seawater RO desalination (feed salinity of 35,000 ppm, recovery rate of 50%). The concentration polarization is maintained within a reasonable range (no more than 1.25), reducing the risks of membrane scaling and fouling. The desalination energy efficiency, achieved through UPMs, optimized module design, and batch RO, shows significant improvement under ultrafast average water flux. However, when membrane permeability exceeds 30 lmh bar^{-1}, additional reductions have minimal impact, unless future advancements yield membrane modules or processes with superior fluid dynamics and mass transfer characteristics relative to the present

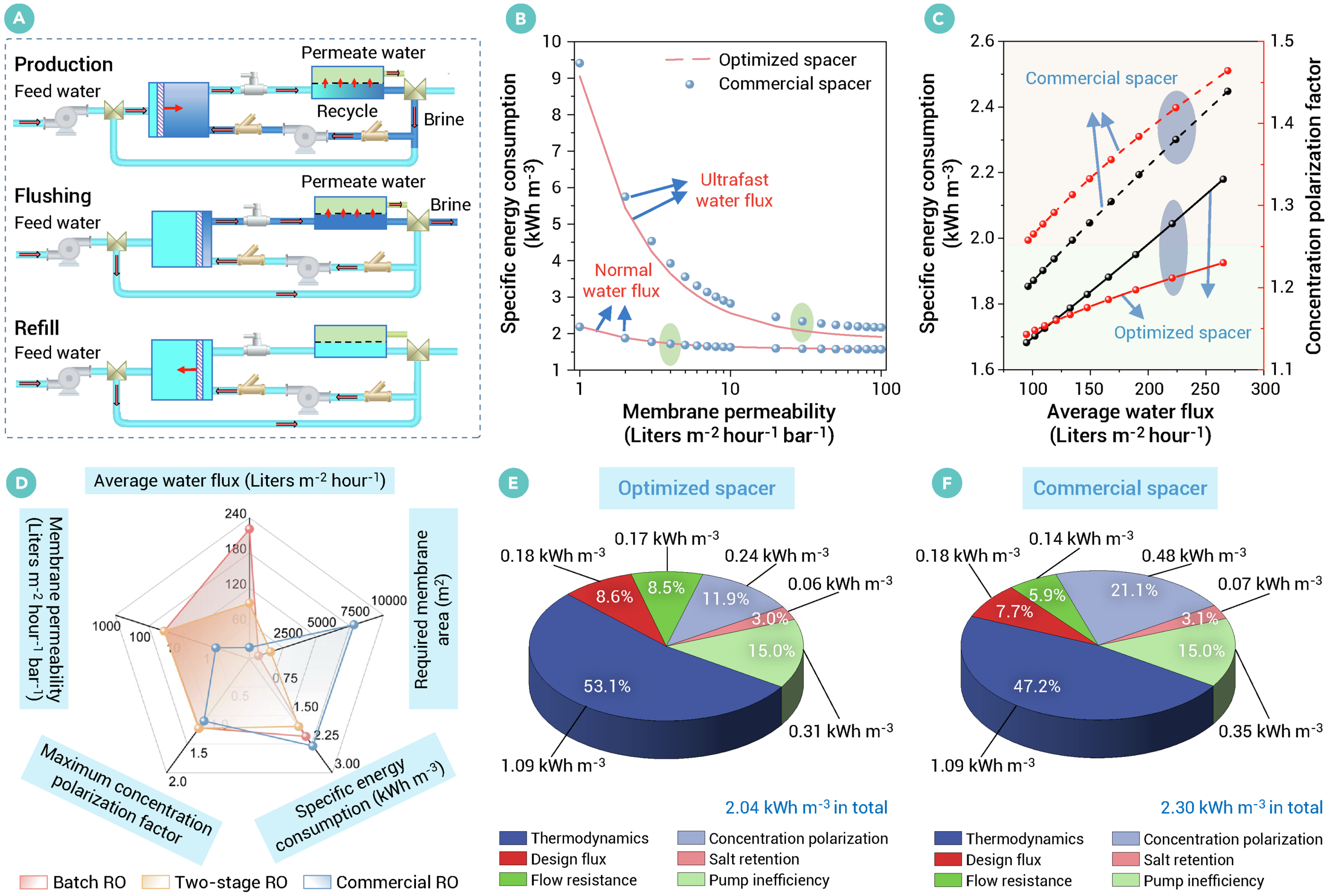

Figure 7. Optimal design of ultrapermeable batch reverse osmosis systems (A) Process flow diagram for batch RO. (B) SEC (kWh m^{-3}) versus membrane permeability (L m^{-2} h^{-1} bar^{-1}, lmh bar^{-1}) for batch RO. The ultrafast average water flux: 221 lmh for the optimized V-shaped spacer and 224 lmh for the commercial spacer. Normal average water flux: 19 lmh. (C) SEC (kWh m^{-3}) and concentration polarization factor versus average water flux (lmh) using an optimized V-shaped spacer and commercial spacer for batch RO. (D–F) (D) SEC (kWh m^{-3}), average water flux (lmh), required membrane area (m^2), maximum concentration polarization factor and membrane permeability (lmh bar^{-1}) for optimized batch and two-stage ROs and commercial RO. Comparison of SEC breakdowns using (E) optimized V-shaped spacer and (F) commercial spacer for batch RO. The conditions for all cases in the batch RO include a feed salinity of 35,000 ppm, an inlet flow rate of 300 m^3 h^{-1}, a total recovery rate of 50%, and a pump efficiency of 85%.

www.the-innovation.org

work. This finding is crucial for guiding the future direction of membrane material development and improving energy efficiency of desalination technologies. These breakthroughs redefine desalination technology, demonstrating how engineered UPMs can simultaneously boost energy efficiency and water production efficiency. Our findings establish design principles that enable next-generation desalination plants and open new possibilities for zero-liquid-discharge systems[5] and other advanced water treatment applications.

Our prior work[56] introduced a hybrid model for RO desalination, integrating 3D CFD with a one-dimensional system-scale approach. Simulations using this framework yielded transmembrane pressure predictions within 5% of industrial data and closely matched the total flow rate, as benchmarked against operational data[57] from the Chino Desalter I facility, California. Nevertheless, the multiscale design scheme, e.g., structural integrity for the designed innovative membrane module (or feed spacer) under high-pressure operation still requires experimental validation in future work. The energy recovery device operates within the two-stage system at relatively constant pressure. Progressive membrane fouling during operation reduces water permeability, necessitating elevated operational pressure to maintain constant flux. This pressure increase may perturb energy recovery device efficiency; however, the effects of pressure fluctuations lie beyond this work's scope and warrant future investigation. Furthermore, the assumption of identical residence time distributions across modules is limited by potential fouling-induced variability. The implementation of the optimized system faces practical challenges: (1) the scalable production of UPMs that balance high salt rejection and mechanical strength with low cost; (2) the scalable manufacturing of optimized feed spacers, despite the promise of low-cost methods such as 3D printing; and (3) the development of a closed-loop control system to maintain constant permeate flux via real-time pressure adjustments.

RESOURCE AVAILABILITY

Materials availability

This study did not generate new unique materials/reagents.

Data and code availability

Data and code are available from the corresponding author upon reasonable request.

FUNDING AND ACKNOWLEDGMENTS

Y.H. acknowledges support provided by the Key Area Research and Development Program of Guangdong Province, China (2021B0101190003). J.L. thanks support provided by the Opening Project of Guangdong Province Key Laboratory of Computational Science at the Sun Yat-sen University (2024014). J.W. thanks support provided by the Suzhou Planning Project of Science and Technology (2023ss03) and the Key Laboratory of General Artificial Intelligence and Large Models in Provincial Universities, Soochow University.

AUTHOR CONTRIBUTIONS

J.L. and Y.H. designed the research. J.L. and X.L. provided the data, models and computational methods, and performed the research. J.L., X.L., J.W., and Y.H. analyzed the data and wrote the paper.

DECLARATION OF INTERESTS

The authors declare no competing interests.

SUPPLEMENTAL INFORMATION

It can be found online at https://doi.org/10.1016/j.xinn.2026.101262.

REFERENCES

1. Shannon, M.A., Bohn, P.W., Elimelech, M. et al. (2008). Science and technology for water purification in the coming decades. *Nature* **452**:301–310. DOI:10.1038/nature06599
2. Eke, J., Yusuf, A., Giwa, A. et al. (2020). The global status of desalination: An assessment of current desalination technologies, plants and capacity. *Desalination* **495**:114633. DOI:10.1016/j.desal.2020.114633
3. Qasim, M., Badrelzaman, M., Darwish, N.N. et al. (2019). Reverse osmosis desalination: A state-of-the-art review. *Desalination* **459**:59–104. DOI:10.1016/j.desal.2019.02.008
4. Alnajdi, S., Naderi Beni, A., Alsaati, A.A. et al. (2024). Practical minimum energy use of seawater reverse osmosis. *Joule* **8**:3088–3105. DOI:10.1016/j.joule.2024.08.005
5. O'Connell, M.G., Rajendran, N., Elimelech, M. et al. (2024). Analysis of energy, water, land and cost implications of zero and minimal liquid discharge desalination technologies. *Nat. Water* **2**:1116–1127. DOI:10.1038/s44221-024-00327-1
6. Wang, Z., Ma, C., Xu, C. et al. (2021). Graphene oxide nanofiltration membranes for desalination under realistic conditions. *Nat. Sustain.* **4**:402–408. DOI:10.1038/s41893-020-00674-3
7. Zhang, W.H., Yin, M.J., Zhao, Q. et al. (2021). Graphene oxide membranes with stable porous structure for ultrafast water transport. *Nat. Nanotechnol.* **16**:337–343. DOI:10.1038/s41565-020-00833-9
8. Tunuguntla, R.H., Henley, R.Y., Yao, Y.C. et al. (2017). Enhanced water permeability and tunable ion selectivity in subnanometer carbon nanotube porins. *Science* **357**:792–796. DOI:10.1126/science.aan2438
9. Yang, Y., Yang, X., Liang, L. et al. (2019). Large-area graphene-nanomesh/carbon-nanotube hybrid membranes for ionic and molecular nanofiltration. *Science* **364**:1057–1062. DOI:10.1126/science.aau5321
10. Li, Y., Li, Z., Aydin, F. et al. (2020). Water-ion permselectivity of narrow-diameter carbon nanotubes. *Sci. Adv.* **6**:eaba9966. DOI:10.1126/sciadv.aba9966
11. Song, Q., Lin, Y., Zhou, S. et al. (2023). Highly permeable nanofilms with asymmetric multilayered structure engineered via amine-decorated interlayered interfacial polymerization. *J. Memb. Sci.* **670**:121377. DOI:10.1016/j.memsci.2023.121377
12. Qin, Y., Qi, P., Hao, S. et al. (2025). Methylation of reverse osmosis membrane for superior anti-fouling performance via blocking carboxyl groups in polyamide. *Nat. Water* **3**:110–121. DOI:10.1038/s44221-024-00371-x
13. Chowdhury, M.R., Steffes, J., Huey, B.D. et al. (2018). 3D printed polyamide membranes for desalination. *Science* **361**:682–686. DOI:10.1126/science.aar2122
14. Kocsis, I., Sun, Z., Legrand, Y.M. et al. (2018). Artificial water channels—deconvolution of natural Aquaporins through synthetic design. *npj Clean Water* **1**:13. DOI:10.1038/s41545-018-0013-y
15. Fuwad, A., Ryu, H., Han, E.D. et al. (2024). Highly permeable and shelf-stable aquaporin biomimetic membrane based on an anodic aluminum oxide substrate. *npj Clean Water* **7**:11. DOI:10.1038/s41545-024-00301-0
16. Lee, C.S., Kim, I., Jang, J.W. et al. (2021). Aquaporin-Incorporated Graphene-Oxide Membrane for Pressurized Desalination with Superior Integrity Enabled by Molecular Recognition. *Adv. Sci.* **8**:e2101882. DOI:10.1002/advs.202101882
17. Itoh, Y., Chen, S., Hirahara, R. et al. (2022). Ultrafast water permeation through nanochannels with a densely fluorous interior surface. *Science* **376**:738–743. DOI:10.1126/science.abd0966
18. Di Vincenzo, M., Tiraferri, A., Musteata, V.E. et al. (2021). Biomimetic artificial water channel membranes for enhanced desalination. *Nat. Nanotechnol.* **16**:190–196. DOI:10.1038/s41565-020-00796-x
19. Yao, Y., Zhang, P., Sun, F. et al. (2024). More resilient polyester membranes for high-performance reverse osmosis desalination. *Science* **384**:333–338. DOI:10.1126/science.adk0632
20. Fane, A.G., Wang, R. and Hu, M.X. (2015). Synthetic membranes for water purification: status and future. *Angew. Chem. Int. Ed. Engl.* **54**:3368–3386. DOI:10.1002/anie.201409783
21. Liu, J., Iranshahi, A., Lou, Y. et al. (2013). Static mixing spacers for spiral wound modules. *J. Memb. Sci.* **442**:140–148. DOI:10.1016/j.memsci.2013.03.063
22. Chong, Y.K., Liang, Y.Y. and Weihs, G.A.F. (2023). Validation and characterisation of mass transfer of 3D-CFD model for twisted feed spacer. *Desalination* **554**:116516. DOI:10.1016/j.desal.2023.116516
23. Luo, J., Li, M., Hoek, E.M.V. et al. (2023). Supercomputing and machine learning-aided optimal design of high permeability seawater reverse osmosis membrane systems. *Sci. Bull.* **68**:397–407. DOI:10.1016/j.scib.2023.01.039
24. Patel, S.K., Ritt, C.L., Deshmukh, A. et al. (2020). The relative insignificance of advanced materials in enhancing the energy efficiency of desalination technologies. *Energy Environ. Sci.* **13**:1694–1710. DOI:10.1039/d0ee00341g
25. Li, M. (2021). A spatiotemporal model for dynamic RO simulations. *Desalination* **516**:115229. DOI:10.1016/j.desal.2021.115229
26. Hosseinipour, E., Park, K., Burlace, L. et al. (2022). A free-piston batch reverse osmosis (RO) system for brackish water desalination: Experimental study and model validation. *Desalination* **527**:115524. DOI:10.1016/j.desal.2021.115524
27. Li, M., Heng, Y. and Luo, J. (2020). Batch reverse osmosis: a new research direction in water desalination. *Sci. Bull.* **65**:1705–1708. DOI:10.1016/j.scib.2020.05.032
28. Mo, Z., Li, D. and She, Q. (2022). Semi-closed reverse osmosis (SCRO): A concise, flexible, and energy-efficient desalination process. *Desalination* **544**:116147. DOI:10.1016/j.desal.2022.116147
29. Li, M. (2023). An improved Closed-Circuit RO (CCRO) system: Design and cyclic simulation. *Desalination* **554**:116519. DOI:10.1016/j.desal.2023.116519
30. Luo, J., Li, M. and Heng, Y. (2024). Bio-inspired design of next-generation ultrapermeable membrane systems. *npj Clean Water* **7**:4. DOI:10.1038/s41545-024-00297-7
31. Shahriari, B., Swersky, K., Wang, Z. et al. (2016). Taking the human out of the looop: A review of Bayesian optimization. *Proc. IEEE* **104**:148–175. DOI:10.1109/jproc.2015.2494218
32. Savage, T., Basha, N., McDonough, J. et al. (2024). Machine learning-assisted discovery of flow reactor designs. *Nat. Chem. Eng.* **1**:522–531. DOI:10.1038/s44286-024-00099-1
33. Wang, C., Heng, Y., Luo, J. et al. (2024). A fast Bayesian parallel solution framework for large-scale parameter estimation of 3D inverse heat transfer problems. *Int. Commun. Heat Mass Transf.* **155**:107409. DOI:10.1016/j.icheatmasstransfer.2024.107409
34. Lee, W., Sun, Y. and Lu, S. (2020). Hierarchical sparse observation models and informative prior for Bayesian inference of spatially varying parameters. *J. Comput. Phys.* **422**:109768. DOI:10.1016/j.jcp.2020.109768
35. Hooke, R. and Jeeves, T.A. (1961). "Direct search" solution of numerical and statistical problems. *J. ACM* **8**:212–229. DOI:10.1145/321062.321069

36. Kolda, T.G., Lewis, R.M. and Torczon, V. (2003). Optimization by direct search: New perspectives on some classical and modern methods. *SIAM Rev.* **45**:385–482. DOI:10.1137/s003614450242889
37. Toh, K.Y., Liang, Y.Y., Lau, W.J. et al. (2020). The techno-economic case for coupling advanced spacers to high-permeance RO membranes for desalination. *Desalination* **491**:114534. DOI:10.1016/j.desal.2020.114534
38. Chipperfield, A.J., Fleming, P.J., Pohlheim, H. et al. (1994). A genetic algorithm toolbox for MATLAB. *Int. Conf. Syst. Eng.* **6**:200–207
39. Li, M. (2021). Residence time distribution in RO channel. *Desalination* **506**:115000. DOI:10.1016/j.desal.2021.115000
40. Geraldes, V. and Afonso, M.D. (2006). Generalized mass-transfer correction factor for nanofiltration and reverse osmosis. *AIChE J.* **52**:3353–3362. DOI:10.1002/aic.10968
41. Elimelech, M. and Phillip, W.A. (2011). The future of seawater desalination: energy, technology, and the environment. *Science* **333**:712–717. DOI:10.1126/science.1200488
42. Weimerskirch, H., Martin, J., Clerquin, Y. et al. (2001). Energy saving in flight formation. *Nature* **413**:697–698. DOI:10.1038/35099670
43. May, R.M. (1979). Flight formations in geese and other birds. *Nature* **282**:778–780. DOI:10.1038/282778a0
44. Chen, Q. and Meng, J.A. (2008). Field synergy analysis and optimization of the convective mass transfer in photocatalytic oxidation reactors. *Int. J. Heat Mass Transf.* **51**:2863–2870. DOI:10.1016/j.ijheatmasstransfer.2007.09.024
45. Meng, J.A., Liang, X.G. and Li, Z.X. (2005). Field synergy optimization and enhanced heat transfer by multi-longitudinal vortexes flow in tube. *Int. J. Heat Mass Transf.* **48**:3331–3337. DOI:10.1016/j.ijheatmasstransfer.2005.02.035
46. Schock, G. and Miquel, A. (1987). Mass transfer and pressure loss in spiral wound modules. *Desalination* **64**:339–352. DOI:10.1016/0011-9164(87)90107-X
47. Guillen, G. and Hoek, E.M.V. (2009). Modeling the impacts of feed spacer geometry on reverse osmosis and nanofiltration processes. *Chem. Eng. J.* **149**:221–231. DOI:10.1016/j.cej.2008.10.030
48. Liang, Y.Y., Toh, K.Y. and Fimbres Weihs, G.A. (2019). 3D CFD study of the effect of multi-layer spacers on membrane performance under steady flow. *J. Memb. Sci.* **580**:256–267. DOI:10.1016/j.memsci.2019.02.015
49. Lim, Y.J., Ma, Y., Chew, J.W. et al. (2022). Assessing the potential of highly permeable reverse osmosis membranes for desalination: Specific energy and footprint analysis. *Desalination* **533**:115771. DOI:10.1016/j.desal.2022.115771
50. Kavianipour, O., Ingram, G.D. and Vuthaluru, H.B. (2017). Investigation into the effectiveness of feed spacer configurations for reverse osmosis membrane modules using Computational Fluid Dynamics. *J. Memb. Sci.* **526**:156–171. DOI:10.1016/j.memsci.2016.12.034
51. Li, M., Bui, T. and Chao, S. (2016). Three-dimensional CFD analysis of hydrodynamics and concentration polarization in an industrial RO feed channel. *Desalination* **397**:194–204. DOI:10.1016/j.desal.2016.07.005
52. Johnson, J. and Busch, M. (2010). Engineering aspects of reverse osmosis module design. *Desalin. Water Treat.* **15**:236–248. DOI:10.5004/dwt.2010.1756
53. Guo, Z.Y., Li, D.Y. and Wang, B.X. (1998). A novel concept for convective heat transfer enhancement. *Int. J. Heat Mass Transf.* **41**:2221–2225. DOI:10.1016/S0017-9310(97)00272-X
54. Guo, Z.Y., Tao, W.Q. and Shah, R.K. (2005). The field synergy (coordination) principle and its applications in enhancing single phase convective heat transfer. *Int. J. Heat Mass Transf.* **48**:1797–1807. DOI:10.1016/j.ijheatmasstransfer.2004.11.007
55. McCutcheon, J.R. and Mauter, M.S. (2023). Fixing the desalination membrane pipeline. *Science* **380**:242–244. DOI:10.1126/science.ade5313
56. Luo, J., Li, M. and Heng, Y. (2020). A hybrid modeling approach for optimal design of non-woven membrane channels in brackish water reverse osmosis process with high-throughput computation. *Desalination* **489**:114463. DOI:10.1016/j.desal.2020.114463
57. Li, M. and Noh, B. (2012). Validation of model-based optimization of brackish water reverse osmosis (BWRO) plant operation. *Desalination* **304**:20–24. DOI:10.1016/j.desal.2012.07.029

The Innovation, Volume 7

Supplemental Information

Intelligent design breaks the trade-off between energy efficiency and water flux in ultrafast seawater desalination

Jiu Luo, Xing Liu, Jin Wang, and Yi Heng

Supplementary Information for

Intelligent design breaks the trade-off between energy efficiency and water flux in ultrafast seawater desalination

Jiu Luo[1, 2], Xing Liu[1], Jin Wang[1, 2], Yi Heng[3*]

[1] School of Future Science and Engineering, Soochow University, Suzhou, 215222, China

[2] Key Laboratory of General Artificial Intelligence and Large Models in Provincial Universities, Soochow University, Suzhou, 215222, China

[3] School of Computer Science and Engineering, Sun Yat-sen University, Guangzhou, 510006, China

* Correspondence: hengyi@mail.sysu.edu.cn (Y. H.)

This PDF file includes:

Supplementary notes S1-S3
Figures S1-S3
Tables S1-S13
Supplementary references

Supplementary Note S1

1.1. Bayesian algorithm

During the global exploration phase, traditional intelligent optimization techniques, such as particle swarm optimization and genetic algorithm, typically require a large number of iterative evaluations to progressively approximate the global optimum. These methods lack an efficient mechanism to prioritize the exploration of potentially optimal regions, relying instead on randomness and natural selection, which often leads to redundant function evaluations. Given the black-box nature of the problem, Bayesian optimization presents a more suitable alternative. By leveraging probabilistic models, Bayesian optimization systematically approximates the objective function, enabling more informed and efficient exploration.

In this work, we employ the widely used Gaussian process model, defined as $F(x) \sim GP(\mu(x), \sigma^2(x))$, where $\mu(x)$ represents the predicted mean and $\sigma(x)$ quantifies the prediction uncertainty. Beyond estimating the objective value at each point, the Gaussian process also provides an uncertainty measure, enabling Bayesian optimization to strategically select sampling points that maximize potential improvement while reducing uncertainty. For complex and computationally expensive objective functions, this probabilistic modeling approach allows Bayesian optimization to efficiently identify the optimal solution while maintaining relatively low computational costs.

Among common acquisition functions—such as Probability of Improvement (PI)[1], Expected Improvement (EI)[2], and Lower Confidence Bound (LCB)[3]—EI is often preferred, and is expressed as:

$$EI(x) = (\mu(x) - f(x+))\Phi\left(\frac{(\mu(x) - f(x+))}{\sigma(x)}\right) + \sigma(x)\varphi\left(\frac{(\mu(x) - f(x+))}{\sigma(x)}\right) \quad (1)$$

where $\mu(x)$ is the mean of the Gaussian process at point x, $f(x+)$ is the current known optimal function value, $\Phi(\bullet)$ is the cumulative distribution function of the standard normal distribution, $\sigma(x)$ is the standard deviation of the Gaussian process at point x, and $\varphi(\bullet)$ is the probability density function of the standard normal distribution. However, when tackling the complex PDE problems of this work, the expected improvement (EI) criterion tends to become trapped in local optima. To overcome this limitation, the EI-Plus (EIP) acquisition function[4] is adopted. EIP incorporates a self-correcting mechanism to prevent over-exploitation, which is mathematically expressed as:

$$\sigma_Q{}^2(x) = \sigma_F{}^2(x) + \sigma^2,\ for\ \sigma_F(x) < t_\sigma \sigma, \quad (2)$$

where $\sigma_F(x)$ denotes the standard deviation of the posterior objective function at x while σ represents the posterior standard deviation of the additive noise. The adjusted standard deviation is given by $\sigma_Q(x)$, and t_σ (set to 0.8 in this work) defines the adjustable exploration–exploitation trade-off ratio. If the condition of $\sigma_F(x) < t_\sigma \sigma$ is not satisfied, the algorithm adaptively increases the variance between observation points by modifying the kernel function.[4]

1.2. Pattern search algorithm

In the local exploitation phase, gradient descent, Newton's method, and quasi-Newton methods are among the most widely used local optimization techniques due to their broad applicability and favorable convergence properties. However, these methods require the

objective function to be continuous and differentiable, making them heavily reliant on derivative information—an impractical assumption for complex PDE problems. In contrast, the pattern search algorithm provides a derivative-free alternative that is computationally efficient, straightforward to implement, and inherently more robust, making it well-suited for addressing the optimal design problem of membrane module in this work.

The pattern search algorithm determines search directions using pattern vectors. In the context of the optimal design of membrane module in this work, where the decision variables have six dimensions, 12 fundamental search directions are defined as a positively definite extension set $[d_1, d_2, \cdots, d_{11}, d_{12}]$ where $d_1 = (1, 0, ..., 0)$, $d_2 = (-1, 0, ..., 0)$ ..., $d_{12} = (0, ..., 0, -1)$. This formulation ensures comprehensive exploration across the search space. The algorithm employs pattern directions and the current grid size to systematically search around the incumbent solution. Here, the grid represents a conceptual mesh of candidate points from which the algorithm selects evaluation points. To enhance local refinement, we initialize the pattern search algorithm at the optimized solution identified through Bayesian optimization and adopt a smaller initial grid size, facilitating more precise local exploration.

If the search step fails to identify an improved solution, the algorithm proceeds to a polling step. Similar to the exploration phase, this step systematically evaluates alternative positions; however, instead of relying on a subset of the previously defined positive definite extension set, it sequentially traverses each direction. If a polling point yields a better objective value than the current position, it is adopted as the new incumbent solution, the grid size is increased, and the algorithm resumes the search step. Accordingly, if the polling

step fails to identify an improved solution, the current position remains unchanged, the grid size is reduced, and the search step is reinitiated. The algorithm terminates upon meeting the predefined convergence criteria, which include a minimum grid size tolerance of 10^{-10}, stabilization of the objective function value within a threshold of 10^{-7} over five consecutive iterations, and a maximum iteration limit. These criteria ensure high solution accuracy while enhancing the adaptability of the algorithm.

Supplementary Note S2

2.1. 3D high-fidelity multi-physics simulations

In spiral-wound membrane systems, each module consists of over a million feed spacer cells, making full-scale 3D multi-physics simulations computationally infeasible. However, prior research indicates that flow and mass transfer reach a periodic fully-developed state within just a few spacer cells[5]. This allows for a substantial reduction in computational complexity by focusing on a localized domain while still capturing the essential hydrodynamic and transport characteristics. To systematically evaluate system performance, the mass transfer coefficient and axial pressure drop per unit length are assessed at different positions along the feed direction by modulating the inlet feed velocity. In this study, high-fidelity 3D multi-physics models is employed to resolve fluid flow and mass transport within a computational domain consisting of five unit cells (Figure S1), providing an efficient yet accurate framework for analyzing local transport phenomena for water production process (3) and flushing process (4) during the flushing process of batch reverse osmosis, as below:

$$
\begin{cases}
\rho(\mathbf{u}\cdot\nabla)\mathbf{u}=\nabla\cdot[-P\mathbf{I}+\mu(\nabla\mathbf{u}+(\nabla\mathbf{u})^{\mathrm{T}})], & \text{in } \Omega,\\
\nabla\cdot(\rho\mathbf{u})=0, & \text{in } \Omega,\\
\nabla\cdot(D_s\nabla c)=\mathbf{u}\cdot\nabla c, & \text{in } \Omega,\\
\mathbf{u}_{\mathrm{in}}=\mathbf{u}_{\mathrm{out}},\,(\nabla\mathbf{u})_{\mathrm{in}}=(\nabla\mathbf{u})_{\mathrm{out}},\,\bar{u}_{\mathrm{ave,\,in}}=U_0, & \text{on } (\boldsymbol{\Gamma}_I\cup\boldsymbol{\Gamma}_O),\\
(\nabla P)_{\mathrm{in}}=(\nabla P)_{\mathrm{out}},\,P_{\mathrm{out}}=0, & \text{on } (\boldsymbol{\Gamma}_I\cup\boldsymbol{\Gamma}_O),\\
\mathbf{u}_L=\mathbf{u}_{\mathrm{R}},\,(\nabla\mathbf{u})_L=(\nabla\mathbf{u})_R, & \text{on } (\boldsymbol{\Gamma}_L\cup\boldsymbol{\Gamma}_R),\\
P_L=P_{\mathrm{R}},\,(\nabla P)_L=(\nabla P)_R, & \text{on } (\boldsymbol{\Gamma}_L\cup\boldsymbol{\Gamma}_R),\\
\mathbf{u}=\mathbf{0}, & \text{on } (\boldsymbol{\Gamma}_U\cup\boldsymbol{\Gamma}_B\cup\boldsymbol{\Gamma}_S),\\
c_{\mathrm{in}}=c_0,\,(-\mathbf{n}\cdot D_{\mathrm{s}}\nabla c)_{\mathrm{out}}=0, & \text{on } (\boldsymbol{\Gamma}_I\cup\boldsymbol{\Gamma}_O),\\
c_L=c_R,\,(\nabla c)_L=(\nabla c)_R, & \text{on } (\boldsymbol{\Gamma}_L\cup\boldsymbol{\Gamma}_R),\\
\mathbf{n}\cdot(-D_{\mathrm{s}}\nabla c+c\mathbf{u})=0, & \text{on } (\boldsymbol{\Gamma}_U\cup\boldsymbol{\Gamma}_B\cup\boldsymbol{\Gamma}_S),
\end{cases}
\tag{3}
$$

$$
\begin{cases}
\rho(\mathbf{u}\cdot\nabla)\mathbf{u}=\nabla\cdot[-P\mathbf{I}+\mu(\nabla\mathbf{u}+(\nabla\mathbf{u})^{\mathrm{T}})], & \text{in } \Omega,\\
\nabla\cdot(\rho\mathbf{u})=0, & \text{in } \Omega,\\
\dfrac{\partial c}{\partial t}=\nabla\cdot(D_s\nabla c)-\mathbf{u}\cdot\nabla c, & \text{in } \Omega\times[0,t_{\mathrm{f}}],\\
c\big|_{t=0}=2c_0, & \text{on } \Omega,\\
\mathbf{u}_{\mathrm{in}}=\mathbf{u}_{\mathrm{out}},\,(\nabla\mathbf{u})_{\mathrm{in}}=(\nabla\mathbf{u})_{\mathrm{out}},\,\bar{u}_{\mathrm{ave,\,in}}=U_0, & \text{on } (\boldsymbol{\Gamma}_I\cup\boldsymbol{\Gamma}_O),\\
(\nabla P)_{\mathrm{in}}=(\nabla P)_{\mathrm{out}},\,P_{\mathrm{out}}=0, & \text{on } (\boldsymbol{\Gamma}_I\cup\boldsymbol{\Gamma}_O),\\
\mathbf{u}_L=\mathbf{u}_R,\,(\nabla\mathbf{u})_L=(\nabla\mathbf{u})_R, & \text{on } (\boldsymbol{\Gamma}_L\cup\boldsymbol{\Gamma}_R),\\
P_L=P_R,\,(\nabla P)_L=(\nabla P)_R, & \text{on } (\boldsymbol{\Gamma}_L\cup\boldsymbol{\Gamma}_R),\\
\mathbf{u}=\mathbf{0}, & \text{on } (\boldsymbol{\Gamma}_U\cup\boldsymbol{\Gamma}_B\cup\boldsymbol{\Gamma}_S),\\
c_{\mathrm{in}}=c_0,\,(-\mathbf{n}\cdot D_{\mathrm{s}}\nabla c)_{\mathrm{out}}=0, & \text{on } (\boldsymbol{\Gamma}_I\cup\mathit{\Gamma}_O)\times(0,t_{\mathrm{f}}),\\
c_L=c_R,\,(\nabla c)_L=(\nabla c)_R, & \text{on } (\boldsymbol{\Gamma}_L\cup\boldsymbol{\Gamma}_R)\times(0,t_{\mathrm{f}}),\\
\mathbf{n}\cdot(-D_{\mathrm{s}}\nabla c+c\mathbf{u})=0, & \text{on } (\boldsymbol{\Gamma}_U\cup\boldsymbol{\Gamma}_B\cup\boldsymbol{\Gamma}_S)\times(0,t_{\mathrm{f}}).
\end{cases}
\tag{4}
$$

The computational domain (Ω) is defined within a narrow spacer-filled channel, as shown in Figure S1, where the boundaries $\boldsymbol{\Gamma}_I$, $\boldsymbol{\Gamma}_O$, $\boldsymbol{\Gamma}_L$, $\boldsymbol{\Gamma}_R$, $\boldsymbol{\Gamma}_U$, $\boldsymbol{\Gamma}_B$ and $\boldsymbol{\Gamma}_S$ are depicted. The total simulation time in Eq. (4) is denoted as t_{f}. The fluid properties, including density (ρ), viscosity (μ), and salt diffusivity (D_{s}), are assumed to remain constant throughout the

simulation. The key variables governing the system include the velocity vector (**u**), hydraulic pressure (*P*), and molar concentration (*c*).

2.2. Hydrodynamics and transport characteristics

The Darcy friction factor is computed based on flow characteristics, including cross velocity ($\bar{u}$), axial pressure drop (ΔP_{c}), and spacer geometry, as below[6]

$$f = \frac{2D_{\mathrm{H}}\Delta P_{\mathrm{c}}}{\rho \bar{u}^2 L}, \tag{5}$$

where D_{H} and L represent the hydraulic diameter and the computational domain's channel length, respectively (Figure S1). $\Delta P_c / L$ and D_{H} are estimated by

$$-\frac{\Delta P_c}{L} = -\frac{\bar{P}_{\mathrm{out}} - \bar{P}_{\mathrm{in}}}{L}, \tag{6}$$

and

$$D_H = \frac{4\varepsilon}{2/H + (1-\varepsilon)S_{\mathrm{sp}}/V_{\mathrm{sp}}}, \tag{7}$$

respectively where S_{sp} and V_{sp} denote surface area and volume of spacers in feed channel. H is channel height. Channel porosity ε is defined as

$$\varepsilon = \frac{V_{\mathrm{tot}} - V_{\mathrm{sp}}}{V_{\mathrm{tot}}}, \tag{8}$$

which V_{tot} is the volume of rectangular prism (Figure S1). The Sherwood number (*Sh*), determined as a function of cell-average mass transfer coefficient ($\bar{k}_{\mathrm{m}}$) on membrane walls can be expressed as

$$Sh = \bar{k}_{\mathrm{m}} \frac{D_{\mathrm{H}}}{D_{\mathrm{s}}}. \tag{9}$$

$\bar{k}_{\mathrm{m}}$ is defined by

$$\bar{k}_{\mathrm{m}} = \frac{\int_0^L \mathrm{d}y \int_0^W \left(\frac{-D_{\mathrm{s}}}{c_{\mathrm{r}} - c_{\mathrm{w}}} \cdot \frac{\partial c}{\partial z}\right) \mathrm{d}x}{\int_0^L \mathrm{d}y \int_0^W \mathrm{d}x}. \tag{10}$$

Here, c_{r} and c_{w} represent the solute concentration in the bulk retentate and at the membrane surface, respectively.

Using CFD simulations across a wide range of Reynolds numbers (*Re* = 50, 62.5, 75 … 1,000), we derive empirical correlations for the Sherwood number and Darcy friction factor. These correlations characterize mass transfer and flow resistance within the spacer-filled channel and are expressed as follows:

$$Sh_{\mathrm{imp}} = kRe^{t}, \tag{11}$$

$$f = \sum_{i=1}^{8} (a_i Re^{i} + b_0), \tag{12}$$

The Reynolds number is determined as a function of crossflow velocity, incorporating fluid properties and channel geometry to characterize flow behavior within the spacer-filled domain, as following

$$Re = \frac{D_{\mathrm{H}} \bar{u} \rho}{\mu}. \tag{13}$$

The correlations (11) and (12) establish a quantitative framework for analyzing mass transfer and flow resistance, facilitating the optimal system design.

2.3. Flushing efficacy for batch RO

Batch reverse osmosis operates in three phases: water production, flushing and refill. During the flushing process, incomplete flushing causes salt retention, which impairs

performance by increasing salt concentration, raising peak pressure, and elevating power consumption. The flushing efficacy (f) is estimated using the following formula[7]

$$f\left(\theta\right)=\int_0^\theta\left(1-F\left(\theta\right)\right)\mathrm{d}\theta. \tag{14}$$

The cumulative function of residence time distribution (CRTD), $F\left(t\right)$ is evaluated from[7]

$$F\left(t\right)=\frac{\iint_{\mathrm{out}}c_{\mathrm{out}}(t)u\mathrm{d}y\mathrm{dz}}{c_{\mathrm{in}}\iint_{\mathrm{in}}u\mathrm{d}y\mathrm{dz}}, \tag{15}$$

The residence time distribution (RTD), $E\left(t\right)$ is determined by[7]

$$E\left(t\right)=\frac{\mathrm{d}F\left(t\right)}{\mathrm{d}t}, \tag{16}$$

Here, c_{in} represents the constant inlet concentration, while outlet concentration of $c_{\mathrm{out}}\left(t\right)$ varies over time. To evaluate the influence of design parameters on RTD, CRTD, and flushing efficiency, the dimensionless time is defined as $\theta = t/\tau$. The space time (τ) is determined using the following equation

$$\tau=\frac{\iiint_{\Omega}\mathrm{d}x\mathrm{d}y\mathrm{dz}}{\iint_{in}u\mathrm{d}y\mathrm{dz}}, \tag{17}$$

which u denote the velocity alone the feed direction (Figure S1). In a full-scale RO system, where multiple RO elements are arranged in series, the overall RTD is determined through a convolution operation[7], expressed as follows:

$$\begin{aligned}
c_{\mathrm{out}}^{1}\left(t\right)&=conv\left(c_{\mathrm{in}}^{1}\left(t\right),E_{\mathrm{in}}^{1}\left(t\right)\right),\\
c_{\mathrm{out}}^{2}\left(t\right)&=conv\left(c_{\mathrm{out}}^{1}\left(t\right),E_{\mathrm{out}}^{1}\left(t\right)\right),\\
&\vdots\\
c_{\mathrm{out}}^{n}\left(t\right)&=conv\left(c_{\mathrm{out}}^{n\text{-}1}\left(t\right),E_{\mathrm{out}}^{n\text{-}1}\left(t\right)\right),
\end{aligned} \tag{18}$$

Here, $c_{\text{out}}^{n\text{-}1}$ and $E_{\text{out}}^{n\text{-}1}$ ($n \in N^+$) represent the outlet concentration and RTD, respectively, for the (n-1)th membrane module within a pressure vessel. Similarly, c_{in}^{1} and E_{in}^{1} denote the inlet concentration and RTD for the first membrane module. Since all membrane elements in the RO system share identical geometric structures, they exhibit the same RTD, meaning $E_{\text{in}}^{1}(t) = E_{\text{out}}^{1}(t) = \ldots = E_{\text{out}}^{n\text{-}1}(t) = E(t)$. $E(t)$ is the RTD through CFD simulations in this work.

Supplementary Note S3

3.1. System modeling at industry-scale

At the industrial scale, the RO process can be mathematically modeled using one-dimensional (1D) differential-algebraic equations (DAEs).[8] The governing equations for k^{th} stage of the RO process are formulated as follows

$$\begin{cases} \dfrac{\mathrm{d}Q}{\mathrm{d}X} = -J_{\mathrm{W}} \cdot A_k & X = k-1, Q = Q_{k-1}, \\ \dfrac{\mathrm{d}(\Delta P)}{\mathrm{d}X} = -\dfrac{\rho \bar{u}^2 f}{2D_H} \cdot \left(n_{\text{mem},\, k} \cdot l_y\right) & X = k-1, \Delta P = \Delta P_{k-1}, \\ \dfrac{\mathrm{d}w_{\mathrm{b}}}{\mathrm{d}X} = J_{\mathrm{W}} \cdot \dfrac{A_k}{Q}\left(w_{\mathrm{b}} - w_{\mathrm{p}}\right) & X = k-1, w_{\mathrm{b}} = w_{\mathrm{b},\, k-1}, \\ J_{\mathrm{W}} = L_{\mathrm{P}}\left(\Delta P - \sigma \cdot \varphi R_{\text{salt}} w_{\mathrm{w}}\right) & \end{cases} \tag{19}$$

In Equation (19), the primary variables to be determined include the flow rate (Q), transmembrane pressure (ΔP), water flux (J_{w}), and salinity concentrations at different points: within the retentate bulk (w_{b}), permeate bulk (w_{p}), and along the membrane surface (w_{w}). These parameters evolve along the dimensionless axial coordinate ($X \in [k-1, k]$),

which represents the normalized position along the membrane length. The expression for Q is given by:

$$Q = N_{\text{pv},k} n_{\text{sp}} l_x H \varepsilon \bar{u}, \tag{20}$$

In this formulation, $N_{\text{pv},k}$ refers to the number of pressure vessels in a given stage, while n_{sp} indicates the number of spacer sheets per module. The parameters l_x, H and ε represent the membrane sheet length perpendicular to the feed flow direction (*x* direction, Figure S1), the height of each feed channel, and the porosity of the feed channel, respectively. The total membrane surface area (A_k) for a given stage, k^{th}, is determined using the following equation

$$A_k = N_{\text{pv},k} \cdot n_{\text{mem},k} \cdot A_0 \cdot l_y / l_{y,0}, \tag{21}$$

In this model, $n_{\text{mem},k}$ represents the number of modules in a given stage of kth. The parameters l_y and $l_{y,0}$ refer to the membrane sheet lengths along the feed flow direction (*y*-direction, in Figure S1) for the optimized module (0.5 m) and the commercial module (1 m), respectively. The membrane area of a commercial module is denoted as A_0. The reflection coefficient (σ) and osmotic pressure coefficient (φ) are considered constants. Additionally, R_{salt} represents the intrinsic rejection efficiency of the membrane. The values of w_{p} and w_{w} can be calculated using the following equations[8]

$$w_{\text{p}} = w_{\text{b}} / \left[\exp\left(\ln \frac{J_{\text{W}}}{B} - \frac{J_{\text{W}}}{\bar{k}_{\text{m, per}}} \right) + 1 \right], \tag{22}$$

and

$$w_{\mathrm{w}} = \frac{w_{\mathrm{p}}}{1 - R_{\mathrm{salt}}}, \tag{23}$$

respectively.

The derivation of Eq. (22) is provided in our previous work.[8] The term $\bar{k}_{\mathrm{m,\,per}}$ represents the cell-averaged mass transfer coefficient on the permeable wall, which can be estimated using Eqs. (24) and (25)[9], as shown below

$$\bar{k}_{\mathrm{m,\,per}} = \bar{k}_{\mathrm{m}} \left[\psi + \left(1 + 0.26\psi^{1.4} \right)^{-1.7} \right], \quad (\psi < 20) \tag{24}$$

$$\psi = \frac{J_{\mathrm{W}}}{\bar{k}_{\mathrm{m}}}. \tag{25}$$

Here, $\bar{k}_{\mathrm{m}}$ for a given system can can be determined using Eqs. (10), (11), and (13), based on the CFD simulations. By combining Eqs. (19) to (23), the system-level variables (Q, ΔP, J_{W}, w_{b}, w_{p}, w_{w}) can be solved.

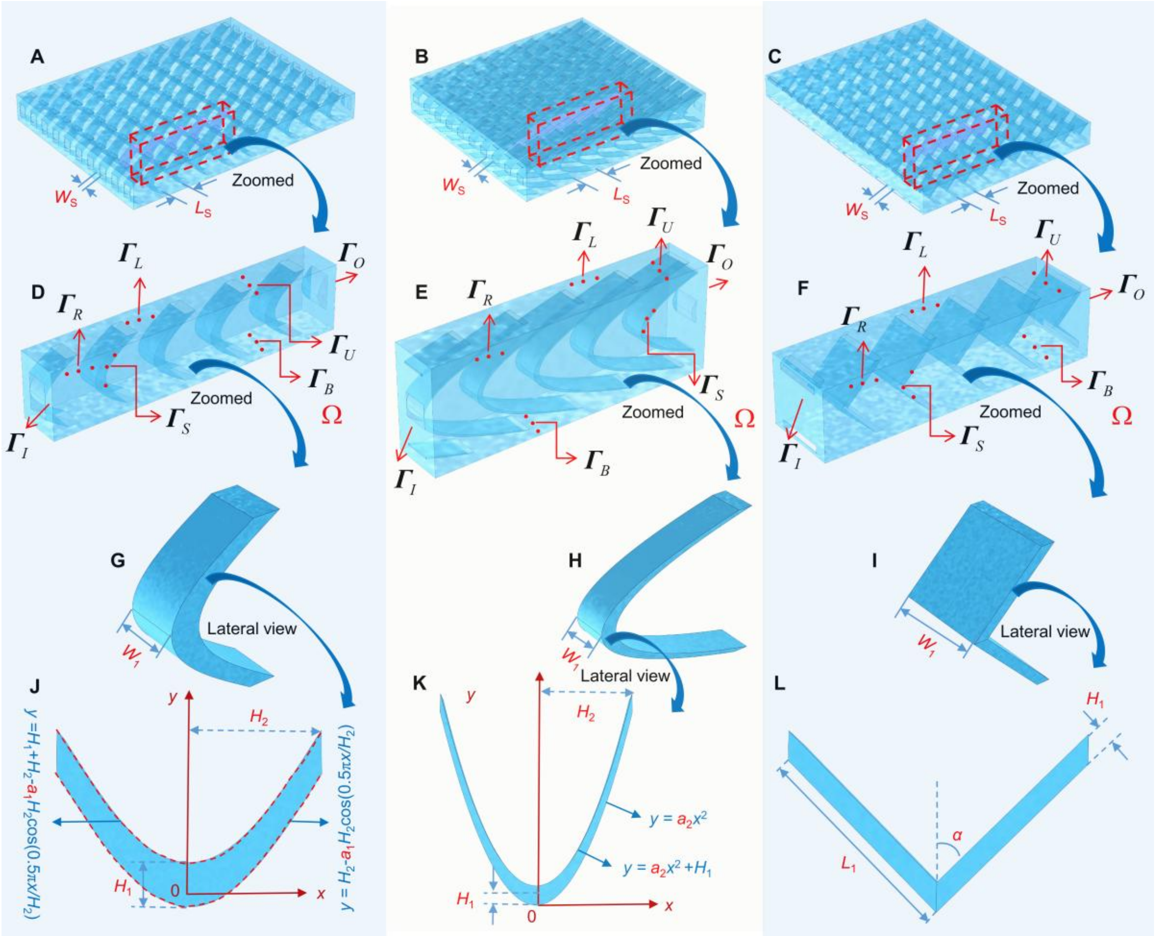

Figure S1. Establishment of parametric geometric models. Small segments of spacer sheets designed for **A**, a cosine shape, **B**, a parabolic shape and **C**, a V-shape. Computational domains of three-dimensional multi-physics model for **D**, a cosine shape, **E**, a parabolic shape and **F**, a V-shape. Spacer cells corresponding to **G**, a cosine shape, **H**, a parabolic shape and **I**, a V-shape. Lateral views of the spacer cells for **J**, a cosine shape, **K**, a parabolic shape and **L**, a V-shape. The cosine-shaped spacers-filled channel is characterized by distance parameters (L_S, W_S), size parameters (H_1, H_2, W_1), and a shape parameter (a_1). The parabolic-shaped spacers-filled channel is defined by distance parameters (L_S, W_S), size parameters (H_1, H_2, W_1), and a shape parameter (a_2). Specifically, the V-shaped spacers-filled channel is defined by distance parameters (L_S, W_S), size parameters (L_1, H_1, W_1), and a shape parameter (α).

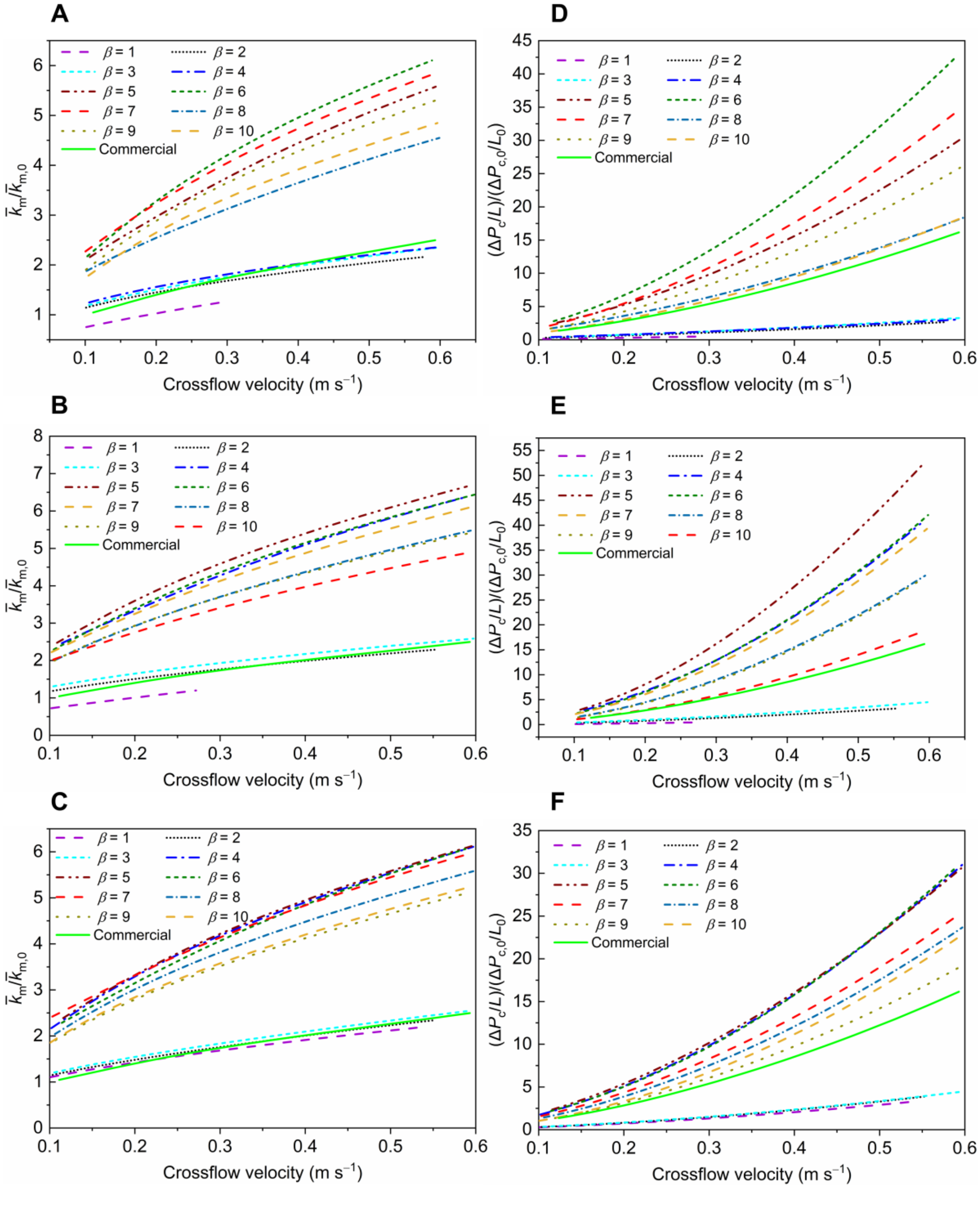

Figure S2. Mass transfer and pressure drop of optimized and commercial spacers. Ratios of cell-averaged mass transfer coefficients ($\bar{k}_m$) for optimized spacers with **A**, V-shape, **B**, cosine-shape, and **C**, parabola-shape, compared to the commercial spacer ($\bar{k}_{m,0}$). Ratios of pressure drops per meter ($\Delta P_c / L$) for optimized spacers with **D**, V-shape, **E**, cosine-shape, and **F**, parabola-shape, relative to the commercial spacer ($\Delta P_{c,0} / L_0$).

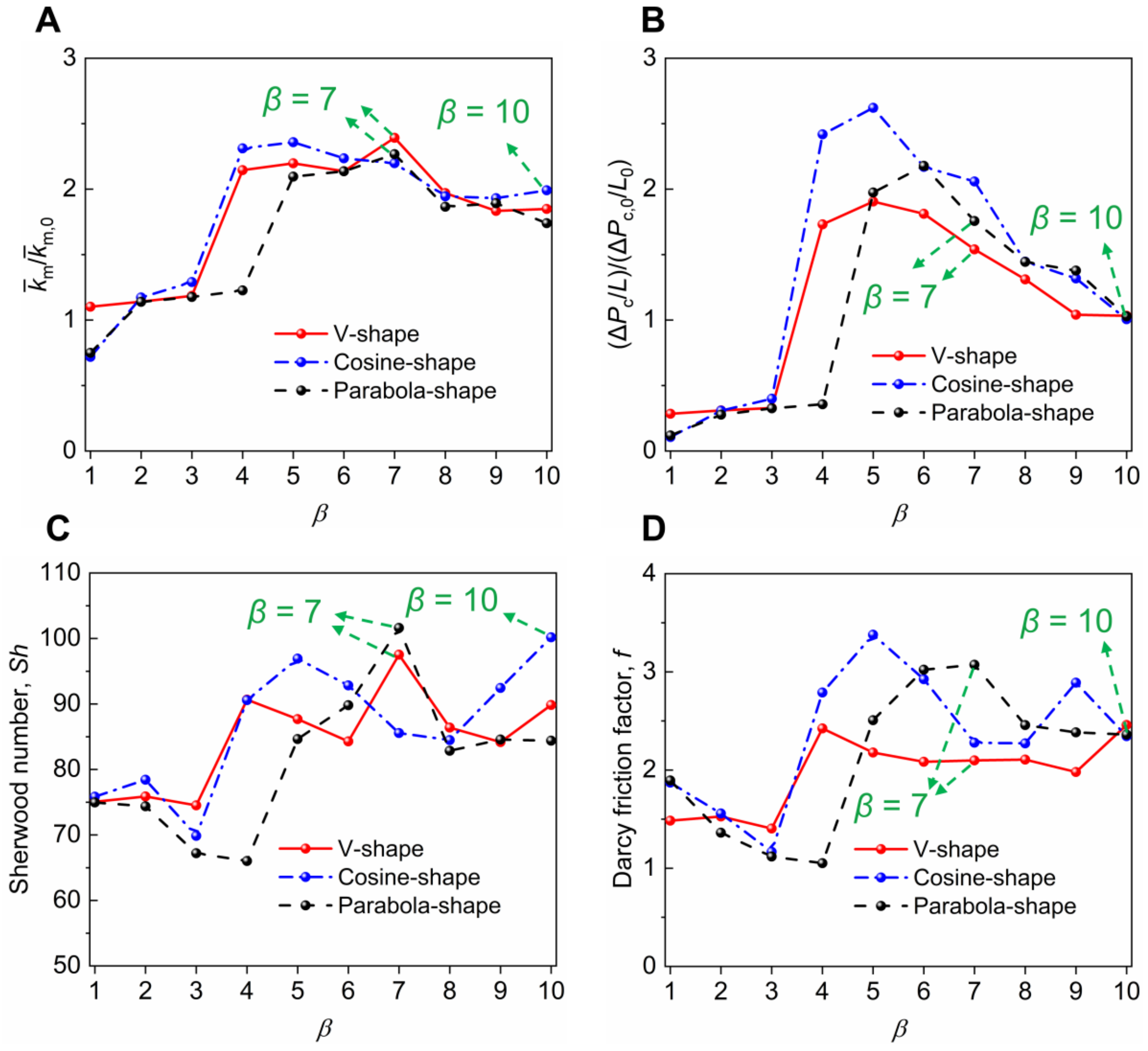

Figure S3. Identification of optimal tradeoff parameters. A, Ratios of cell-averaged mass transfer coefficients for the optimized spacer ($\bar{k}_{\mathrm{m}}$) versus the commercial spacer ($\bar{k}_{\mathrm{m,0}}$) as a function of β. **B,** Ratios of pressure drops per meter for the optimized spacer ($\Delta P_{\mathrm{c}} / L$) versus the commercial spacer ($\Delta P_{\mathrm{c,0}} / L_0$) as a function of β. **C**, Sherwood number for the optimized spacers as a function of β. **D**, Darcy friction factor for the optimized spacers as a function of β. β represents the tradeoff parameter in the objective function (F_1) to balance pressure drop and mass transfer. A detailed mathematical formulation of F_1 is provided in the *MATERIALS AND METHODS* section. The cross velocity is set to 0.1 m s^{-1} in **A** and **B**, while the Reynolds number is fixed at 100 in **C** and **D**.

Table S1. The range of geometric design parameters. The design parameters are represented as $\boldsymbol{\beta}_1 = [L_S, W_S, a_1, W_1, H_1, H_2]$ for cosine-shape, $\boldsymbol{\beta}_1 = [L_S, W_S, a_2, W_1, H_1, H_2]$ for parabolic-shaped, and $\boldsymbol{\beta}_1 = [L_S, W_S, \alpha, W_1, H_1, L_1]$ for V-shaped membrane modules, respectively, as shown in Figure S1.

**Range of design parameters**		**Value**					
		L_S (μm)	W_S (μm)	a_1 (m) or a_2 (m^{-1}) or α (°)	W_1 (μm)	H_1 (μm)	H_2 (μm) or L_1 (μm)
Cosine-shape	Minimum	400	200	0.25	100	10	300
	Maximum	2,000	1,000	1.25	1,000	100	1,000
Parabola-shape	Minimum	400	200	800	100	10	300
	Maximum	2,000	1,000	3,000	1,000	100	1,000
V-shape	Minimum	400	200	25	100	10	300
	Maximum	2,000	1,000	70	1,000	100	1,000

Table S2. Optimized outcomes for cosine-, parabolic-, and V-shape spacers. Geometric design parameters and computational domain size are investigated in relation to various trade-off parameter values of β. The design parameters are represented as $\boldsymbol{\beta}_1 = [L_S, W_S, a_1, W_1, H_1, H_2]$ for cosine-shape, $\boldsymbol{\beta}_1 = [L_S, W_S, a_2, W_1, H_1, H_2]$ for parabolic-shaped, and $\boldsymbol{\beta}_1 = [L_S, W_S, \alpha, W_1, H_1, L_1]$ for V-shaped membrane modules, respectively, as shown in Figure S1. The computational domain size consists of channel length (L), width (W) and height (H).

**Cases**		**Optimized results**								
		Geometric design parameters ($\boldsymbol{\beta}_1$)						Computational domain size		
		L_S (μm)	W_S (μm)	a_1 (m) or a_2 (m^{-1}) or α (°)	W_1 (μm)	H_1 (μm)	H_2 (μm) or L_1 (μm)	L (mm)	W (mm)	H (mm)
Cosine-shape	$\beta = 1$	1,966	964	1.24	102	14	993	9.90	1.07	1.99
	$\beta = 2$	411	1,000	1.05	102	17	597	2.14	1.10	1.19
	$\beta = 3$	403	990	1.07	101	28	399	2.15	1.09	0.80
	$\beta = 4$	409	252	1.09	132	14	340	2.11	0.38	0.68
	$\beta = 5$	501	274	1.09	217	26	401	2.63	0.49	0.80
	$\beta = 6$	563	274	1.16	190	13	357	2.88	0.46	0.71
	$\beta = 7$	472	312	1.20	179	17	304	2.44	0.49	0.61
	$\beta = 8$	583	408	1.18	223	13	372	2.98	0.63	0.74
	$\beta = 9$	887	468	1.22	359	12	491	4.49	0.83	0.98
	$\beta = 10$	657	449	1.10	212	38	510	3.48	0.66	1.02
Parabola-shape	$\beta = 1$	1,816	994	1,511	105	68	984	9.42	1.10	1.97
	$\beta = 2$	411	972	1,023	111	73	583	2.42	1.08	1.17
	$\beta = 3$	478	947	2,304	110	66	445	2.72	1.06	0.89
	$\beta = 4$	405	989	1,631	119	53	393	2.29	1.11	0.79
	$\beta = 5$	414	288	2,924	133	33	365	2.24	0.42	0.73
	$\beta = 6$	558	315	2,880	240	36	369	2.97	0.55	0.74
	$\beta = 7$	644	378	2,567	261	22	444	3.33	0.64	0.89
	$\beta = 8$	400	322	1,828	106	54	414	2.27	0.43	0.83
	$\beta = 9$	727	400	2,931	295	61	422	3.94	0.69	0.84
	$\beta = 10$	836	440	2,436	272	50	511	4.43	0.71	0.51
V-shape	$\beta = 1$	413	998	52.7	106	65	828	2.48	1.10	1.32
	$\beta = 2$	404	997	48.7	116	51	879	2.36	1.11	1.32
	$\beta = 3$	418	988	41.9	110	31	850	2.32	1.10	1.14
	$\beta = 4$	576	306	33.5	219	11	777	2.98	0.52	0.86
	$\beta = 5$	565	279	30.4	205	12	753	2.94	0.48	0.76
	$\beta = 6$	407	297	37.5	135	11	550	2.12	0.43	0.67
	$\beta = 7$	598	301	31.6	188	11	750	3.09	0.49	0.79
	$\beta = 8$	652	374	32.5	230	10	794	3.35	0.60	0.85
	$\beta = 9$	753	455	31.4	272	10	895	3.86	0.73	0.93
	$\beta = 10$	712	498	37.3	304	13	983	3.66	0.80	1.19

Table S3. Repeatability test of V-shaped feed spacer design for geometric parameters. Geometric design parameters and computational domain size are investigated in relation to a trade-off parameter value of β = 7. The design parameters are represented as $\boldsymbol{\beta}_1 = \left[L_S, W_S, \alpha, W_1, H_1, L_1\right]$ for V-shaped membrane modules, as shown in Figure S1. The computational domain size consists of channel length (L), width (W) and height (H).

	Geometric design parameters ($\boldsymbol{\beta}_1$)						Computational domain size		
	L_S (μm)	W_S (μm)	α (°)	W_1 (μm)	H_1 (μm)	L_1 (μm)	L (mm)	W (mm)	H (mm)
Optimized	598	301	31.6	188	11	750	3.09	0.49	0.79
Repeatability test	562	314	34.2	188	11	719	2.91	0.51	0.81

Table S4. Repeatability test of V-shaped feed spacer design for Sherwood numbers with respect to various Reynolds numbers. The tradeoff parameter in the objective function (F_1) is chosen as $\beta = 7$ to balance pressure drop and mass transfer.

Re	Sherwood number		Relative deviation
	Optimized	Repeatability test	
50	76	75	0.1%
100	98	98	0.4%
150	118	120	1.8%
200	138	142	2.5%
250	156	161	2.7%
300	173	177	2.9%
350	187	193	3.0%
400	201	207	3.1%
450	214	221	3.1%
500	226	234	3.2%
550	238	246	3.3%
600	249	258	3.3%

Table S5. Sensitivity analysis for geometric design parameters. The parameters, defined as $\boldsymbol{\beta}_1 = [L_S, W_S, \alpha, W_1, H_1, L_1]$, describe the V-shaped membrane modules under a cross-flow velocity of 0.1 m s^{-1}. A design optimized at β = 7 serves as the benchmark: $\boldsymbol{\beta}_{1,\,\mathrm{opt}}$ = $[598\,\mu\mathrm{m}, 301\,\mu\mathrm{m}, 31.6^\circ, 188\,\mu\mathrm{m}, 11\,\mu\mathrm{m}, 750\,\mu\mathrm{m}]$ where $\bar{k}_\mathrm{m} / \bar{k}_{\mathrm{m},\,0}$ and $\frac{\Delta P_\mathrm{c} / L}{\Delta P_{\mathrm{c},\,0} / L_0}$ are 2.39 and 1.54 respectively. For the sensitivity analysis, each parameter is varied individually while the others remain fixed at $\boldsymbol{\beta}_{1,\,\mathrm{opt}}$.

Parameter	Value									
L_S (μm)	400	600	800	1,000	1,200	1,400	1,600	1,800	2,000	
$\bar{k}_\mathrm{m} / \bar{k}_{\mathrm{m},\,0}$	2.56	2.38	2.15	1.98	1.83	1.76	1.68	1.61	1.52	
$\frac{\Delta P_\mathrm{c} / L}{\Delta P_{\mathrm{c},\,0} / L_0}$	2.20	1.54	1.22	1.09	1.00	0.90	0.81	0.74	0.68	
W_S (μm)	200	300	400	500	600	700	800	900	1,000	
$\bar{k}_\mathrm{m} / \bar{k}_{\mathrm{m},\,0}$	2.62	2.40	2.19	2.02	1.87	1.72	1.66	1.62	1.52	
$\frac{\Delta P_\mathrm{c} / L}{\Delta P_{\mathrm{c},\,0} / L_0}$	2.39	1.55	1.13	0.88	0.71	0.60	0.52	0.47	0.43	
α (°)	25	30	35	40	45	50	55	60	65	70
$\bar{k}_\mathrm{m} / \bar{k}_{\mathrm{m},\,0}$	2.35	2.39	2.33	2.26	2.18	2.06	1.97	1.90	1.82	1.84
$\frac{\Delta P_\mathrm{c} / L}{\Delta P_{\mathrm{c},\,0} / L_0}$	1.69	1.57	1.51	1.48	1.48	1.49	1.53	1.61	1.75	1.80
W_1 (μm)	100	200	300	400	500	600	700	800	900	1,000
$\bar{k}_\mathrm{m} / \bar{k}_{\mathrm{m},\,0}$	2.18	2.41	2.53	2.61	2.65	2.68	2.70	2.71	2.71	2.73
$\frac{\Delta P_\mathrm{c} / L}{\Delta P_{\mathrm{c},\,0} / L_0}$	1.04	1.63	2.39	3.29	4.34	5.54	6.86	8.25	9.69	11.14
H_1 (μm)	10	20	30	40	50	60	70	80	90	100
$\bar{k}_\mathrm{m} / \bar{k}_{\mathrm{m},\,0}$	2.39	2.30	2.28	2.30	2.26	2.24	2.21	2.20	2.18	2.16
$\frac{\Delta P_\mathrm{c} / L}{\Delta P_{\mathrm{c},\,0} / L_0}$	1.54	1.53	1.53	1.52	1.52	1.51	1.51	1.51	1.51	1.52
L_1 (μm)	300	400	500	600	700	800	900	1,000		
$\bar{k}_\mathrm{m} / \bar{k}_{\mathrm{m},\,0}$	2.61	2.56	2.46	2.46	2.41	2.36	2.33	2.27		
$\frac{\Delta P_\mathrm{c} / L}{\Delta P_{\mathrm{c},\,0} / L_0}$	3.26	2.49	2.05	1.75	1.59	1.51	1.46	1.43		

Table S6. The range of system design parameters for two-stage ultrapermeable seawater reverse osmosis.

Design Parameters			Maximum	Minimum
Membrane module	Number of feed spacers per element	n_{sp}	30	5
First-stage RO system	Transmembrane pressure	ΔP_0 (bar)	$\Delta P_{0,\,min}$ (Refer to our previous work[8])	0
	Number of modules per pressure vessel	$n_{mem,\,1}$	20	4
	Number of pressure vessels	$N_{pv,\,1}$	30	5
Second-stage RO system	Transmembrane pressure	ΔP_1 (bar)	$\Delta P_{0,\,min}$ +10 bar	$\Delta P_{0,\,min}$
	Number of modules per pressure vessel	$n_{mem,\,2}$	20	4
	Number of pressure vessels	$N_{pv,\,2}$	$N_{pv,\,1}$	$\text{ceil}\left(N_{pv,\,1}/3\right)^{\#}-1$
Membrane properties	Water permeability	L_p (L m^{-2} h^{-1} bar^{-1})	100	1
	Salt permeability	B (L m^{-2} h^{-1})	$0.1\times L_p\times\text{bar}$	0.1

$^{\#}$ ceil: rounding towards positive infinity

Table S7. Optimized two-stage ultrapermeable membrane systems with a limited concentration polarization (no more than 1.20). The analysis is based on a feed salinity of 35,000 ppm, a water recovery rate of 50%, pump efficiency of 85%, and energy recovery efficiency of 95%. The original results are from a conventional one-stage SWRO system with a commercial membrane module under standard engineering conditions. In contrast, optimized results are obtained from a two-stage SWRO system using ultrapermeable membranes and optimized spacer configurations for tradeoff parameter c_m values (40, 220, and 400 \$ m^{-2}), with a maximum concentration polarization factor of 1.20. The geometric specifications of the commercial membrane module, with a 28 mil feed spacer, are detailed in previous work.[10]

Parameters				Value			
				Original	Optimized c_m = 40 \$ m^{-2}	c_m = 220 \$ m^{-2}	c_m = 400 \$ m^{-2}
Design parameters	Geometrical parameters of feed spacers		L_1 (μm)	---	750	750	750
			W_1 (μm)	---	188	188	188
			H_1 (μm)	---	11	11	11
			α (°)	---	31.6	31.6	31.6
			L_S (μm)	---	598	598	598
			W_S (μm)	---	301	301	301
	First-stage RO system	Inlet transmembrane pressure	ΔP_0 (bar)	65.0	40.7	42.7	44.0
		Number of modules per pressure vessel	$n_{mem,1}$	7	4	4	4
		Number of pressure vessels	$N_{pv,1}$	30	30	14	23
	Second-stage RO system	Inlet transmembrane pressure	ΔP_1 (bar)	---	56.7	57.5	58.9
		Number of modules per pressure vessel	$n_{mem,2}$	---	4	4	4
		Number of pressure vessels	$N_{pv,2}$	---	17	9	15
	Module parameters	Number of feed spacers per element	n_{sp}	23	19	23	11
	Membrane properties	Water permeability	L_p (lmh bar^{-1})	1.00	15.73	25.34	26.23
		Salt permeability	B (lmh)	0.05	0.48	0.79	1.01
Computational results	Average permeate salinity		$\bar{w}_p$ (ppm)	145	495	498	499
	Average water flux		$\bar{J}_w$ (lmh)	19	52	88	111
	Maximum CPF		Max (CPF)	1.09	1.16	1.20	1.20
	Recovery rate		$R_{r,0}$	0.50	0.50	0.50	0.50
	Membrane area		A_{tot} (m^2)	7,804	2,886	1,709	1,351
	Specific energy consumption		SEC (kWh m^{-3})	2.30	1.68	1.78	1.88

Table S8. Optimized two-stage ultrapermeable membrane systems with a limited concentration polarization (no more than 1.25). The analysis is based on a feed salinity of 35,000 ppm, a water recovery rate of 50%, pump efficiency of 85%, and energy recovery efficiency of 95%. The original results are from a conventional one-stage SWRO system with a commercial membrane module under standard engineering conditions. In contrast, optimized results are obtained from a two-stage SWRO system using ultrapermeable membranes and optimized spacer configurations for tradeoff parameter c_m values (40, 220, and 400 \$ m^{-2}), with a maximum concentration polarization factor of 1.25. The geometric specifications of the commercial membrane module, with a 28 mil feed spacer, are detailed in previous work.[10]

Parameters				Value			
				Original	Optimized c_m = 40 \$ m^{-2}	c_m = 220 \$ m^{-2}	c_m = 400 \$ m^{-2}
Design parameters	Geometrical parameters of feed spacers		L_1 (μm)	---	750	750	750
			W_1 (μm)	---	188	188	188
			H_1 (μm)	---	11	11	11
			α (°)	---	31.6	31.6	31.6
			L_S (μm)	---	598	598	598
			W_S (μm)	---	301	301	301
	First-stage RO system	Inlet transmembrane pressure	ΔP_0 (bar)	65.0	40.9	42.2	43.3
		Number of modules per pressure vessel	$n_{mem,1}$	7	4	4	4
		Number of pressure vessels	$N_{pv,1}$	30	29	22	8
	Second-stage RO system	Inlet transmembrane pressure	ΔP_1 (bar)	---	56.7	57.5	58.1
		Number of modules per pressure vessel	$n_{mem,2}$	---	4	4	4
		Number of pressure vessels	$N_{pv,2}$	---	16	13	5
	Module parameters	Number of feed spacers per element	n_{sp}	23	22	14	30
	Membrane properties	Water permeability	L_p (lmh bar^{-1})	1.00	13.68	34.90	42.33
		Salt permeability	B (lmh)	0.05	0.44	0.76	1.06
Computational results	Average permeate salinity		$\bar{w}_p$ (ppm)	145	500	441	495
	Average water flux		$\bar{J}_w$ (lmh)	19	47	95	119
	Maximum CPF		Max (CPF)	1.09	1.15	1.22	1.25
	Recovery rate		$R_{r,0}$	0.50	0.5	0.50	0.50
	Membrane area		A_{tot} (m^2)	7,804	3,199	1,583	1,260
	Specific energy consumption		SEC (kWh m^{-3})	2.30	1.67	1.78	1.86

Table S9. Optimized two-stage ultrapermeable membrane systems with a limited concentration polarization (no more than 1.30). The analysis is based on a feed salinity of 35,000 ppm, a water recovery rate of 50%, pump efficiency of 85%, and energy recovery efficiency of 95%. The original results are from a conventional one-stage SWRO system with a commercial membrane module under standard engineering conditions. In contrast, optimized results are obtained from a two-stage SWRO system using ultrapermeable membranes and optimized spacer configurations for tradeoff parameter c_m values (40, 220, and 400 \$ m^{-2}), with a maximum concentration polarization factor of 1.30. The geometric specifications of the commercial membrane module, with a 28 mil feed spacer, are detailed in previous work.[10]

**Parameters**				**Value**			
				Original	Optimized c_m = 40 \$ m^{-2}	c_m = 220 \$ m^{-2}	c_m = 400 \$ m^{-2}
Design parameters	Geometrical parameters of feed spacers		L_1 (μm)	---	750	750	750
			W_1 (μm)	---	188	188	188
			H_1 (μm)	---	11	11	11
			α (°)	---	31.6	31.6	31.6
			L_S (μm)	---	598	598	598
			W_S (μm)	---	301	301	301
	First-stage RO system	Inlet transmembrane pressure	ΔP_0 (bar)	65.0	41.0	42.8	43.4
		Number of modules per pressure vessel	$n_{mem,1}$	7	4	4	4
		Number of pressure vessels	$N_{pv,1}$	30	25	20	24
	Second-stage RO system	Inlet transmembrane pressure	ΔP_1 (bar)	---	56.8	57.4	59.1
		Number of modules per pressure vessel	$n_{mem,2}$	---	4	4	4
		Number of pressure vessels	$N_{pv,2}$	---	14	14	14
	Module parameters	Number of feed spacers per element	n_{sp}	23	24	14	10
	Membrane properties	Water permeability	L_p (lmh bar^{-1})	1.00	15.90	62.42	59.05
		Salt permeability	B (lmh)	0.05	0.36	0.22	0.43
Computational results	Average permeate salinity		$\bar{w}_p$ (ppm)	145	394	129	200
	Average water flux		$\bar{J}_w$ (lmh)	19	50	98	123
	Maximum CPF		Max (CPF)	1.09	1.16	1.30	1.29
	Recovery rate		$R_{r,0}$	0.50	0.50	0.50	0.50
	Membrane area		A_{tot} (m^2)	7,804	3,025	1,538	1,228
	Specific energy consumption		SEC (kWh m^{-3})	2.30	1.68	1.79	1.88

Table S10. Repeatability test of system design. The analysis is based on a feed salinity of 35,000 ppm, a water recovery rate of 50%, pump efficiency of 85%, and energy recovery efficiency of 95%. Optimized system performance and repeatability are evaluated in a two-stage seawater reverse osmosis (SWRO) using ultrapermeable membranes and optimized spacer configurations for tradeoff parameter c_m values of 310, 340, 370 and 400 \$ m^{-2}, with a maximum concentration polarization factor of 1.25.

c_m (\$ m^{-2})	SEC (kWh m^{-3})		Relative deviation	$\bar{J}_w$ (lmh)		Relative deviation	$\bar{w}_p$ (ppm)		Relative deviation
	Optimized	Repeatability test		Optimized	Repeatability test		Optimized	Repeatability test	
310	1.84	1.82	0.8%	112.2	105.5	5.9%	490	480	2.1%
340	1.82	1.84	1.3%	107.3	114.6	6.8%	500	490	2.0%
370	1.88	1.85	1.3%	121.2	117.2	3.3%	499	497	0.4%
400	1.86	1.88	1.1%	119.2	122.5	2.8%	495	499	0.8%

Table S11. Comparison of normalized SEC (NSEC) breakdowns for batch reverse osmosis.[10]

NSEC = SEC/π_0 (π_0 is feed osmotic pressure)

Contributing terms in NSEC	Batch RO
Thermodynamics, $NSEC_1$	$-\ln\left(1-Y_{\text{tot}}\right)/Y_{\text{tot}}$
Design flux, $NSEC_2$	$\bar{J}_{\text{W}}/\left(L_{\text{P}}\pi_0\right)$
Flow resistance, $NSEC_3$	$\dfrac{\alpha_2}{\pi_0}\dfrac{\dfrac{1}{t_1+1}\dfrac{1-\left(1-Y_{\text{SP}}\right)^{t_1+1}}{Y_{\text{SP}}}+\dfrac{Y_{\text{SP}}\left(1-Y_{\text{tot}}\right)}{Y_{\text{tot}}}}{Y_{\text{SP}}}$
Concentration polarization, $NSEC_4$	$\left(\text{CPF}-1\right)\left[-\dfrac{\ln\left(1-Y_{\text{tot}}\right)}{Y_{\text{tot}}}-\ln\left(1-Y_{\text{tot}}\right)\left(\dfrac{1}{f_{n_{\text{mem}}}}-1\right)\right]$
Salt retention, $NSEC_5$	$-\ln\left(1-Y_{\text{tot}}\right)/\left(1/f-1\right)$
Pump inefficiency, $NSEC_6$	$\left(1/\eta_{\text{pump}}-1\right)\sum_{i=1}^{5}\text{NSEC}_i$

Table S12. Batch ultrapermeable membrane systems with optimized spacer. The analysis is based on a feed salinity of 35,000 ppm, a water recovery rate of 50%, pump efficiency of 85%. The original results are from a conventional one-stage SWRO system utilizing a commercial membrane module under standard engineering conditions. In contrast, optimized results are obtained from a batch SWRO system incorporating ultrapermeable membranes and optimized spacer configurations. The geometric specifications of the commercial membrane module, with a 28 mil feed spacer, are detailed in previous work.[10]

**Parameters**				**Value**			
				Original	Optimized spacer + UPM + batch		
					Option 1	Option 2	Option 3
Design parameters	Geometrical parameters of feed spacers		L_1 (μm)	---	750	750	750
			W_1 (μm)	---	188	188	188
			H_1 (μm)	---	11	11	11
			α (°)	---	31.6	31.6	31.6
			L_S (μm)	---	598	598	598
			W_S (μm)	---	301	301	301
	RO system	Inlet transmembrane pressure	ΔP_0 (bar)	65.0	Varying as a function of time[11]		
		Number of modules per pressure vessel	n_{mem}	7	2	2	2
		Number of pressure vessels	N_{pv}	30	70	30	25
	Module parameters	Number of feed spacers per element	n_{sp}	23	14	14	14
	Membrane properties	Water permeability	L_p (lmh bar^{-1})	1.00	34.90	34.90	34.90
Computational results	Average water flux		$\bar{J}_w$ (lmh)	19	95	221	265
	Maximum CPF		Max (CPF)	1.09	1.14	1.21	1.23
	Recovery rate		$R_{r,0}$	0.50	0.50	0.50	0.50
	Membrane area		A_{tot} (m^2)	7,804	1,583	679	565
	Specific energy consumption		SEC (kWh m^{-3})	2.30	1.68	2.04	2.18

Table S13. Batch ultrapermeable membrane systems with commercial spacer. The analysis is based on a feed salinity of 35,000 ppm, a water recovery rate of 50%, pump efficiency of 85%. The original results are from a conventional one-stage SWRO system utilizing a commercial membrane module under standard engineering conditions. In contrast, optimized results are obtained from a batch SWRO system incorporating ultrapermeable membranes and commercial spacer configurations. The geometric specifications of the commercial membrane module, with a 28 mil feed spacer, are detailed in previous work.[10]

**Parameters**				**Value**			
				Original	Commercial spacer + UPM + batch		
					Option 1	Option 2	Option 3
Design parameters	RO system	Inlet transmembrane pressure	ΔP_0 (bar)	65.0	Varying as a function of time[11]		
		Number of modules per pressure vessel	n_{mem}	7	1	1	1
		Number of pressure vessels	N_{pv}	30	42	18	15
	Module parameters	Number of feed spacers per element	n_{sp}	23	23	23	23
	Membrane properties	Water permeability	L_p (lmh bar^{-1})	1.00	34.90	34.90	34.90
Computational results	Average water flux		$\bar{J}_w$ (lmh)	19	96	224	269
	Maximum CPF		Max (CPF)	1.09	1.26	1.42	1.46
	Recovery rate		$R_{r,0}$	0.50	0.50	0.50	0.50
	Membrane area		A_{tot} (m^2)	7,804	1,561	669	557
	Specific energy consumption		SEC (kWh m^{-3})	2.30	1.85	2.30	2.45

Supplementary References

1. Ruan X., Jiang P., Zhou Q., et al. (2020). Variable-fidelity probability of improvement method for efficient global optimization of expensive black-box problems. *Structural and Multidisciplinary Optimization* **62**:3021-3052. DOI:10.1007/s00158-020-02646-9
2. Jones D. R., Schonlau M. and Welch W. J. (1998). Efficient global optimization of expensive black-box functions. *Journal of Global Optimization* **13**:455–492. DOI:10.1023/A:1008306431147
3. Blockeel H., Kristian K., Nijssen S., et al. (2013). Machine learning and knowledge discovery in databases (Springer). 10.1007/978-3-642-40988-2.
4. Bull A. D. (2011). Convergence rates of efficient global optimization algorithms. *Journal of Machine Learning Research* **12**:2879-2904. DOI:10.5555/1953048.2078198
5. Li M. H., Bui T. and Chao S. (2016). Three-dimensional CFD analysis of hydrodynamics and concentration polarization in an industrial RO feed channel. *Desalination* **397**:194-204. DOI:10.1016/j.desal.2016.07.005
6. Guillen G. and Hoek E. M. V. (2009). Modeling the impacts of feed spacer geometry on reverse osmosis and nanofiltration processes. *Chem. Eng. J.* **149**:221–231. DOI:10.1016/j.cej.2008.10.030
7. Li M. H. (2021). Residence time distribution in RO channel. *Desalination* **506**:115000. DOI:10.1016/j.desal.2021.115000
8. Luo J., Li M., Hoek E. M. V., et al. (2023). Supercomputing and machine learning-aided optimal design of high permeability seawater reverse osmosis membrane systems. *Sci. Bull.* **68**:397-407. DOI:10.1016/j.scib.2023.01.039
9. Geraldes V. and Afonso M. D. (2006). Generalized mass-transfer correction factor for nanofiltration and reverse osmosis. *AIChE J.* **52**:3353-3362. DOI:10.1002/aic.10968
10. Bucs S. S., Radu A. I., Lavric V., et al. (2014). Effect of different commercial feed spacers on biofouling of reverse osmosis membrane systems: a numerical study. *Desalination* **343**:26–37. DOI:10.1016/j.desal.2013.11.007
11. Li M. H. (2020). Effects of finite flux and flushing efficacy on specific energy consumption in semi-batch and batch reverse osmosis processes. *Desalination* **496**:114646. DOI:10.1016/j.desal.2020.114646
